# Genome-wide analysis of complex wheat gliadins, the dominant carriers of celiac disease epitopes

**DOI:** 10.1038/srep44609

**Published:** 2017-03-16

**Authors:** Da-Wei Wang, Da Li, Junjun Wang, Yue Zhao, Zhaojun Wang, Guidong Yue, Xin Liu, Huanju Qin, Kunpu Zhang, Lingli Dong, Daowen Wang

**Affiliations:** 1 The State Key Laboratory of Plant cell and Chromosome Engineering, Institute of Genetics and Developmental Biology, Chinese Academy of Sciences, Beijing, China; 2 University of Chinese Academy of Sciences, Beijing, China; 3 Tobacco Research Institute, Chinese Academy of Agricultural Sciences, Qingdao, China; 4 The Collaborative Innovation Center for Grain Crops, Henan Agricultural University, Zhengzhou, China

## Abstract

Gliadins, specified by six compound chromosomal loci (*Gli-A1/B1/D1* and *Gli-A2/B2/D2*) in hexaploid bread wheat, are the dominant carriers of celiac disease (CD) epitopes. Because of their complexity, genome-wide characterization of gliadins is a strong challenge. Here, we approached this challenge by combining transcriptomic, proteomic and bioinformatic investigations. Through third-generation RNA sequencing, full-length transcripts were identified for 52 gliadin genes in the bread wheat cultivar Xiaoyan 81. Of them, 42 were active and predicted to encode 25 α-, 11 γ-, one δ- and five ω-gliadins. Comparative proteomic analysis between Xiaoyan 81 and six newly-developed mutants each lacking one *Gli* locus indicated the accumulation of 38 gliadins in the mature grains. A novel group of α-gliadins (the CSTT group) was recognized to contain very few or no CD epitopes. The δ-gliadins identified here or previously did not carry CD epitopes. Finally, the mutant lacking *Gli-D2* showed significant reductions in the most celiac-toxic α-gliadins and derivative CD epitopes. The insights and resources generated here should aid further studies on gliadin functions in CD and the breeding of healthier wheat.

Wheat is a major staple crop in the world, with a total of 728.967 million metric tons of grains produced in 2014^1^. Wheat foods are consumed globally and supply approximately 20% of the calories and protein that humans consume^2^. However, despite intensive breeding efforts in the past, there still exist many undesirable substances in wheat grains and derived foods, whose consumption can induce severe illnesses in sensitive human individuals^3^,^4^. Comprehensive characterization of these compounds is a prerequisite for effectively decreasing their contents in the development of healthier wheat foods through innovative plant breeding^5^.

Gliadins, a distinct family of gluten proteins, are strongly accumulated in wheat grains^6^,^7^,^8^. In hexaploid bread wheat (*Triticum aestivum*, 2n = 6x = 42, AABBDD), which accounts for over 95% of global wheat production^9^, gliadins constitute approximately 40–50% of total grain proteins^6^,^7^,^8^. Gliadins have been divided into α-, γ-, δ- and ω-types based on differences in the primary structure and the presence and number of conserved cysteine residues in the protein^6^,^7^,^8^. During wheat food processing, gliadins and two other families of gluten proteins, i.e., high-molecular-weight glutenin subunits (HMW-GSs) and low-molecular-weight glutenin subunits (LMW-GSs), form a gluten complex^6^,^7^,^8^. Thus, gliadins have traditionally and more frequently been studied for their effects on the functional properties of gluten complex and the end-use quality of wheat grains^6^,^7^,^8^. However, in the human gastrointestinal track, many gluten proteins are poorly digested, and the resultant peptides, especially those derived from gliadins, have been linked with the elicitation of celiac disease (CD)^10^. CD has been considered as one of the most important food related disorders in the world, because 1) it affects 1–3% of human population, and 2) the incidence of CD is rising in many developing countries in association with increased consumption of wheat based foods^5^,^10^,^11^. Furthermore, due to the involvement of similar genetic predispositions, the incidence of CD is substantially higher among the individuals with type 1 diabetes, and CD can acerbate the detrimental effects of type 1 diabetes^12^. Together, these factors have prompted extensive international studies on CD, which have revealed the central importance of gliadins in the cause and pathogenesis of CD^13^.

In bread wheat, the genes encoding gliadins are contained mainly in six compound loci, with *Gli-A1, -B1* and *-D1* located on the short arms of group 1 chromosomes (1AS, 1BS and 1DS) and *Gli-A2, -B2* and *-D2* on the short arms of group 6 chromosomes (6AS, 6BS and 6DS)^6^,^7^,^8^. Moreover, *Gli-A1, -B1* and *-D1* are, respectively, linked with the *Glu-A3, -B3* and *-D3* loci that specify LMW-GSs^6^,^7^,^8^. Orthologous *Gli* loci are present in the diploid grasses *T. urartu* and *Aegilops tauschii*, which donated the A and D genomes to bread wheat, respectively, and the tetraploid wheat *T. turgidum*, which is involved in the hexaploidization event yielding hexaploid wheat^6^,^7^,^8^,^14^. The genes coding for γ-, ω- or δ-gliadins are carried in *Gli-1* loci, whereas those for α-gliadins are in *Gli-2* loci^6^,^7^,^8^. There are frequently highly similar homoeologous and paralogous gliadin members, and many gliadins have multiple allelic variants among different wheat cultivars^6^,^7^,^8^. Apart from the six major *Gli* loci outlined above, several minor gliadin loci have been reported to locate on 1AS (*Gli-A3, -A5* and *-A6*), 1BS (*Gli-B3* and *-B5*) and 1DS (*Gli-D4* and *-D6*)^6^. In addition to carrying the genes for α-gliadins, *Gli-2* loci have also been suggested to harbor the genes encoding β-gliadins that migrated slower than α-gliadins in the electrophoresis under low pH^7^. But later studies revealed that the two types of gliadins were very similar in amino acid sequence and could be collectively named as α-gliadins^7^.

To date, none of the six *Gli* loci of bread wheat has been completely sequenced. A partial sequencing of bread wheat *Gli-A2, -B2* and *-D2* loci, approximately 200 kb for each locus, has been reported, with 12 active and four inactive α-gliadin genes identified^15^. A recent study has sequenced a chromosomal region harboring the entire *Gli-D*^*t*^*1* locus of *Aegilops tauschii*, which is orthologous to *Gli-D1*^16^. In *Gli-D*^*t*^*1*, six ω-gliadin, four γ-gliadin and two δ-gliadin genes are found in a genomic DNA segment of about 888.5 kb, and for all three types of gliadin genes, there are both active and pseudogene members. A proteomic approach, involving digestion of excised protein spots separated by two-dimensional gel electrophoresis (2-DE) with multiple proteases, has been proposed for identifying and differentiating similar gliadins accumulated in bread wheat grains^17^,^18^. Attempts have also been made to match the gliadins identified by tandem mass spectrometry (MS/MS) to gliadin gene coding sequences constructed with expressed sequence tags (ESTs)^17^,^18^. But many of the gliadin gene coding sequences assembled using ESTs were incomplete, which compromised accurate matching between gliadin proteins and transcripts present in the grains^17^,^18^. A transcriptomic analysis of the genes expressed in developing wheat seeds using second generation sequencing (SGS) technology has identified multiple gliadin transcripts and genes^19^. However, the gliadin gene sequences constructed with SGS reads may be error-prone because the presence of highly repetitive nucleotide sequence elements in these genes can cause misassembly of the reads from similar homoeologous or paralogous members. In the recently published draft genome sequence for the spring wheat line Chinese Spring (CS), the chromosomal regions carrying *Gli* loci are largely covered by short contigs^20^. Moreover, CS is unlikely useful for genome-wide analysis of gliadin genes because of lacking *Gli-D2* locus due to a natural deletion on chromosome 6D^19^,^20^. Clearly, gliadins, especially those in bread wheat, are highly heterogeneous and complex. The progress in genome-wide characterization of bread wheat gliadins and their corresponding genes has been slow. Comprehensive characterization of gliadin proteins and their corresponding genes expressed in bread wheat varieties remains a strong challenge.

The gliadin peptides that promote CD generally harbor one or more epitopes capable of binding to human T cells^21^,^22^,^23^. Such epitopes are also present in HMW-GSs and LMW-GSs but in a reduced amount^5^,^10^. The epitopes are generally rich in proline and glutamine, and the high content of proline renders them resistant to protease digestion^21^,^22^,^23^. These epitopes bind to specific haplotypes of human leucocyte antigen (HLA) class II proteins, i.e., HLA-DQ2.2, HLA-DQ2.5, HLA-DQ8 and HLA-DQ8.5, expressed on the surface of CD4^+^ T cells. This elicits complex biochemical and cell biological events, leading to epithelial cell destruction and villous atrophy in the small intestine^21^,^22^,^23^. So far, a total of 31 epitopes involved in CD have been compiled based on available *in vitro* and *in vivo* evidence, the majority of which are derived from gliadins^5^,^10^. Bioinformatic analysis of protein sequences has frequently been employed to detect the presence of CD epitopes in different gliadins. The results show that different types of gliadins differ substantially in the CD epitopes contained, with the α-gliadins specified by *Gli-B2* containing very few CD epitopes^5^,^10^,^24^,^25^,^26^,^27^. However, this type of analysis has not been reported for δ-gliadins, which are recognized only recently^28^,^29^, nor has it been conducted for all of the gliadins accumulated in a single bread wheat variety.

Based on the information presented above, we aimed to develop an experimental model for comprehensively characterizing the gliadins expressed in bread wheat. Because of the high complexity of gliadins in bread wheat, multidisciplinary investigations combining transcriptomic, proteomic, mutagenetic and bioinformatic approaches were used. Important to our efforts was the preparation of six unique mutants each lacking one of the six *Gli* loci in our model variety Xiaoyan 81, an elite winter type bread wheat cultivar^30^. To efficiently identify the mRNAs of transcribed gliadin genes, the newly emerged third generation sequencing platform PacBio RSII, which is effective for obtaining full-length transcript sequences^31^,^32^,^33^,^34^,^35^, was adopted. For differentiating closely related gliadin proteins, the 2-DE-MS/MS based proteomic approach, previously recommended for gliadin identification^17^,^18^, was employed. The data generated allowed us to identify the spectrum of gliadin genes expressed through matching full-length transcripts to their protein products, to assign the expressed gliadin genes to individual *Gli* loci, and to bioinformatically assess the presence of CD epitopes in all of the gliadin proteins accumulated in the grains of a bread wheat cultivar.

## Results

### Analysis of full-length transcripts of gliadins and their corresponding genes

Three RNA sequencing experiments (RSE1, 2 and 3) were conducted to investigate the full-length transcripts of gliadins. RSE1 used total RNAs extracted from a pooled sample of the grains collected at 0, 5, 15 and 25 days after flowering (DAF), and was accomplished with PacBio RSII platform. RSE2 was conducted with Illumina HiSeq 2000 platform, and sequenced RNAs in the grains at 0, 5, 15 and 25 DAF, respectively. RSE3 was carried out with PacBio RSII, and used total RNAs from the grains collected at 25 DAF. The high-quality reads produced in RSE2 were employed to correct nucleotide errors in the PacBio transcriptomic reads obtained in RSE1 and RSE3 as described previously^36^,^37^. A foregoing analysis of the RSE1 data showed that the majority of the full-length non-chimeric (FLNC) reads, approximately 74.6%, were likely to contain full-length open reading frame (ORF), and by searching RSE1 FLNC reads, 72 unique full-length transcripts were identified for wheat gluten genes, including six for HMW-GSs, 14 for LMW-GSs and 52 for gliadins^38^. Among the full-length transcripts of gliadins, 32 were transcribed from α-gliadin genes, 13 from γ-gliadin genes, one from δ-gliadin gene, and six from ω-gliadin genes.

To verify the number of full-length transcripts found for gluten genes by RSE1, a total of 187,279 FLNC reads, produced in RSE3 (Table S1), were analyzed. After removal of redundancy, 41,611 unique transcripts were obtained, 35,380 of which could be mapped to the draft genome sequence of CS; the 35,380 transcripts covered 14,747 extant chromosomal loci and 5,449 new loci (not annotated by the draft genome sequence of CS) (Table S1). Searching the RSE3 transcriptomic data yielded an identical set of full-length transcripts for gluten genes as that based on RSE1 data. Thus, the 52 unique full-length transcripts for gliadins were supported by two independent PacBio RNA sequencing experiments. Of the 52 transcripts, 42 had intact coding sequence, and the remaining 10 had defective coding region owing to the presence of premature stop codon or frame shift mutations (Table S2). Here, we focused on studying the gliadins specified by the 42 full-length transcripts having intact coding region, with their corresponding genes tentatively named to facilitate more detailed analysis (Table 1). The chromosomal locations of the genes yielding the 42 gliadin transcripts were determined with the aid of six deletion lines lacking each of the six *Gli* loci and by PCR mapping (see below). For the 10 transcripts with disrupted coding sequence, they were transcribed from seven α-, two γ- and one ω-gliadin pseudogenes, respectively (Table S2).

Among the 25 transcribed, intact α-gliadin genes, eight members came from chromosome 6A, 10 from chromosome 6B, and seven from chromosome 6D (Table 1). In general, their transcript levels were upregulated during grain development, although those of *Gli-α7, Gli-α8, Gli-α18* and *Gli-α25* were comparatively lower than their respective counterparts from the same chromosome (Figure S1). The length of the proteins deduced from the 25 members ranged from 286 to 325 residues (Figure S2), and their sequence identities varied from 75.7% (between Gli-α9 and Gli-α20) to 99.7% (between Gli-α1 and Gli-α2, Gli-α11 and Gli-α12, Gli-α13 and Gli-α14, or Gli-α15 and Gli-α16). Their primary structure was identical to that previously defined for α-gliadins^39^, being composed of a signal peptide, a N-terminal repetitive region, two regions containing polyglutamine, and two unique regions carrying conserved cysteine residues (Figure S2). The number of conserved cysteine residues was six, with one additional cysteine found in Gli-α25 (Figure S2). Interestingly, the 25 α-gliadins could be divided into two groups based on amino acid variation downstream of the last conserved cysteine residue. The CSTT group had 12 members, including Gli-α8 specified by chromosome 6A, Gli-α9 to Gli-α18 by chromosome 6B, and Gli-α19 by chromosome 6D, whereas the CT group contained the remaining 13 members by either 6A (Gli-α1 to Gli-α7) or 6D (Gli-α20 to Gli-α25) chromosomes (Figure S2). Members in the CSTT group also exhibited unique amino acid substitutions in the first polyglutamine region (PQR1) and the first unique region (UR1) (Figure S2).

The 11 transcribed, intact γ-gliadin genes resided on chromosomes 1A (*Gli-γ1* to *Gli-γ3*), 1B (*Gli-γ4* to *Gli-γ7*) or 1D (*Gli-γ8* to *Gli-γ11*) (Table 1). Their transcript levels were generally increased as grain development progressed (Figure S3). The primary structure of the 11 deduced γ-gliadins was identical to that previously reported for this type of proteins^40^,^41^, which was consisted of a signal peptide (SP), a short N-terminal region (NR), a repetitive region (RR), a region with polyglutamine (PQR), and two unique regions (UR1 and UR2) containing the conserved cysteine residues (Figure S4). The number of conserved cysteine residues was eight, with one extra cysteine found in Gli-γ5 and Gli-γ10, respectively (Figure S4). The length of the 11 deduced γ-gliadins ranged from 285 to 357 residues (Figure S4), and their sequence identities varied from 66.7% to 100%. For the two members (Gli-γ2 and Gli-γ3) that were 100% identical, their coding regions (from the start to stop codons) differed by two synonymous nucleotide substitutions.

The δ-gliadins are recognized very recently, and one to two active genes have been identified for δ-gliadins in two common wheat varieties (CS and Hereward)^28^,^29^. In this work, we found the full-length transcript for only one unique and intact δ-gliadin gene (*Gli-δ1*, Table 1) in our experimental variety Xiaoyan 81. The transcript level of *Gli-δ1* was up-regulated during grain development (Figure S5a), and its deduced protein was highly similar to the two δ-gliadins of CS (Figure S5b). The main primary structural features, including a signal peptide, a short N-terminal region, a repetitive region, and a unique region with eight cysteine residues, were shared among the three compared δ-gliadins.

The ω-gliadins have been designated according to the first four amino acid residues of the N-terminal region^42^,^43^. The ω-gliadins specified by chromosomes 1A or 1D are generally AREL- and ARQL-types, while those by chromosomes 1B are usually SRLL-type^42^,^43^. There is also an alternative classification scheme that divides these gliadins into two subgroups, ω5-gliadins and ω1, 2-gliadins^43^. In general, the ω5-gliadins have a N-terminus starting with SRL and carry the repetitive motifs FPQQQ and QQIPQQ, while the ω1, 2-gliadins possess a N-terminus starting with ARE, ARQ or KEL and have the repetitive element PQQPFP. Here, we identified five transcribed, intact ω-gliadin genes, coming from chromosomes 1A (*Gli-ω1*), 1B (*Gli-ω2* and *Gli-ω3*) and 1D (*Gli-ω4* and *Gli-ω5*), respectively (Table 1). The transcript levels of the five ω-gliadin genes were generally increased as grain development proceeded (Figure S6). Consistent with previous studies, we noticed that the ω-gliadins by 1A or 1D were either ARQL-type (Gli-ω1 and -5) or AREL-type (Gli-ω4), and that one of the 1B ω-gliadin (Gli-ω3) belonged to the SRLL-type (Figure S7a,b). In contrast, the other 1B ω-gliadin, Gli-ω2, had a novel N-terminus starting with ARPL, and exhibited high similarity to a previously reported D-type glutenin protein (Figure S7c). The deduced Gli-ω2 and Gli-ω5 proteins each contained a cysteine residue in the repetitive region, but no such residue was observed in Gli-ω1, -ω3 or -ω4 (Figure S7). Gli-ω1, -4 and -5 belonged to ω1, 2-gliadins because their N-terminus started with ARQ or ARE and they carried the repetitive motif PQQPFP; Gli-ω3 was a typical ω5-gliadin because its N-terminus began with SRL and it carried the repetitive motifs FPQQQ and QQIPQQ (Figure S7a,b). Gli-ω2 was likely a ω1, 2-gliadin because the first three residues of its N-terminus (ARP) resembled ARE and ARQ, and it had the repetitive motif PQQPFP (rather than the FPQQQ or QQIPQQ elements normally found in ω5-gliadins) (Figure S7c).

Among the four types of genes analyzed above, the transcript levels of γ-gliadin genes were comparatively high, followed by those of α-gliadin genes; the transcript levels of δ- and ω-gliadin genes were relatively low except for *Gli-ω4* (Figures S1, S3, S5 and S6). This gene was substantially more highly transcribed than other ω-gliadin gene members (Figure S6).

### Development of six deletion lines lacking individual *Gli* loci

Our previous study has shown that a mutant population of Xiaoyan 81 created by ion beam mutagenesis is useful for isolating the deletion mutants of important wheat chromosomal loci^30^. Thus, in this work, we screened this mutant population for isolating the deletion mutants lacking individual *Gli* loci (*Gli-A1, -B1, -D1, -A2, -B2* or *-D2*) by MALDI-TOF-MS analysis. For Xiaoyan 81, 10 compound gliadin peaks (1^#^ to 10^#^) in the mass range of 28–40 kD were consistently obtained (Figure S8a). A similar set of MS peaks was observed for the gliadin extract of CS (Figure S8a). Because it is known that *Gli-A1, -B1, -D1, -A2, -B2* and *-D2* are located on chromosomes 1A, 1B, 1D, 6A, 6B and 6D, respectively^6^,^7^,^8^, we examined the gliadin MS peaks of the six nulli-tetrasomic lines of CS each lacking one of the six chromosomes. This permitted us to link the 10 gliadin MS peaks with the *Gli* locus on 1A, 1B, 1D, 6A, 6B or 6D chromosomes (Figure S8b). Subsequently, we screened the gliadin extracts of 10,100 M2 seeds (five from each of the 2020 M2 families), and a total of 78 M_2_ families were found to miss one or more of the 10 gliadin MS peaks. Eventually, six unique and homozygous deletion lines (DLGliA1, DLGliB1, DLGliD1, DLGliA2, DLGliB2 and DLGliD2), lacking *Gli-A1, -B1, -D1, -A2, -B2* and *-D2*, respectively, were developed (Table 2). Examination with chromosome specific microsatellite markers revealed that the missing of individual *Gli* loci in the six mutant lines was generally caused by large genomic deletion in 1A, 1B, 1D, 6A, 6B or 6D chromosomes (Figure S9). The microsatellite markers missed in the six mutant lines provided a convenient means for their identification (Table 2). DLGliA1, DLGliB1, DLGliD1, DLGliA2 and DLGliD2 were fertile with normal seed development; DLGliB2 was partially infertile but the selfed seeds developed and germinated normally (Figure S10).

### Proteomic analysis of gliadins in Xiaoyan 81 and *Gli* locus deletion lines

**Figure 1:**
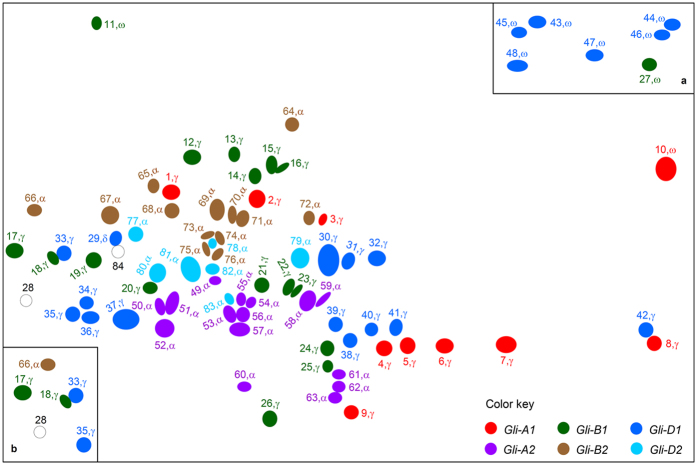
A schematic representation of the 2-DE spots resolved for the gliadin extract of Xiaoyan 81 mature grains. The spots were numbered (1 to 84) and the types of gliadins (α, γ, δ or ω) to which they belonged were indicated. They were also color-coded to indicate the control by individual *Gli* loci (i.e., *Gli-A1, -B1, -D1, -A2, -B2* and *-D2*), which was determined by comparing the 2-DE data of Xiaoyan 81 with those of the six *Gli* locus deletion lines (DLGliA1, DLGliB1, DLGliD1, DLGliA2, DLGliB2 and DLGliD2). The majority of the spots were well separated by IEF under pH 6–11, but those shown in insets a and b were better resolved by IEF under pH 3–10 (Figure S12). Two spots (28 and 84) could not be resolved because both were mixtures of different types of gliadins. The image was prepared based on the data of 25 independent 2-DE runs (seven for Xiaoyan 81 and three for each of the six deletion lines) and the MS/MS analysis results of the 2-DE spots.

As the first step in this series of experiments, the gliadin extract prepared from the mature grains of Xiaoyan 81 was separated using 2-DE, which involved isoelectric focusing (IEF) and SDS-PAGE in the first and second dimensions, respectively. To maximize the resolving of gliadins, IEF was performed under two pH regimes, one under pH 6–11 and the other under pH 3–10. The majority of the gliadin spots were well separated under pH 6–11 (Figure S11), but IEF under pH 3–10 enabled better separation of approximately a dozen of protein spots with the isoelectric point (pI) ranging from 3 to 6 or 6 to 7 (Figure S12). A total of 97 protein spots were consistently found for Xiaoyan 81 gliadin extract in seven independent 2-DE runs. These spots were excised from the gel, digested by chymotrypsin and thermolysin separately, and examined by nano LC-LTQ-MS/MS, with the resultant mass spectral data analyzed bioinformatically (see Methods). Of the 97 spots, 95 corresponded to individual gliadin (82 spots) or LMW-GS (13 spots) proteins, with the remaining 2 (spots 28 and 84) being mixtures of different gliadins. For the 82 well resolved gliadin spots, they were identified to be α-gliadin (35 spots), γ-gliadin (37 spots), ω-gliadin (9 spots) or δ-gliadin (1 spot) (Fig. 1). To stick to the main objective of this work, the MS data of the 82 gliadin spots are described in Table S3.

Subsequently, the gliadin extracts of the six deletion lines were investigated following the experimental scheme outlined above. The lack of individual *Gli* loci in these lines decreased the number of gliadin spots present in 2-DE. Specifically, for DLGliA1, DLGliB1 and DLGliD1, the numbers of gliadin spots missed were 10, 17 and 20, respectively; in DLGliA2, DLGliB2 and DLGliD2, the numbers of gliadin spots lacked were 15, 13 and 7, respectively (Fig. 1, Table 3). The total number of gliadin spots lacked in the six deletion lines (i.e., 82) equaled to the well resolved gliadin spots found for Xiaoyan 81 (Fig. 1, Table 3). Thus, the 82 gliadin spots could all be assigned to individual *Gli* loci (Table 3). In addition to gliadin spots, DLGliA1, DLGliB1 and DLGliD1 also lacked two, six and five LMW-GS spots, respectively, indicating that the *Glu-A3, -B3* and *-D3* loci specifying LMW-GSs were also deleted in these three mutant lines.

### Matching gliadin spots to active gliadin genes

The MS peptide data of the 82 gliadin spots were searched against the Xiaoyan 81_Gluten database in order to match the 2-DE spots to the 42 intact and transcribed gliadin genes (detailed in Methods). As shown in Table 4 and Table S3, positive match was obtained for 38 active gliadin genes, but not for *Gli-α7, Gli-α8, Gli-α18* and *Gli-α25*. The match was most satisfactory for the single δ-gliadin and 11 γ-gliadin genes, followed by the five ω-gliadin genes. For δ- and γ-gliadin genes, the match was generally supported by unique peptides; *Gli-γ2* and *Gli-γ3* were matched to the same set of 2-DE spots (4–9, Table 4 and Table S3), which was in agreement with the fact that the deduced proteins of the two genes were 100% identical (see above). In the case of ω-gliadin genes, specific matching (supported by unique peptides) was obtained for *Gli-ω1, Gli-ω2* and *Gli-ω3*, with *Gli-ω4* and *Gli-ω5* being matched to the same two spots (43 and 44) and *Gli-ω4* being additionally matched to the spots 45–48 (Table 4 and Table S3). For the 21 α-gliadin genes, specific matching (supported by unique peptides) was achieved for 13 members (i.e., *Gli-α1, -α2, -α3, -α4, -α5, -α6, -α9, -α19, -α20, -α21, -α22, -α23* and *-α24*); for the remaining eight members (*Gli-α10, -α11, -α12, -α13, -α14, -α15, -α16* and *-α17*), the matching was deduced based upon maximal protein coverage by the peptides derived from individual spots (Table 4 and Table S3). *Gli-α1* and *Gli-α2* were matched to the same two spots (50 and 51, Table 4 and Table S3), consistent with the finding that the deduced proteins of the two genes were 99.7% identical (see above). This was also true for *Gli-α11* and *Gli-α12*, with both being matched to the 2-DE spot 66 (Table 4 and Table S3).

For the 38 active gliadin genes with positive 2-DE spot matching (Table 4), they were assigned to different *Gli* loci based on *Gli* locus information of the matched 2-DE spots (Table 3). For *Gli-α7, Gli-α8, Gli-α18* and *Gli-α25*, which did not have positive matching, PCR mapping, aided with gene specific primers and the six *Gli* locus deletion lines, was conducted to assign them to specific *Gli* loci. The results showed that both *Gli-α7* and *Gli-α8* were from *Gli-A2*, with *Gli-α18* and *Gli-α25* being from *Gli-B2* and *Gli-D2*, respectively (Table 4).

PCR mapping was also undertaken to validate *Gli* locus assignment of the active genes based on matched 2-DE spots. We selected 10 α-gliadin (*Gli-α1, -α4, -α5, -α9, -α10, -α11, -α16, -α17, -α20* and *-α24*), two γ-gliadin (*Gli-γ1* and *-γ11*) and one ω-gliadin (*Gli-ω2*) genes as representatives for this validation test. As anticipated, PCR mapping results for the 13 representative genes agreed with their *Gli* locus assignment based on matching 2-DE spots (Table 4). A typical result of the validation test is shown in Figure S13.

### Computation of CD epitopes in gliadin proteins

**Figure 2:**
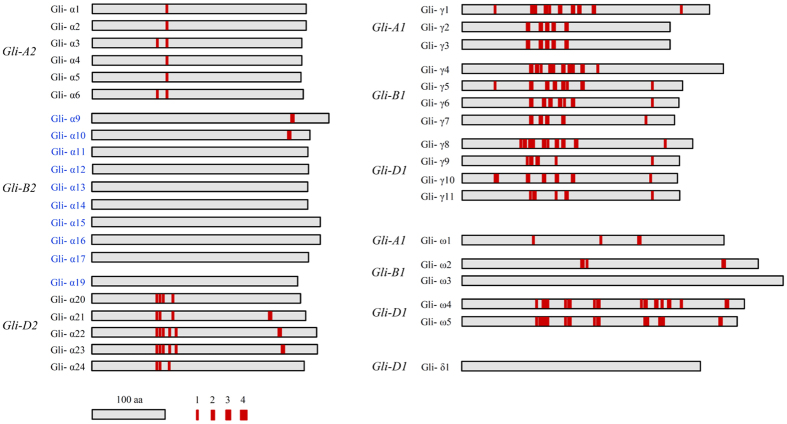
A diagram illustrating the CD epitopes computed for the 38 gliadins accumulated in Xiaoyan 81 mature grains. The six *Gli* loci (*Gli-A1, -B1, -D1, -A2, -B2* and *-D2*) specifying the different gliadins are presented. The four filled red boxes with varying width indicate one to four copies of CD epitopes, respectively. The CSTT α-gliadins (Gli-α9 to α17 and Gli-α19) are marked in blue. The CD epitopes examined are listed in Tables S4 and S5.

The protein sequences of the 38 gliadins accumulated in Xiaoyan 81 mature grains were each searched for the presence of 24 CD epitopes previously found in different gliadins^5^. The results are summarized in Tables S4 and S5, and schematically presented in Fig. 2. The 11 γ-gliadins were found to have more diverse and numerous CD epitopes, followed by the five α-gliadins (Gli-α20 to Gli-α24) specified by *Gli-D2* and four ω-gliadins by *Gli-A1* (Gli-ω1), *Gli-B1* (Gli-ω2) or *Gli-D1* (Gli-ω4 and Gli-ω5); the number of CD epitopes in these 20 gliadins ranged from three to 23. Previous studies have characterized a major celiac-toxic α-gliadin peptide composed of 33 residues and carrying the three epitopes DQ2.5-glia-α1a, DQ2.5-glia-α1b and DQ2.5-glia-α2^44^,^45^. Among the α-gliadins by *Gli-D2*, Gli-α20, -α22 and -α23 carried all of the three epitopes, and Gli-α21 and Gli-α24 harbored two of the three epitopes (Fig. 2 and Tables S4). The remaining 18 gliadins contained fewer or no CD epitopes. Specifically, two epitopes were found in each of the eight α-gliadins by *Gli-A2* (Gli-α1 to Gli-α6) or *Gli-B2* (Gli-α9 and Gli-α10); no epitope was present in the seven α-gliadins (Gli-α11 and Gli-α17) by *Gli-B2*, Gli-α19 by *Gli-D2*, Gli-δ1 by *Gli-D1*, and Gli-ω3 by *Gli-B1* (Fig. 2 and Tables S4 and S5).

To sum up, of the 38 gliadins accumulated in Xiaoyan 81 mature grains, 10 did not carry any CD epitope, eight had one or two epitopes in their proteins, and 20 contained more than three epitopes in their proteins. Interestingly, eight of the 10 accumulated CSTT α-gliadins did not contain CD epitopes, whereas the 11 expressed CT α-gliadins all harbored CD epitopes (Fig. 2 and Table S4). Finally, we noticed that the five δ-gliadins, previously reported for bread wheat and *Ae. tauschii*^28^,^29^, did not contain CD epitope either (Figure S14).

### Investigating the levels of α-gliadins and CD epitopes in DLGliD2

**Figure 3:**
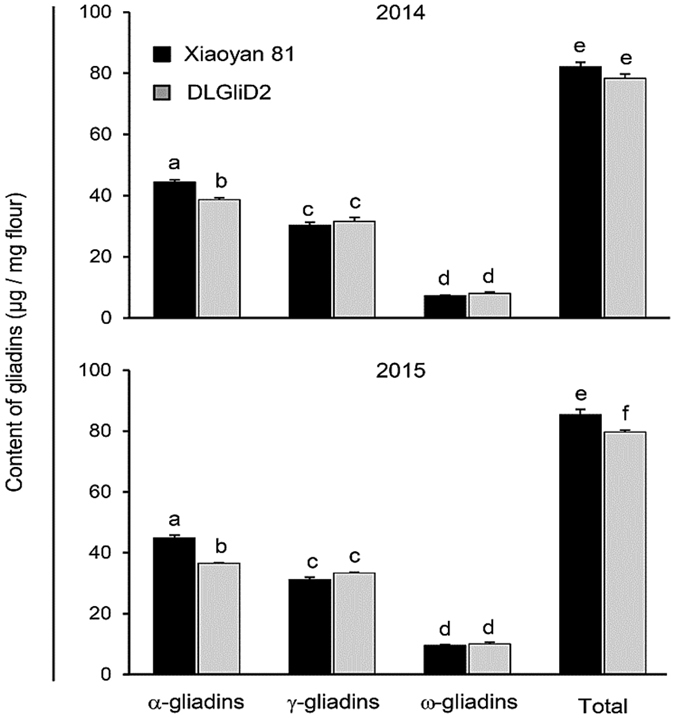
Comparison of gliadin accumulation level in the grains between Xiaoyan 81 and the deletion line DLGliD2 harvested from two different years (2014 and 2015). The gliadins were separated and quantified using reverse phase high performance liquid chromatography, with the resultant values shown as means ± SD. The columns marked by different letters are statistically different (*P* < 0.05, one-way ANOVA with Bonferroni correction).

Because the majority of the α-gliadins encoded by *Gli-D2* carried the highly celiac-toxic epitopes (Fig. 2 and Table S4), the deletion line DLGliD2, which lacked *Gli-D2* and the entire suite of α-gliadins specified by it (Table 3), may be useful for developing wheat lines with reduced celiac toxicity. This led us to examine the levels of α-gliadins and CD epitopes in DLGliD2. The flour samples, milled from the grains of DLGliD2 and its progenitor Xiaoyan 81 harvested from two years (2014 and 2015), were used in this set of experiments. As shown in Fig. 3, for both Xiaoyan 81 and DLGliD2 and in both years, the content of α-gliadins was significantly higher than that of γ-gliadins or ω-gliadins, with the level of ω-gliadins being the lowest. The content of α-gliadins was significantly reduced in DLGliD2 in both years compared to that of Xiaoyan 81, but the levels of γ- and ω-gliadins did not differ considerably between the two lines. The total amount of gliadins in DLGliD2 was also decreased relative to that of Xiaoyan 81, with the decrease reaching the significance level in 2015 (Fig. 3).

**Figure 4:**
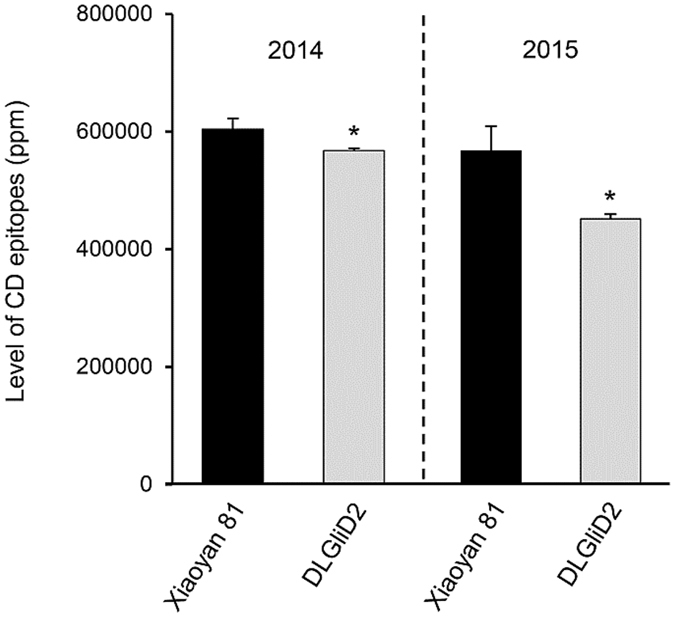
Analysis of CD epitope level in the grains of Xiaoyan 81 and the deletion line DLGliD2 harvested from two different years (2014 and 2015). The level of CD epitopes was determined using the G12 based immunoassay. The values shown are means ± SD. The asterisk indicates statistical significance (*P* < 0.05, independent samples t-test) from the mean of Xiaoyan 81.

The monoclonal antibody G12, raised against the highly celiac-toxic 33-mer peptide, offers an effective means for assessing the level of CD epitopes^46^,^47^. We therefore used a G12 based immunoassay (see Methods) to investigate the level of CD epitopes in DLGliD2. In both 2014 and 2015, the level of CD epitopes was significantly decreased in DLGliD2 relative to that in Xiaoyan 81, with the reduction exhibited by DLGliD2 exceeding 20% in 2015 (Fig. 4).

## Discussion

The high multiplicity and similarity of gliadins and the complexity of bread wheat genome have imposed many difficulties for efficiently dissecting the expression and functionalities of different gliadins. Here, we conducted a genome-wide characterization of bread wheat gliadins and derivative CD epitopes by combining RNA sequencing, proteomic and bioinformatic approaches. The new insights gathered and their implications for future research are discussed below.

### Efficient identification of gliadin genes expressed in bread wheat

Two outstanding questions in the genetic and genomic studies of gliadins are (1) how many gliadin genes exist in the bread wheat genome and (2) how many of them are expressed during grain development. To date, neither of the two questions has been addressed satisfactorily. None of the six *Gli* loci was adequately sequenced and annotated in the draft genome sequence of CS^20^. Although the transcription of multiple gliadin genes in developing wheat grains was noted in a recent transcriptomic study^19^, the *Gli* locus location and protein products of the transcribed gliadin genes were not systematically determined. The combination of EST sequencing and proteomic analysis has improved the understanding on the gliadins expressed in the bread wheat cultivar Butte 86, but the cDNA (mRNA) sequences constructed for different gliadins are still largely incomplete^17^,^18^. None of the eight ω-gliadin gene sequences constructed had a complete coding region; eight α-gliadin and two γ-gliadin gene sequences were also incomplete^18^.

In this work, we achieved a genome-wide characterization of the gliadins accumulated in bread wheat grains by combining transcriptomic and proteomic approaches and with the aid of six newly prepared *Gli* locus deletion lines. Our transcriptomic analysis made use of the advantages of both second generation RNA sequencing (i.e., high throughput and accurate nucleotide sequence data) and third generation RNA sequencing (long read capacity and efficient construction of full-length transcripts) platforms. Furthermore, to maximize the chance to catch the whole set of gliadin transcripts, we conducted two separate PacBio RNA sequencing experiments using the RNAs extracted from pooled developing grains or those at 25 DAF in which the prolamin genes were strongly transcribed (Figures S1, S3, S5 and S6). This led to the identification of 52 unique full-length transcripts for four types of gliadins (i.e., α-, γ-, δ- and ω-gliadins), with 42 of them being active members. To our knowledge, this has not been achieved in previous studies on wheat gliadins.

Finding the protein products encoded by active gliadin genes from individual *Gli* loci has been a difficult task, because 1) multiple gliadin members from different loci are expressing in the same endospermic tissue, and 2) gliadins of the same type are often highly similar in both molecular mass and amino acid composition^17^,^18^,^39^,^40^,^41^,^42^,^43^. Here, this problem was dealt with by comparative proteomic analysis between Xiaoyan 81 and six *Gli* locus deletion mutants. In agreement with past studies^17^,^18^, we found that analysis of the 2-DE spots digested with chymotrypsin and thermolysin separately generated more informative peptides for gliadin identification. But unique from previous studies, we employed a set of deletion lines each lacking one of the six *Gli* loci, which permitted assignment of the identified gliadin spots to *Gli-A1, -B1, -D1, -A2, -B2*, or *-D2*. These *Gli* locus assigned 2-DE spots were then matched to 38 of the 42 active gliadin genes (full-length transcripts) revealed by PacBio RNA sequencing. And as such, the majority of the active gliadin genes were duly assigned to individual *Gli* loci. This assignment was verified to be correct by PCR mapping of 13 representative gliadin genes. The procedure outlined above was not successful for the four α-gliadin genes (*Gli-α7, Gli-α8, Gli-α18* and *Gliα-25*) without matching 2-DE spots. But with the availability of full-length transcript sequence information, the four genes were readily assigned to their hosting loci through PCR mapping.

The applications of full-length transcripts and *Gli* locus deletion lines are of central importance in promoting the efficient identification of gliadin genes expressed in our experimental variety. Apart from the active genes, we also identified full-length transcripts for 10 gliadin pseudogenes. Considering recent findings on the important roles of pseudogenes in regulating active gene expression^48^,^49^, the 10 pseudogenes may be valuable for future studies of the mechanisms controlling gliadin gene expression.

### A genome-wide view of the gliadins accumulated in bread wheat grains

Based on the data obtained in this work, we suggest that, during the grain development of Xiaoyan 81, 52 gliadin genes are transcribed, 42 of them carry intact coding sequence, and at least 38 of them have their proteins accumulated in the mature grains. The 38 expressed members include 21 α-, 11 γ-, 1 δ-, and 5 ω-gliadins. Clearly, the number of genes expressed is highest for α-gliadins, intermediate for γ-gliadins and relatively low for δ- and ω-gliadins. This is consistent with the finding that the content of α-gliadins in the flour is highest compared with that of γ- or ω-gliadins, and that the content of γ-gliadins is higher than that of ω-gliadins (Fig. 3). Although the transcript levels of the 11 γ-gliadin genes were generally higher than those of the 25 α-gliadin genes (Figures S1 and S3), this transcriptional difference was not sufficiently large to raise the content of γ-gliadins to that of α-gliadins. Clearly, the expression of more gene members is an important contributor to the very high accumulation level of α-gliadins in Xiaoyan 81 grains.

At the locus level, the number of gliadins accumulated in mature grains is four (*Gli-A1*), six (*Gli-B1*), seven (*Gli-D1*), six (*Gli-A2*), nine (*Gli-B2*) and six (*Gli-D2*), respectively. For the three subgenomes, the number of accumulated gliadins is highest for subgenome B (15), followed by subgenomes D (13) and A (10). Subgenome D expresses all four types of gliadins (α, γ, δ and ω), whereas subgenomes A and B express only α-, γ- and ω-gliadins. For the four α-gliadin genes (*Gli-α7, Gli-α8, Gli-α18* and *Gliα-25*) that did not have matching 2-DE spots, their protein products may not be present in the mature grains, or accumulated to a very low level if present. This is possible because the transcript levels of the four genes were generally and comparatively lower than other active α-gliadin genes (Figure S1).

Previously, Dupont and coauthors identified the presence of 23 α-gliadins, 13 γ-gliadins and seven ω-gliadins in the flour of the spring wheat variety Butte 86 by integrating EST analysis with proteomic approach^18^. However, their efforts to match gliadin genes with 2-DE spots might be complicated by the fact that many of the gliadin coding sequences constructed with ESTs were incomplete. Inclusion of the polypeptides deduced from incomplete coding sequences may compromise the matching of 2-DE spots to active gliadin genes because some of the partial coding sequences may be transcribed from gliadin pseudogenes. Many past studies have found the transcripts derived from gliadin pseudogenes^39^,^40^,^41^,^42^. According to our data, 19.2% of the gliadin full-length transcripts (i.e., 10 out of 52) were transcribed from pseudogenes. Consequently, in this work, we matched 2-DE spots to only the proteins deduced from 42 active gliadin genes, thus increasing the accuracy in matching active gliadin genes to their protein products.

It is interesting to note that α-gliadins can be divided into CT and CSTT groups (Figure S2). Remarkably, the α-gliadins specified by *Gli-B2* all belonged to the CSTT group, whereas the majority of the α-gliadins by *Gli-A2* or *-D2* belonged to the CT group. The CSTT α-gliadins are also present in other wheat genotypes and Triticeae species based on previous studies^25^,^39^,^50^,^51^. Consequently, further work is warranted to investigate the evolutionary and functional differences between CT and CSTT α-gliadins (see also below).

Similar to past studies^39^,^41^,^52^,^53^, this work identified one α- (Gli-α25) and two γ-gliadins (Gli-γ5 and Gli-γ10) carrying additional cysteine residue apart from the conserved ones, and two ω-gliadins (Gli-ω2 and Gli-ω5) each containing a cysteine residue (Table 1). Moreover, we found that Gli-γ5, Gli-γ10, Gli-ω2 and Gli-ω5 accumulated in the mature grains (Table 4), thus paving the way for their functional analysis in further research.

### Systematic mapping of CD epitopes in the gliadins accumulated in an elite bread wheat variety

Consistent with previous reports^5^,^10^,^24^,^25^,^26^,^27^, more numerous and diverse CD epitopes were detected in all γ-gliadins and many ω-gliadins, with the most celiac-toxic epitopes (DQ2.5-glia-α1a, DQ2.5-glia-α1b and DQ2.5-glia-α2) found in the majority of the α-gliadins specified by *Gli-D2* (Fig. 2 and Tables S4 and S5). The finding of less CD epitopes in the α-gliadins by *Gli-A2* was also in line with what was reported in the past^24^,^25^. However, we made two new observations. First, the 10 accumulated CSTT α-gliadins, including nine specified by *Gli-B2*, carried very few or no CD epitopes (Fig. 2 and Table S4). Second, the δ-gliadins identified in this work and by previous studies harbored no CD epitopes (Fig. 2, Table S5 and Figure S14). So for future development of the wheat lines beneficial to celiac patients, γ-gliadins, ω-gliadins and the CT group of α-gliadins (especially those by *Gli-D2*) should be removed (see also below). The CSTT α-gliadins and δ-gliadins may be kept to aid the likely function of gliadins in the end-use traits of wheat grains.

### Implications for future research

The data gathered in this work and the points discussed above have several implications for further research on wheat gliadins. First, the strategy for genome-wide characterization of gliadins detailed in this work should also be applicable for analyzing gliadin gene expression in other wheat genotypes. This is possible because PacBio transcriptome sequencing is becoming more and more effective^54^,^55^, and the method we used for isolating *Gli* locus deletion mutants is relatively easy to adopt.

Second, the 52 unique full-length transcripts and the 38 expressed gliadins identified by this work encompass the different types of gliadins known to exist in different wheat species and genotypes. They may be used as basic reference materials for future studies on (1) gliadin gene transcription and protein expression in different wheat materials, and (2) the roles of individual gliadins in CD and other gliadin related illnesses. As multiple wheat types are cultivated and consumed in different regions of the world^1^,^2^, and gliadin composition differs widely among wheat genotypes^7^,^8^, systematic studies of gliadin gene expression and functionalities will be important for continuously optimizing the health promotion effects of wheat grains. These studies will benefit from the sets of gliadin transcript and protein sequences reported by this work. Moreover, the six *Gli* locus deletion lines prepared by us may also aid studies on the minor gliadin loci (i.e., *Gli-A3, -A5, -A6, -B3, -B5, -D4* and *-D6*)^6^, for which there is still little structural and functional formation available.

Third, our insight on the distribution of CD epitopes in different gliadins and the significant reduction of celiac-toxic α-gliadins in the deletion line lacking *Gli-D2* may stimulate more efforts for developing the wheat lines with enhanced health benefits. A number of transgenic studies have shown that silencing gliadin gene expression may facilitate the development of wheat lines beneficial to the individuals affected by CD and other gliadin related illnesses^56^,^57^,^58^. An alternative, non-transgenic approach in this direction is to selectively remove the undesirable gliadin genes through mutagenesis. Our analysis reveals that many gliadins (i.e., 18 out of the 38 accumulated gliadins) do not carry, or contain very few, CD epitopes (Fig. 2 and Tables S4 and S5). Furthermore, the *Gli-D2* locus specifying the highly celiac-toxic gliadins can be removed without affecting the overall agronomic performance of bread wheat. Thus, the number of genes encoding harmful gliadin species is limited, and the development of non-transgenic wheat lines lacking undesirable gliadins but with improved health benefits is possible through mutagenesis and molecular breeding. In this context, the *Gli* locus deletion lines created by us may find useful applications because optimization of gliadin composition in bread wheat may best start from a genotype lacking one or more of the six *Gli* loci. However, to develop a wheat line completely free of CD epitopes is still difficult at present, because HMW-GSs and LMW-GSs, which are indispensable for wheat end-use quality control, also carry some CD epitopes^5^,^10^. In the long term, as gene editing technologies become more and more powerful^59^, it may be possible to eliminate CD epitopes from both gliadins and glutenins through genome engineering. Thus, the resources generated in this work may also help future genome editing studies of complex gliadins.

Finally, aside from eliciting CD, some gliadins also contribute to the occurrence of wheat allergy (WA)^10^. For example, ω5-gliadins have been found to be an important cause of the serious food allergy wheat-dependent excise-induced anaphylaxis^60^,^61^. There is also emerging evidence for the involvement of gliadins in a newly recognized gluten-dependent disorder termed nonceliac gluten sensitivity (NCGS) that affects 0.6 to 6% of the human population^10^,^62^. Consequently, the gliadin resources generated in this work may additionally help future studies on WA and NCGS related disorders.

In summary, this work shows that the combination of third generation of RNA sequencing, proteomic analysis and bioinformatic investigation is highly effective for resolving complex gliadins and derivative CD epitopes in bread wheat. The new insights and resources generated here may stimulate and facilitate more systematic studies of gliadin functions in CD and other wheat food related disorders and the development of healthier wheat in the future.

## Methods

### Plant materials

Xiaoyan 81 and the six *Gli* locus deletion lines were cultivated in the field^30^,^38^. At 25 DAF, the grains of Xiaoyan 81 were collected, and used for total RNA purification as described previously^38^. The mature grains of the seven lines were harvested, cleaned, and stored at 4 °C until use. The ion beam mutant population of Xiaoyan 81 used for isolating *Gli* locus deletion lines was reported in our previous study^30^. The six NT lines (N1AT1D, N1BT1D, N1DT1A, N6AT6D, N6BT6A and N6DT6B) and its WT progenitor CS were also described previously^63^,^64^.

### PacBio RNA sequencing and data analysis

We performed the third RNA sequencing experiment (RSE3) of Xiaoyan 81 developing grains collected at 25 DAF using PacBio RSII following the protocol detailed before^38^. In brief, three cDNA libraries (1–2 kb, 2–3 kb and >3 kb) were constructed and sequenced (Table S1). The subreads were processed, followed by FLNC reads identification and error correction using Illumina HiSeq transcriptomic reads generated in RSE2. Non-redundant transcripts were identified from the FLNC reads, and then mapped against the draft genome sequence of CS, with the corresponding loci divided into two types (extant and newly identified, Table S1). Finally, full-length transcripts of gluten genes in the FLNC reads were searched using local BlastN as reported previously^38^.

### Examining the transcriptional profile of gliadin genes

The transcriptional profile of the 42 active gliadin genes at 0, 10, 15 and 25 DAF was examined using the HiSeq transcriptomic data obtained in RSE2. HTSeq-count was employed to obtain the reads mapped to individual gliadin genes, with the transcript level expressed as reads per kilobase per million mapped reads (RPKM)^65^.

### Screening mutant population by MALDI-TOF-MS

Each gliadin extract was prepared using one half of the grain (lacking the embryo end). It was crushed into powder, followed by extraction in 1 ml of 70% ethanol for 40 min at room temperature (RT). The suspension was centrifuged (at 13,500 g) for 5 min at RT, and 200 μl supernatant was kept. The supernatant was centrifuged again for 5 min at RT, with 5 μl of the extract used for MALDI-TOF-MS analysis in an Autoflex MALDI-TOF mass spectrometer (Bruker Daltonics, Billerica, MA, USA). The processing of gliadin extract for MS analysis and calibration of mass spectra were conducted following the manufacturer’s instructions. The gliadin MS peaks of Xiaoyan 81 were compared to those of CS and six NT lines to infer their control by specific chromosomes (Figure S8). During the screening of M2 seeds, a positive sample (derived from the non-embryo half of a cut grain) was judged by missing one or more gliadin MS peaks. The corresponding embryo half of the positive grain was germinated and the resultant plant was raised to produce M3 seeds. The M3 seeds were again screened by MALDI-TOF-MS to validate inheritance of the desired mutation. Six non-redundant mutants, collectively lacking all 10 gliadin MS peaks of Xiaoyan 81, were used for developing the six *Gli* locus deletion lines (i.e., DLGliA1, DLGliB1, DLGliD1, DLGliA2, DLGliB2 and DLGliD1, Table 2). Three cycles of backcrossing (with Xiaoyan 81 as recurrent parent) were carried out to reduce background mutations. The *Gli* locus lacked in each of the six deletion lines was confirmed by examination using chromosome specific microsatellite markers (see below).

### Examination of six *Gli* locus deletion lines using microsatellite markers

Genomic DNA samples were prepared from the leaf tissues of Xiaoyan 81 and the six deletion lines, and used for microsatellite marker analysis following the methods described previously^64^. Because it is known that the six *Gli* loci are located on 1AS, 1BS, 1DS, 6AS, 6BS and 6DS, respectively^6^,^7^,^8^, we selected only the microsatellite markers located on these chromosomal arms for our analysis. Specifically, the microsatellite markers selected from the six arms were 5, 4, 6, 8, 7 and 6, respectively. The chromosomal positions of these markers and the nucleotide primers used for amplifying them are listed in Table S6.

### Proteomic analysis of gliadins

The gliadin samples prepared from the mature grains of Xiaoyan 81 and those of the six *Gli* locus deletion lines were comparatively analyzed in this series of experiments. The manipulations were mostly conducted at RT unless otherwise stated. Each gliadin sample was prepared using 5 half grains (embryo end removed), which were ground into a fine powder in liquid nitrogen. The powder was extracted 3 times with the RAG solution (0.4 M NaCl in 0.067 M KNaHPO_4_ buffer, pH 7.6) to remove albumins and globulins. For each extraction, 1 ml of RAG solution was used, the suspension was shaken for 10 min followed by centrifugation for 10 min at 13,500 g, and the supernatant was discarded. The pellet was then extracted twice using 70% ethanol to prepare gliadins. In each gliadin extraction, 500 μl of 70% ethanol was used, the suspension was agitated for 1 h followed by centrifugation for 10 min at 13,500 g, and the supernatant was kept. The combined supernatant was freeze-dried, with the resultant protein sample being redissolved in 250 μl lysis solution [7 M urea, 2 M thiourea, 40 mM DTT, 4% (w/v) CHAPS, and 2% (v/v) immobilized pH gradient buffer]. The protein solution was kept for 1 h with vortexing every 10 minutes, followed by centrifugation for 20 min at 15,800 g. The supernatant was transferred to a new tube, and its protein concentration was determined using the 2-D Quant Kit (GE Life Sciences, Beijing, China). Subsequently, the gliadin samples were subjected to 2-DE separation following the instructions detailed in the Handbook of 2-D Electrophoresis (Amersham Biosciences UK Ltd, Buckinghamshire, UK). The first dimension was conducted using Immobiline DryStrip gels (IPG strips) with a linear pH gradient of 3–10 (24 cm) or 6–11 (18 cm). The second dimension was accomplished in 12% SDS-PAGE. For each gliadin sample, at least 5 different 2-DE separations were executed, with the amount of gliadins used varying from 150–350 μg in order to maximize the resolution of individual protein spots.

The protein spots were excised from 2-DE gels, and digested by chymotrypsin or thermolysin following the methods detailed previously^17^,^18^. MS/MS analysis of the digests was conducted on a nano LC-LTQ-MS/MS platform (Thermo Scientific, Waltham, MA). The resulting MS/MS data were analyzed in two ways. First, for investigating the protein identity of each excised spot, a Wheat_Gluten database was constructed with the protein sequences obtained by searching NCBInr database using the key words ‘prolamin’, ‘gliadin’, ‘gluten’, ‘glutenin’, ‘storage’, ‘avenin’, ‘hordein’ and ‘secalin’ according to a previous study^66^. Redundant sequences in the resultant database were removed using the program Cd-hit^67^. The MS spectra obtained in this work were interrogated against the Wheat_Gluten database using both MASCOT and SEQUEST search engines in the Proteome Discoverer software (Version 1.4, Thermo Scientific, Bremen, Germany). Carbamidomethylation of cysteine and oxidation of methionine were used as variable residue modifications. The following parameters were used during the search. MS/MS extraction: 300–6000 Da; Peak count > 3; Intensity > 50; Maximum missed cleavage: 2; q value < 0.01; Precursor mass tolerance: 3 Da; Fragment mass tolerance: 0.8 Da. This analysis identified the excised 2-DE spots as different types of gliadins or LMW-GSs. Second, for finding correspondence between the active gliadin genes of Xiaoyan 81 and the 2-DE gliadin spots, another database (Xiaoyan 81_Gluten) was assembled by combining the 58 gluten proteins of Xiaoyan 81 and the common contaminant proteins provided by the software MaxQuant documentation (http://www.coxdocs.org/doku.php?id=: maxquant:start). The 58 proteins of Xiaoyan 81 were deduced from the active gluten gene transcripts (5 coding for HMW-GSs, 11 for LMW-GSs and 42 for gliadins) uncovered by our previous study^38^. The MS spectra of the gliadin spots were searched against Xiaoyan 81_Gluten database as described above. Positive correspondence was based on the presence of unique peptide as well as coverage of the target protein by the MS peptides.

### PCR mapping of gliadin genes

Gene specific primers (listed in Table S7) were designed for each of the 17 gliadin genes subjected to PCR mapping. The DNA templates used for the mapping experiment were prepared from Xiaoyan 81 and the six *Gli* locus deletion lines. The method for extracting genomic DNA and the conditions of PCR were described previously^63^,^64^.

### Bioinformatic analysis of CD epitopes

The deduced proteins of the 38 gliadins accumulated in Xiaoyan 81 mature grains were each examined for the presence of the 24 CD epitopes compiled for gliadins^5^. The examination was carried out using the “search for sequence” function of the DNAMAN software (Lynnon Biosoft, Lynnon Corporation, http://www.lynnon.com).

### Measuring gliadin and CD epitope levels in Xiaoyan 81 and DLGliD2

The grains of Xiaoyan 81 and DLGliD2, harvested from the experimental farm in 2014 and 2015, respectively, were milled, with the resulting flour samples used for assaying the gliadin and CD epitope levels. The assessment of gliadins was accomplished with reverse phase high performance liquid chromatography (RP-HPLC) following the method described in our previous work^30^. The levels of CD epitopes in the flour samples were determined using an AgraQuant^®^ Gluten G12 Assay kit (Romer Labs UK Ltd., Cheshire, UK), with some modifications in preparing the gluten sample. Briefly, each gluten sample was prepared by suspending 100 mg flour in 2.5 ml extraction buffer (supplied with the kit). The suspension was shaken vigorously, followed by incubation at 50 °C for 40 minutes in a laboratory vortex. After the incubation, the suspension was cooled to RT, and 7.5 ml of 80% ethanol was added. The suspension was again shaken for 60 minutes at RT with a laboratory rotator. Afterwards, a centrifugation (5 min, 12,000 g) was applied, and the supernatant was kept. The supernatant was diluted with the sample dilution buffer supplied in the kit before being used in the assay. The remaining steps of the assay were finished following the instructions of the supplier. In these experiments, three separate samples were analyzed for both Xiaoyan 81 and DLGliD2 and for each year, with the resultant data expressed as means ± SD. Statistical analysis of the data was accomplished by one-way ANOVA (with genotype as factor and Bonferroni correction) or independent samples t-test in the SPSS program (SPSS Inc., Chicago, IL, USA).

### Data availability

The 197,709 FLNC reads produced in this study have been deposited in the BioProject database of National Center for Biotechnology Information (accession number SRR4279819).

## Additional Information

**How to cite this article:** Wang, D.-W. *et al*. Genome-wide analysis of complex wheat gliadins, the dominant carriers of celiac disease epitopes. *Sci. Rep.* **7**, 44609; doi: 10.1038/srep44609 (2017).

**Publisher's note:** Springer Nature remains neutral with regard to jurisdictional claims in published maps and institutional affiliations.

## Supplementary Material

Supplementary Information

Supplementary Table S3

## Figures and Tables

**Table 1:** List of 42 unique and active full-length transcripts found for different types of gliadins through RNA sequencing.

α-gliadin				γ-, δ- and ω-gliadin			
Full-length transcript^a^	Cognate gene	Protein (aa)^b^	Chromosomal location^c^	Full-length transcript^a^	Cognate gene	Protein (aa)^b^	Chromosomal location^c^
α1 (31)	*Gli-α1*	293	6A	γ1 (38)	*Gli-γ1*	339	1A
α2 (45)	*Gli-α2*	293	6A	γ2 (28)	*Gli-γ2*	285	1A
α3 (69)	*Gli-α3*	287	6A	γ3 (19)	*Gli-γ3*	285	1A
α4 (62)	*Gli-α4*	287	6A	γ4 (67)	*Gli-γ4*	357	1B
α5 (24)	*Gli-α5*	286	6A	γ5 (14)	*Gli-γ5*	**302 (9 cys)**	1B
α6 (32)	*Gli-α6*	289	6A	γ6 (16)	*Gli-γ6*	297	1B
α7 (10)	*Gli-α7*	287	6A	γ7 (72)	*Gli-γ7*	291	1B
α8 (7)	*Gli-α8*	296	6A	γ8 (83)	*Gli-γ8*	327	1D
α9 (10)	*Gli-α9*	325	6B	γ9 (60)	*Gli-γ9*	298	1D
α10 (75)	*Gli-α10*	299	6B	γ10 (36)	*Gli-γ10*	**295 (9 cys)**	1D
α11 (19)	*Gli-α11*	293	6B	γ11 (86)	*Gli-γ11*	298	1D
α12 (23)	*Gli-α12*	293	6B				
α13 (17)	*Gli-α13*	296	6B	δ1 (4)	*Gli-δ1*	327	1D
α14 (9)	*Gli-α14*	296	6B				
α15 (84)	*Gli-α15*	313	6B	ω1 (10)	*Gli-ω1*	359	1A
α16 (83)	*Gli-α16*	313	6B	ω2 (12)	*Gli-ω2*	**406 (1 cys)**	1B
α17 (58)	*Gli-α17*	297	6B	ω3 (18)	*Gli-ω3*	439	1B
α18 (2)	*Gli-α18*	312	6B	ω4 (9)	*Gli-ω4*	387	1D
α19 (24)	*Gli-α19*	282	6D	ω5 (7)	*Gli-ω5*	**377 (1 cys)**	1D
α20 (15)	*Gli-α20*	286	6D				
α21 (57)	*Gli-α21*	293	6D				
α22 (54)	*Gli-α22*	308	6D				
α23 (44)	*Gli-α23*	309	6D				
α24 (6)	*Gli-α24*	291	6D				
α25 (9)	*Gli-α25*	**299 (7 cys)**	6D				

^a^The value in brackets indicates the total number of FLNC reads found in RSE1 and RSE3 for the given full-length transcript.

^b^Deduced from the listed full-length transcripts. The gliadin having variant number of conserved cysteine (cys) residues is shown in bold.

^c^Chromosomal location of the gene yielding the given transcript.

**Table 2:** Characteristics of the six deletion lines lacking individual *Gli* loci.

Deletion line	MALDI-TOF-MS peak missed^a^	*Gli* locus deleted	Size of the deletion (cM)^b^	Location of the deletion	Diagnostic microsatellite marker (missed in the deletion line)
DLGliA1	9^#^	*Gli-A1*	>40.4	1AS	*Xgdm33, Xcfa2153, Xgpw7072, Xgpw2276*, and *Xwmc24*
DLGliB1	10^#^	*Gli-B1*	>23.2	1BS	*Xpsp3000, Xwmc406, Xwmc230*, and *Xgpw4069*
DLGliD1	4^#^, 8^#^	*Gli-D1*	>12.5	1DS	*Xwmc147, Xgpw7082, Xwmc432, Xcfd15*, and *Xwmc336*
DLGliA2	2^#^	*Gli-A2*	>51.5	6AS	*Xgwm334, Xgpw2082, Xgpw7073, Xgpw7076*, and *Xgpw7592*
DLGliB2	5^#^, 7^#^	*Gli-B2*	>7	6BS	*Xgdm113, Xbarc14, Xpsp3009, Xwmc494*, and *Xgwm508*
DLGliD2	1^#^, 3^#^, 6^#^	*Gli-D2*	>23.5	6DS	*Xcfd132, Xcfd33*, and *Xgdm127*

^a^The ten compound peaks are shown in Figure S8a.

^b^Detailed in Figure S9.

**Table 3:** The 2-DE gliadin spots lacked in individual *Gli* locus deletion lines.

Deletion line (*Gli* locus deleted)	Gliadin spot lacked		*Gli* locus assignment of the spot
	2-DE spot^a^	Total	
DLGliA1 (*Gli-A1*)	1–10	10	*Gli-A1*
DLGliB1 (*Gli-B1*)	11–27	17	*Gli-B1*
DLGliD1 (*Gli-D1*)	29–48	20	*Gli-D1*
DLGliA2 (*Gli-A2*)	49–63	15	*Gli-A2*
DLGliB2 (*Gli-B2*)	64–76	13	*Gli-B2*
DLGliD2 (*Gli-D2*)	77–83	7	*Gli-D2*

^a^Spots 28 and 84 were not included in the table because both were mixtures of different types of gliadins.

**Table 4:** Matching between active gliadin genes and 2-DE spots and *Gli* locus assignment of the active genes.

α-gliadin			γ-, δ-, and ω-gliadin		
Active gene	2-DE spot	Assignment to *Gli* locus^b^	Active gene	2-DE spot	Assignment to *Gli* locus^b^
*Gli-α1*	50, 51	*Gli-A2* (SA + PM)	*Gli-γ1*	1–3	*Gli-A1* (SA + PM)
*Gli-α2*	50, 51	*Gli-A2* (SA)	*Gli-γ2*	4–9	*Gli-A1* (SA)
*Gli-α3*	61–63	*Gli-A2* (SA)	*Gli-γ3*	4–9	*Gli-A1* (SA)
*Gli-α4*	52, 57	*Gli-A2* (SA + PM)	*Gli-γ4*	12–16, 22, 23	*Gli-B1* (SA)
*Gli-α5*	54–56, 60	*Gli-A2* (SA + PM)	*Gli-γ5*	17–20	*Gli-B1* (SA)
*Gli-α6*	49, 53, 58, 59	*Gli-A2* (SA)	*Gli-γ6*	21	*Gli-B1* (SA)
*Gli-α7*	NMF^a^	*Gli-A2* (PM)	*Gli-γ7*	24–26	*Gli-B1* (SA)
*Gli-α8*	NMF	*Gli-A2* (PM)	*Gli-γ8*	30–33	*Gli-D1* (SA)
*Gli-α9*	64	*Gli-B2* (SA + PM)	*Gli-γ9*	34–37	*Gli-D1* (SA)
*Gli-α10*	65, 76	*Gli-B2* (SA + PM)	*Gli-γ10*	38–39	*Gli-D1* (SA)
*Gli-α11*	66	*Gli-B2* (SA + PM)	*Gli-γ11*	40–42	*Gli-D1* (SA + PM)
*Gli-α12*	66	*Gli-B2* (SA)			
*Gli-α13*	67	*Gli-B2* (SA)	*Gli-δ1*	29	*Gli-D1* (SA)
*Gli-α14*	73	*Gli-B2* (SA)			
*Gli-α15*	68, 71, 72	*Gli-B2* (SA)	*Gli-ω1*	10	*Gli-A1* (SA)
*Gli-α16*	70	*Gli-B2* (SA + PM)	*Gli-ω2*	27	*Gli-B1* (SA + PM)
*Gli-α17*	69, 74, 75	*Gli-B2* (SA + PM)	*Gli-ω3*	11	*Gli-B1* (SA)
*Gli-α18*	NMF	*Gli-B2* (PM)	*Gli-ω4*	43–48	*Gli-D1* (SA)
*Gli-α19*	77	*Gli-D2* (SA)	*Gli-ω5*	43, 44	*Gli-D1* (PM)
*Gli-α20*	83	*Gli-D2* (SA + PM)			
*Gli-α21*	80	*Gli-D2* (SA)			
*Gli-α22*	78	*Gli-D2* (SA)			
*Gli-α23*	79	*Gli-D2* (SA)			
*Gli-α24*	81, 82	*Gli-D2* (SA + PM)			
*Gli-α25*	NMF	*Gli-D2* (PM)			

^a^NMF, no match found.

^b^Based on 2-DE spot assignment (SA) (Table 3), PCR mapping (PM) or both (SA + PM).
