# Supplementary material for: Genome-wide analysis of complex wheat gliadins, the dominant carriers of celiac disease epitopes
Source: Sci Rep. 2017 Mar 16;7:44609. doi: 10.1038/srep44609 (PMC5353739; doi:10.1038/srep44609)
Supplement: Supplementary Information [file srep44609-s1.pdf]

Supplementary Information

Genome-wide analysis of complex wheat gliadins, the dominant carriers of celiac disease epitopes

Da-Wei Wang[1,2,3,+], Da Li[1,2,+], Junjun Wang[1,2,+], Yue Zhao[1], Zhaojun Wang[1,2], Guidong Yue[1], Xin Liu[1], Huanju Qin[1], Kunpu Zhang[1], Lingli Dong[1] & Daowen Wang[1,2,4]

[1]The State Key Laboratory of Plant cell and Chromosome Engineering, Institute of Genetics and Developmental Biology, Chinese Academy of Sciences, Beijing, China. [2]University of Chinese Academy of Sciences, Beijing, China. [3]Tobacco Research Institute, Chinese Academy of Agricultural Sciences, Qingdao, China. [4]The Collaborative Innovation Center for Grain Crops, Henan Agricultural University, Zhengzhou, China.

[+]These authors contributed equally to this work.

Corresponding authors: Kunpu Zhang (kpzhang@genetics.ac.cn), Lingli Dong (lldong@genetics.ac.cn) & Daowen Wang (dwwang@genetics.ac.cn).

Figure S1. Transcript levels of 25 α-gliadin genes (*Gli-α1* to *-α25*) in the developing grains of Xiaoyan 81 at 0, 10, 15 and 25 days after flowering (DAF). The 25 genes came from the A, B or D genome of common wheat. The transcript level was expressed as reads per kilobase per million mapped reads (RPKM).

Figure S2. Amino acid sequence comparison of 25 α-gliadins (Gli-α1 to -α25). The deduced protein sequences were aligned using Clustal Omega (http://www.ebi.ac.uk/Tools/msa/clustalo/) with manual adjustment. The 25 α-gliadins shared a common primary structure composed of a signal peptide (SP), a N-terminal repetitive region (NRR), two poly-glutamine regions (PQR1 and PQR2), and two unique regions (UR1 and UR2). The six conserved cysteine residues are marked in red. The additional cysteine found in Gli-α25 is boxed in purple. The CSTT element shared by Gli-α8 to -α19 is shaded in yellow. The amino acid substitutions, which are unique to CSTT α-gliadins and located in PQR1 and UR1, respectively, are also shaded in yellow.

Figure S3. Transcript levels of 11 γ-gliadin genes (*Gli-γ1* to *-γ11*) in the developing grains of Xiaoyan 81 at 0, 10, 15 and 25 days after flowering (DAF). The 11 genes resided in the A, B or D genome of common wheat. The transcript level was expressed as reads per kilobase per million mapped reads (RPKM).

Figure S4. Amino acid sequence comparison of 11 γ-gliadins (Gli-γ1 to -γ11). The protein sequences were aligned using Clustal Omega with manual adjustment. The 11 γ-gliadins possessed a common primary structure consisted of a signal peptide (SP), a N-terminal region (NR), a repetitive region (RR), two unique regions (UR1 and UR2), and a poly-glutamine region (PQR). These five regions have also been designated as I (NR), II (RR), III (UR1), IV (PQR) and V (UR2) by past studies[40,41]. The eight conserved cysteine residues are marked in red. The extra cysteine found in the repetitive region of Gli-γ5 or Gli-γ10 is boxed in purple.

Figure S5. Analysis of the transcript level of *Gli-δ1* and its deduced protein. **a** Transcript level of *Gli-δ1*, expressed as reads per kilobase per million mapped reads (RPKM), in the developing grains of Xiaoyan 81 at 0, 10, 15 and 25 days after flowering (DAF). This gene was located in the D genome of common wheat. **b** comparison of Gli-δ1 with two representative homologous proteins (Gli-δ-CS1B and Gli-δ-CS1D) from the common wheat variety Chinese Spring (CS). The three proteins

shared a common primary structure composed of a signal peptide (SP), a N-terminal region (NR), a repetitive region (RR), and a C-terminal region (CR). The eight conserved cysteines are labelled in blue. Gli-δ-CS1B and Gli-δ-CS1D were characterized by two previous studies[28,29].

Figure S6. Transcript levels of five ω-gliadin genes (*Gli-ω1* to *-ω5*) in the developing grains of Xiaoyan 81 at 0, 10, 15 and 25 days after flowering (DAF). The transcript level was expressed as reads per kilobase per million mapped reads (RPKM). The five genes resided in the A, B or D genome of common wheat.

Figure S7. Analysis of the five deduced ω-gliadins (Gli-ω1 to -ω5) of Xiaoyan 81. **a** Comparison of Gli-ω1, -ω4 and -ω5 to two previously published homologous ω-gliadins with the N-terminus starting by AREL or ARQL (GenBank accessions AAT74547 and CAR82268, respectively). **b** Comparison of Gli-ω3 to a representative homologous ω-gliadin with the N-terminus starting by SRLL (GenBank accession BAE20328). **c** Comparison of Gli-ω2 to a previously reported D-type glutenin protein (GenBank accession AJ937839). In the above comparisons, the putative signal peptide is marked in blue, and the first four residues in the N-terminus (AREE, ARQL SRLL or ARPL) are written in brown. The cysteine residue found in Gli-ω2 or Gli-ω5 is boxed in purple. Asterisks indicate identical residues, whereas colons and periods represent conserved and semi-conserved substitutions, respectively.

Figure S8. Examination of the MALDI-TOF-MS peaks of Xiaoyan 81 gliadin extract. **a** The 10 compound MALDI-TOF-MS peaks of Xiaoyan 81 gliadin extract in the mass range of 25 - 45 kD. **b** Overlapping between the main MALDI-TOF-MS peaks of the gliadin extracts of Xiaoyan 81 (blue) and Chinese Spring (red). **c** Chromosomal control of the 10 main gliadin MS peaks as investigated using Chinese Spring (CS) and derivative nulli-tetrasomic (NT) lines lacking chromosome 1A (N1AT1D), 1B (N1BT1D), 1D (N1DT1A), 6A (N6AT6D), 6B (N6BT6A) or 6D (N6DT6B). The gliadin MS peak(s) missed in each of the six NT lines is indicated by red arrow(s).

Figure S9. Analysis of the six *Gli* locus deletion lines of Xiaoyan 81 using microsatellite markers. The deletion of the chromosomal fragment carrying *Gli-A1*, *-B1*, *-D1*, *-A2*, *-B2* or *-D2* in the six lines (DLGliA1, DLGliB1, DLGliD1, DLGliA2, DLGliB2 and DLGliD2) was investigated using chromosome specific microsatellite

markers. The total number of markers used was five (1AS), four (1BS), six (1DS), eight (6AS), seven (6BS) or six (6DS). The markers missed in the deletion lines are indicated by red arrows, whereas those still detectable are labeled by green arrows. The unlabeled markers were not examined. The genetic positions of the markers (cM) are provided on the left side of each graph. The linkage data of the markers are from GrainGenes (http://wheat.pw.usda.gov/ggpages/SSRclub/GeneticPhysical/).

Figure S10. Grains harvested from Xiaoyan 81 and the six *Gli* locus deletion lines. Xiaoyan 81 and the *Gli* locus deletion lines (DLGliA1, DLGliB1, DLGliD1, DLGliA2, DLGliB2 and DLGliD2) were cultivated in the field. The grains shown are typical of the harvests from four field environments conducted in 2013 to 2016.

Figure S11. A typical 2-DE gel image of Xiaoyan 81 gliadin extract obtained by using the IPG strip with a linear pH gradient of 6-11 in the first dimension. The second dimension was run in 12% SDS-PAGE. The majority of the gliadin spots were well separated (see also insets). The spots in the two dash-line boxes were found to be LMW-GSs by subsequent MS/MS analysis. The size (kD) of the protein markers is indicated on the left side of the graph.

Figure S12. A representative 2-DE gel graph of Xiaoyan 81 gliadin extract generated by using the IPG strip with a linear pH gradient of 3-10 in the first dimension. The second dimension was conducted in 12% SDS-PAGE. The gliadin spots in the two boxes were better resolved than using the IPG strip with a linear pH gradient of 6-11 in the first dimension. The size (kD) of the protein markers is shown on the left side of the image.

Figure S13. A typical result of validating the location of gliadin genes in specific *Gli* loci by PCR mapping. The validation was carried out with gene specific primers and the genomic DNA samples of Xiaoyan 81 and the six *Gli* locus deletion lines (DLGliA1, DLGliB1, DLGliD1, DLGliA2, DLGliB2 and DLGliD2) lacking *Gli-A1*, *Gli-B1*, *Gli-D1*, *Gli-A2*, *Gli-B2* and *Gli-D2*, respectively. The gliadin genes validated were *Gli-γ1*, *Gli-ω2*, *Gli-α9* and *Gli-α20*. The lengths of the PCR amplicons for the four genes are indicated in the brackets. The size (bp) of the DNA markers is shown on the left side of the image.

Figure S14. Absence of CD epitopes in previously reported δ-gliadins. The five

δ-gliadins were identified in Chinese Spring (Gli-δ-CS1B and Gli-δ-CS1D), Hereward (Gli-δ-He1) or *Aegilops tauschii* (Gli-δ-Aet1 and Gli-δ-Aet2) by two previous studies[28,29]. The GenBank accession numbers for Gli-δ-He1, Gli-δ-Aet1 and Gli-δ-Aet2 are HE819390, JX081265 and JX295577, respectively.

Table S1. Summary of the data of RNA sequencing experiment 3 (RSE3).

Table S2. Sequence information of the 52 gliadin genes transcribed in the grains of Xiaoyan 81.

Table S3: Analysis of mass spectrometry data obtained for the 82 gliadin spots of Xiaoyan 81 separated by 2-DE.

Table S4. Computation of coeliac disease (CD) epitopes in the 21 α-gliadins accumulated in Xiaoyan 81 mature grains.

Table S5. Computation of coeliac disease (CD) epitopes in the 11 γ-gliadin, 1 δ-gliadin and 5 ω-gliadin proteins accumulated in Xiaoyan 81 mature grains.

Table S6. PCR primers used for microsatellite marker analysis of the six *Gli* locus deletion lines.

Table S7. PCR primers used for mapping the chromosomal locations of 17 gliadin genes.

Figure S1

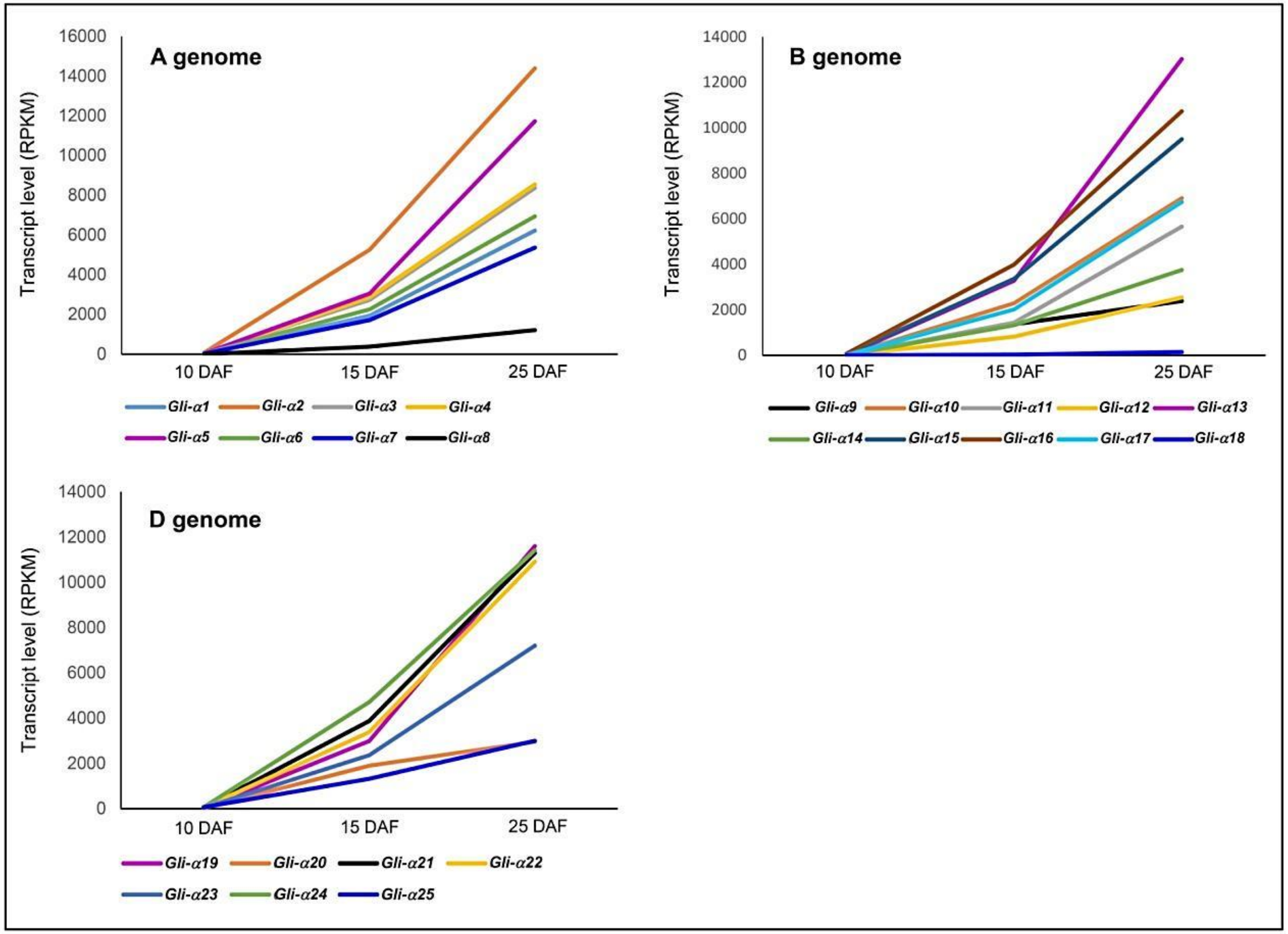

A genome
Transcript level (RPKM)
16000
14000
12000
10000
8000
6000
4000
2000
0
10 DAF
15 DAF
25 DAF
Gli-α1
Gli-α2
Gli-α3
Gli-α4
Gli-α5
Gli-α6
Gli-α7
Gli-α8
B genome
Transcript level (RPKM)
14000
12000
10000
8000
6000
4000
2000
0
10 DAF
15 DAF
25 DAF
Gli-α9
Gli-α10
Gli-α11
Gli-α12
Gli-α13
Gli-α14
Gli-α15
Gli-α16
Gli-α17
Gli-α18
D genome
Transcript level (RPKM)
14000
12000
10000
8000
6000
4000
2000
0
10 DAF
15 DAF
25 DAF
Gli-α19
Gli-α20
Gli-α21
Gli-α22
Gli-α23
Gli-α24
Gli-α25

Figure S2

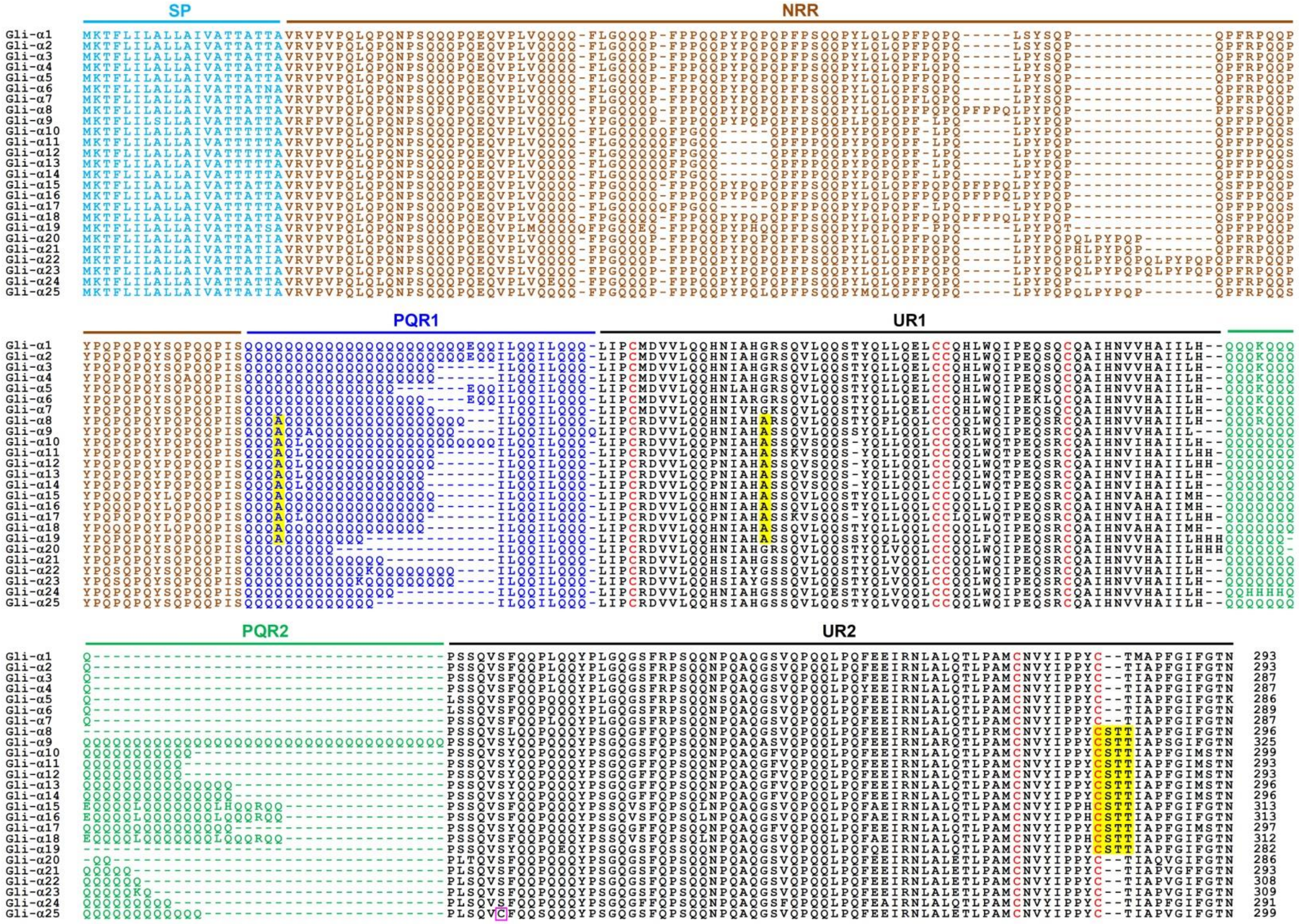

Figure S3

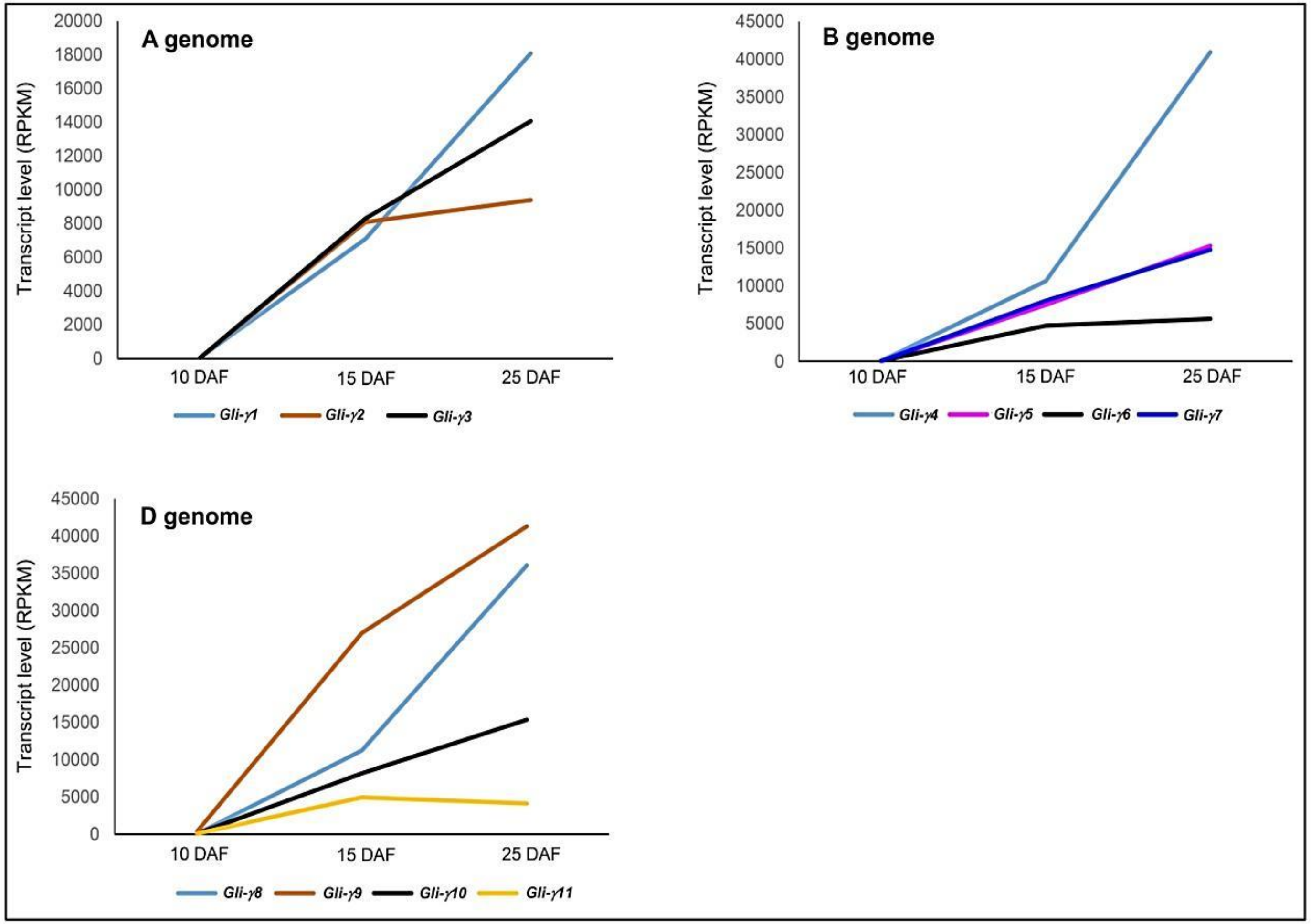
A genome
Transcript level (RPKM)
20000
18000
16000
14000
12000
10000
8000
6000
4000
2000
0
10 DAF
15 DAF
25 DAF
Gli-γ1
Gli-γ2
Gli-γ3
B genome
Transcript level (RPKM)
45000
40000
35000
30000
25000
20000
15000
10000
5000
0
10 DAF
15 DAF
25 DAF
Gli-γ4
Gli-γ5
Gli-γ6
Gli-γ7
D genome
Transcript level (RPKM)
45000
40000
35000
30000
25000
20000
15000
10000
5000
0
10 DAF
15 DAF
25 DAF
Gli-γ8
Gli-γ9
Gli-γ10
Gli-γ11

Figure S4

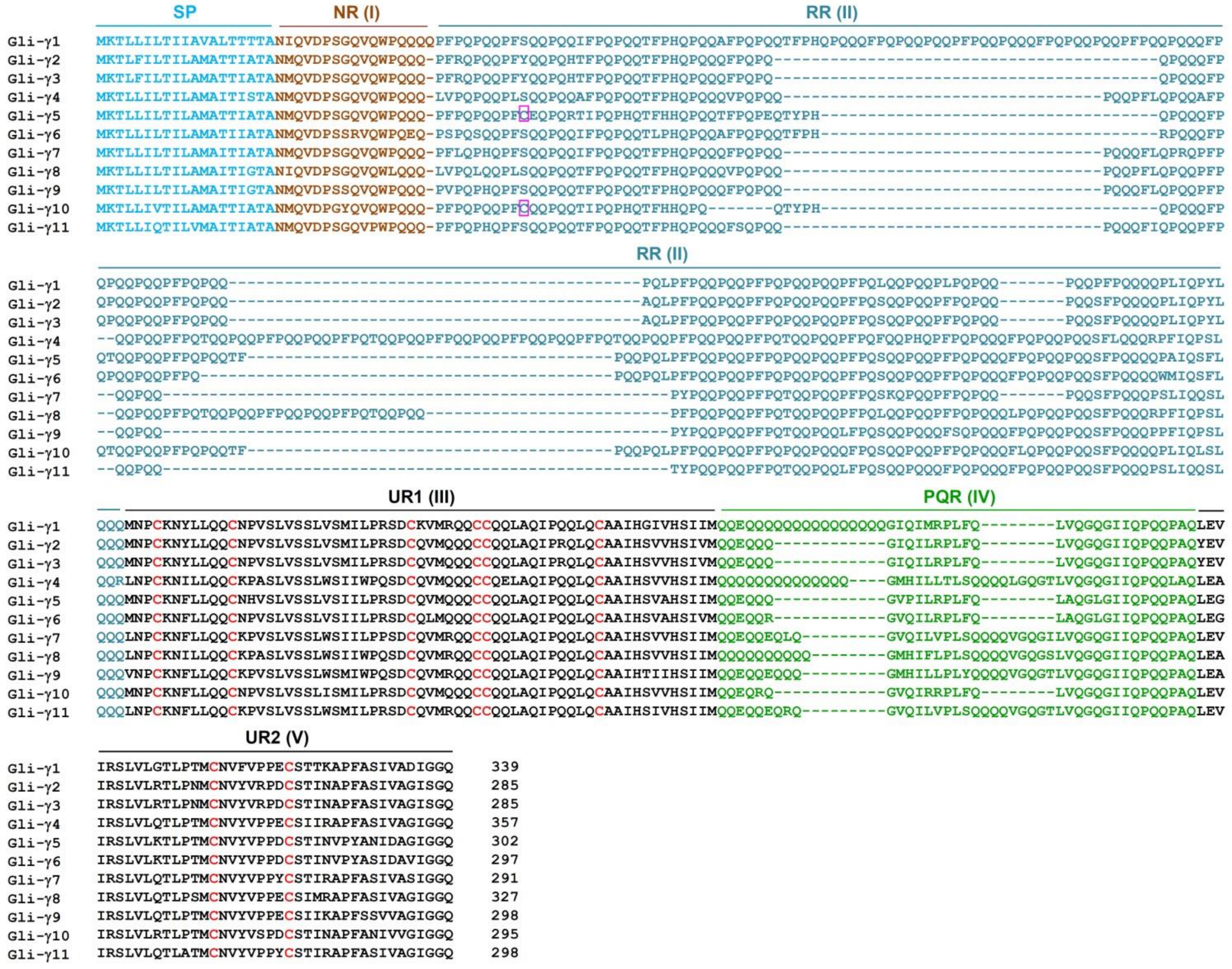
SP
NR (I)
RR (II)
RR (II)
UR1 (III)
PQR (IV)
UR2 (V)
Gli-γ1 IRSLVLGTLPTMCNVFVPPECSTTKAPFASIVADIGGQ 339
Gli-γ2 IRSLVLRTLPNMCNVYVRPDCSTINAPFASIVAGISGQ 285
Gli-γ3 IRSLVLRTLPNMCNVYVRPDCSTINAPFASIVAGISGQ 285
Gli-γ4 IRSLVLQTLPTMCNVYVPPECSIIRAPFASIVAGIGGQ 357
Gli-γ5 IRSLVLKTLPTMCNVYVPPDCSTINVPYANIDAGIGGQ 302
Gli-γ6 IRSLVLKTLPTMCNVYVPPDCSTINVPYASIDAVIGGQ 297
Gli-γ7 IRSLVLQTLPTMCNVYVPPYCSTIRAPFASIVASIGGQ 291
Gli-γ8 IRSLVLQTLPSMCNVYVPPECSIMRAPFASIVAGIGGQ 327
Gli-γ9 IRSLVLQTLPTMCNVYVPPECSIIKAPFSSVVAGIGGQ 298
Gli-γ10 IRSLVLRTLPTMCNVYVSPDCSTINAPFANIVVGIGGQ 295
Gli-γ11 IRSLVLQTLATMCNVYVPPYCSTIRAPFASIVAGIGGQ 298

Figure S5

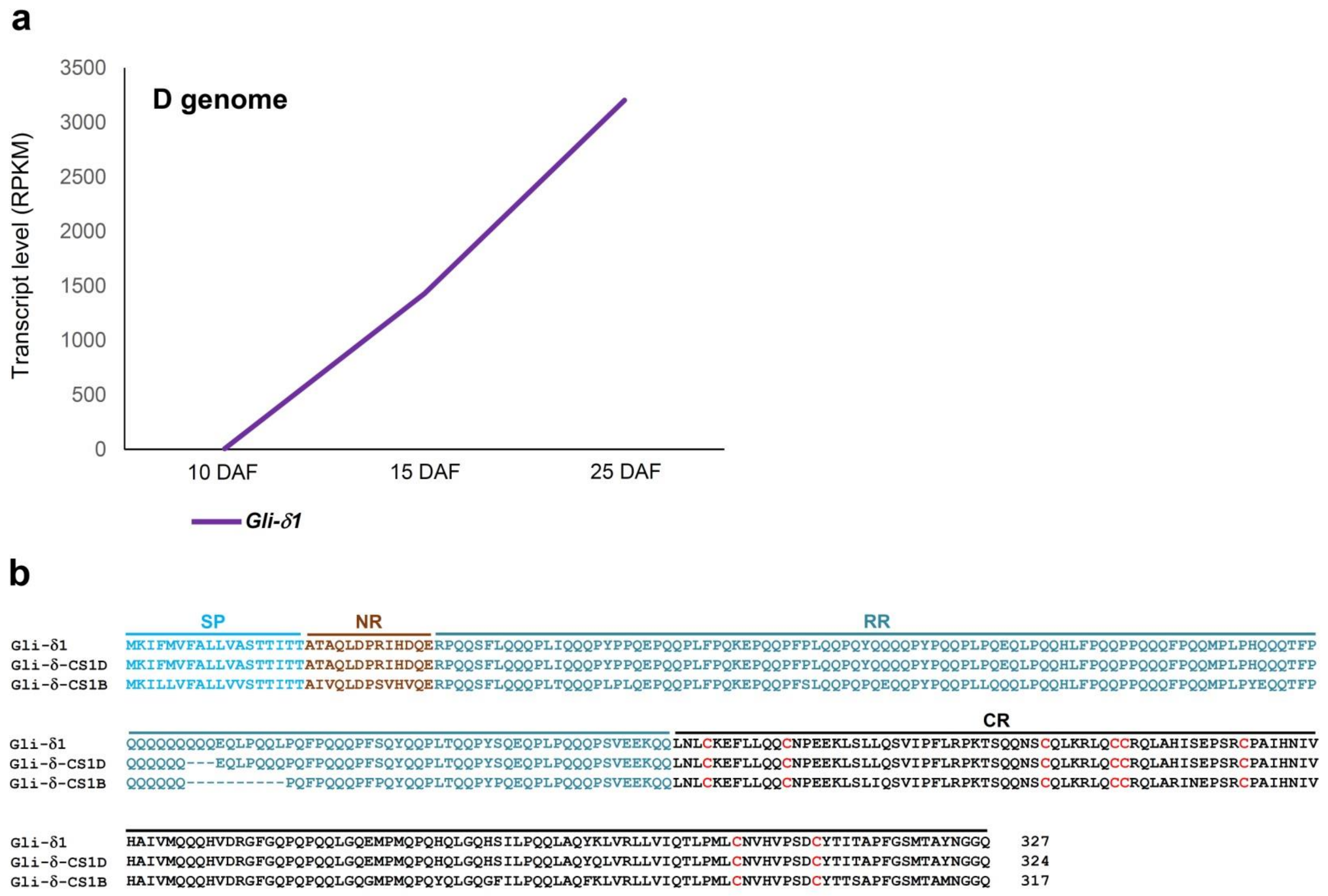

a
b
3500
3000
2500
2000
1500
1000
500
0
Transcript level (RPKM)
D genome
10 DAF
15 DAF
25 DAF
Gli-δ1
SP
NR
RR
Gli-δ1 MKIFMVFALLVASTTITTATAQLDPRIHDQERPQQSFLQQQPLIQQQPYPPQEPQQPLFPQKEPQQPFPLQQPQYQQQQPYPQQPLPQEQLPQQHLFPQQPPQQQFPQQMPLPHQQQTFP
Gli-δ-CS1D MKIFMVFALLVASTTITTATAQLDPRIHDQERPQQSFLQQQPLIQQQPYPPQEPQQPLFPQKEPQQPFPLQQPQYQQQQPYPQQPLPQEQLPQQHLFPQQPPQQQFPQQMPLPHQQQTFP
Gli-δ-CS1B MKILLVFALLVVSTTITTAIVQLDPSVHVQERPQQSFLQQQPLTQQQPLPLQEPQQPLFPQKEPQQPFSLQQPQPQEQQPYPQQPLLQQQLPQQHLFPQQPPQQQFPQQMPLPYEQQTFP
CR
Gli-δ1 QQQQQQQQQEQLPQQLPQFPQQQPFSQYQQPLTQQPYSQEQPLPQQQPSVEEKQQLNLCKEFLLQQCNPEEKLSLLQSVIPFLRPKTSQQNSCQLKRLQCCRQLAHISEPSRCPAIHNIV
Gli-δ-CS1D QQQQQQ---EQLPQQQPQFPQQQPFSQYQQPLTQQPYSQEQPLPQQQPSVEEKQQLNLCKEFLLQQCNPEEKLSLLQSVIPFLRPKTSQQNSCQLKRLQCCRQLAHISEPSRCPAIHNIV
Gli-δ-CS1B QQQQQQ----------PQFPQQQPFPQYQQPLTQQPYPQEQPLPQQQPSVEEKQQLNLCKEFLLQQCNPEEKLSLIQSVIPFLRPKTSQQNSCQLKRLQCCRQLARINEPSRCPAIHNIV
Gli-δ1 HAIVMQQQHVDRGFGQPQPQQLGQEMPMQPQHQLGQHSILPQQLAQYKLVRLLVIQTLPMLCNVHVPSDCYTITAPFGSMTAYNGGQ 327
Gli-δ-CS1D HAIVMQQQHVDRGFGQPQPQQLGQEMPMQPQHQLGQHSILPQQLAQYQLVRLLVIQTLPMLCNVHVPSDCYTITAPFGSMTAYNGGQ 324
Gli-δ-CS1B HAIVMQQQHVDRGFGQPQPQQLGQGMPMQPQYQLGQGFILPQQLAQFKLVRLLVIQTLPMLCNVHVPSDCYTTSAPFGSMTAMNGGQ 317

Figure S6

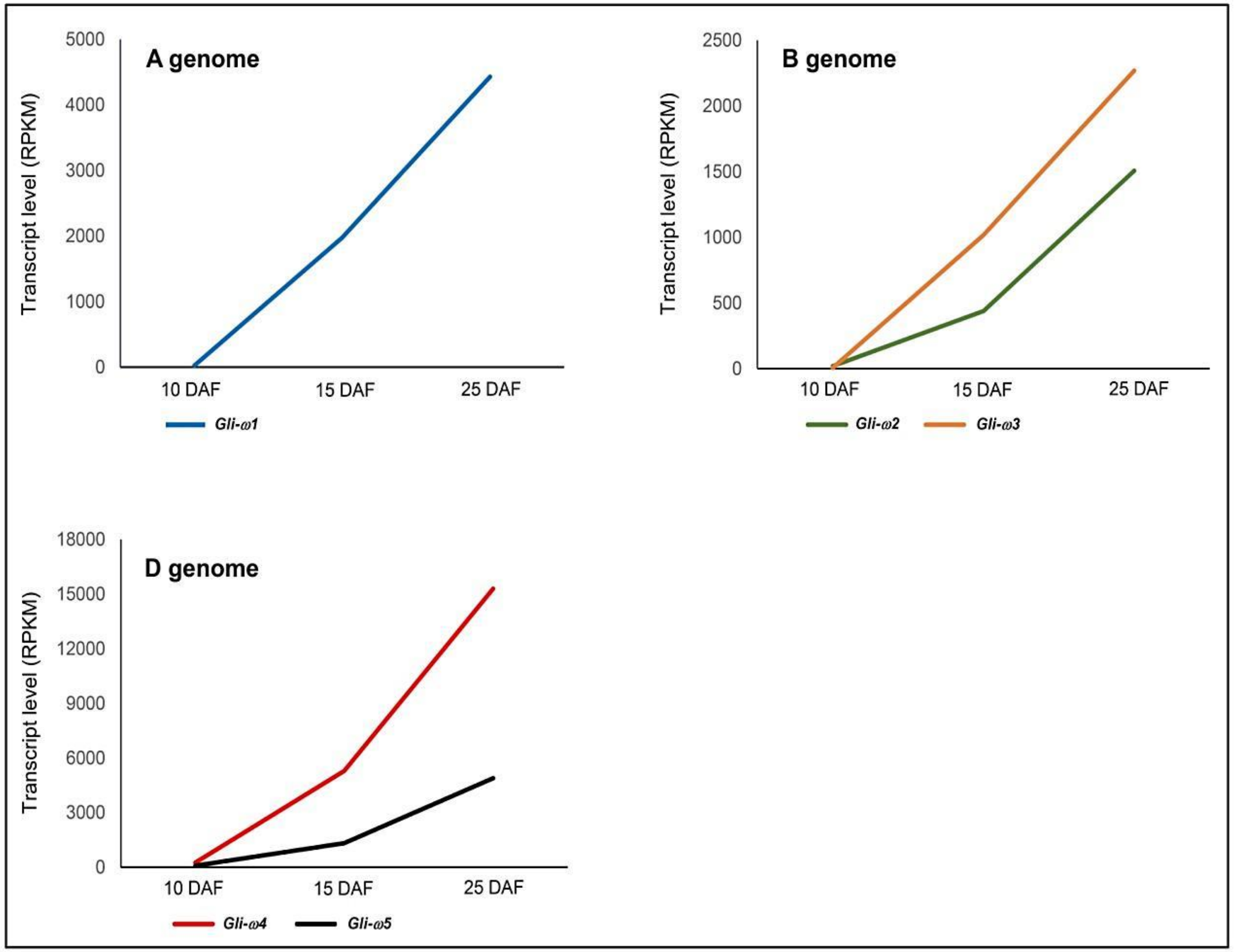

A genome
Transcript level (RPKM)
5000
4000
3000
2000
1000
0
10 DAF
15 DAF
25 DAF
Gli-ω1
B genome
Transcript level (RPKM)
2500
2000
1500
1000
500
0
10 DAF
15 DAF
25 DAF
Gli-ω2
Gli-ω3
D genome
Transcript level (RPKM)
18000
15000
12000
9000
6000
3000
0
10 DAF
15 DAF
25 DAF
Gli-ω4
Gli-ω5

Figure S7

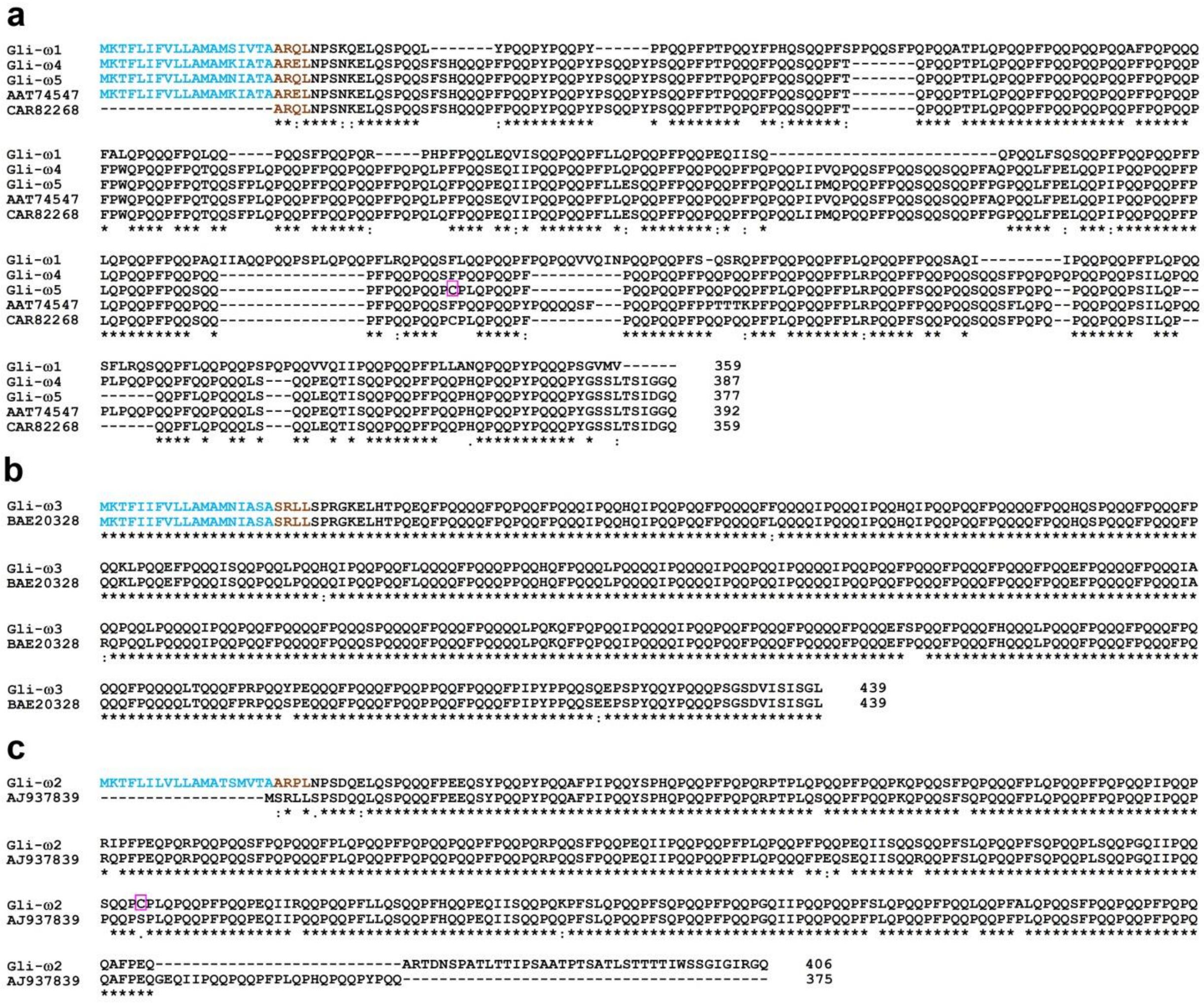
a
Gli-ω1
Gli-ω4
Gli-ω5
AAT74547
CAR82268
359
387
377
392
359
b
Gli-ω3
BAE20328
439
439
c
Gli-ω2
AJ937839
406
375

Figure S8

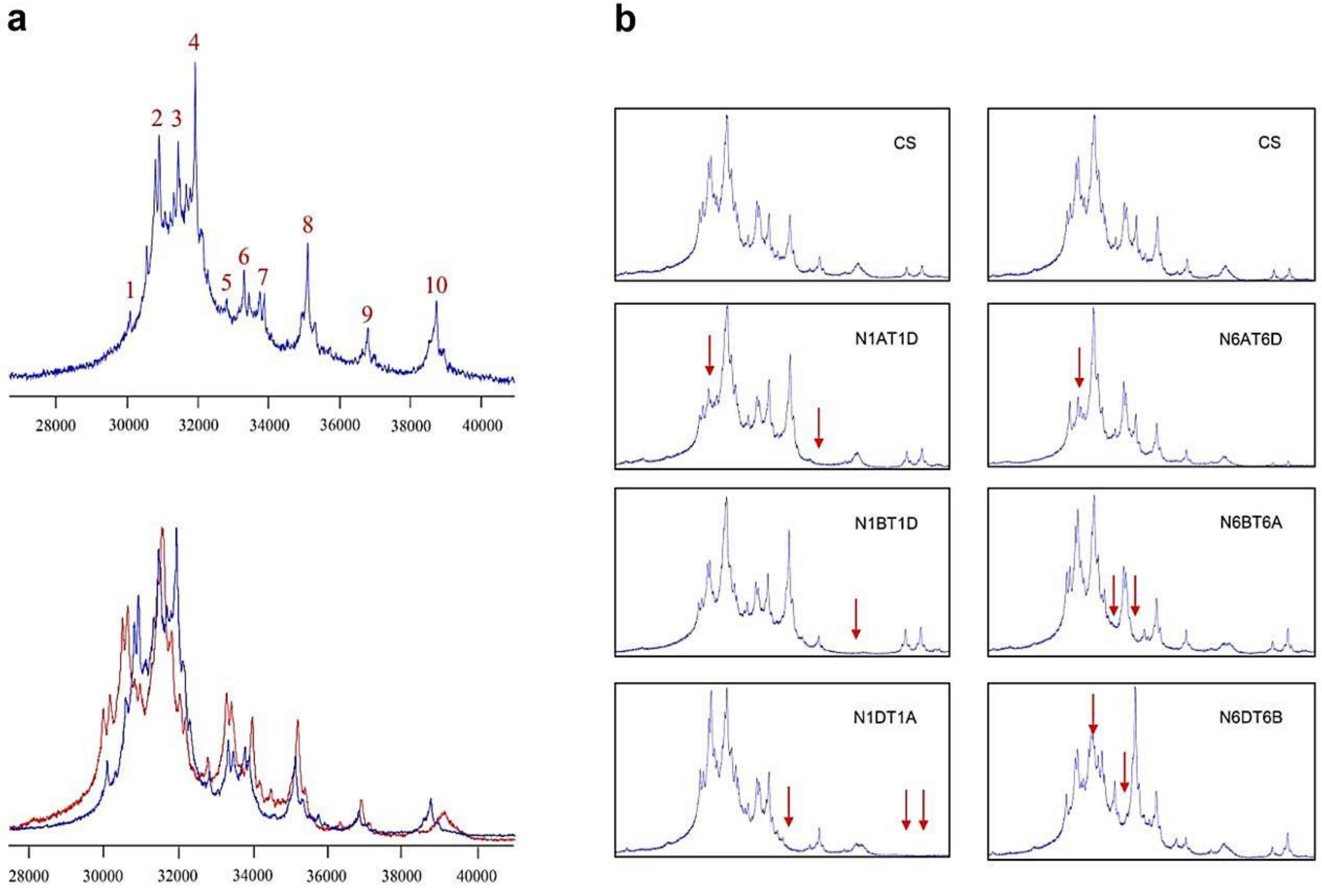
a
1
2
3
4
5
6
7
8
9
10
28000
30000
32000
34000
36000
38000
40000
b
CS
CS
N1AT1D
N6AT6D
N1BT1D
N6BT6A
N1DT1A
N6DT6B

Figure S9

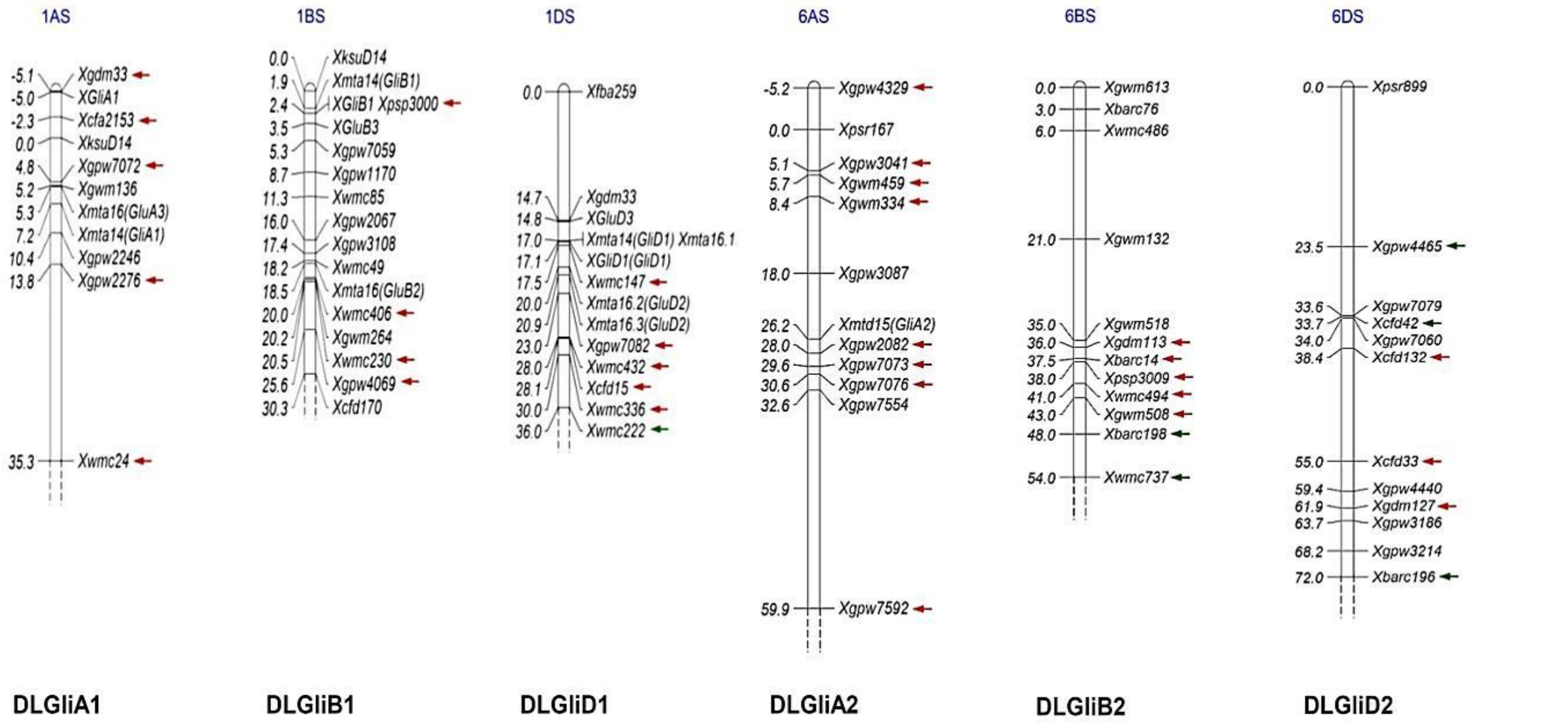
1AS
-5.1 Xgdm33
-5.0 XGliA1
-2.3 Xcfa2153
0.0 XksuD14
4.8 Xgpw7072
5.2 Xgwm136
5.3 Xmta16(GluA3)
7.2 Xmta14(GliA1)
10.4 Xgpw2246
13.8 Xgpw2276
35.3 Xwmc24
DLGliA1
1BS
0.0 XksuD14
1.9 Xmta14(GliB1)
2.4 XGliB1 Xpsp3000
3.5 XGluB3
5.3 Xgpw7059
8.7 Xgpw1170
11.3 Xwmc85
16.0 Xgpw2067
17.4 Xgpw3108
18.2 Xwmc49
18.5 Xmta16(GluB2)
20.0 Xwmc406
20.2 Xgwm264
20.5 Xwmc230
25.6 Xgpw4069
30.3 Xcfd170
DLGliB1
1DS
0.0 Xfba259
14.7 Xgdm33
14.8 XGluD3
17.0 Xmta14(GliD1) Xmta16.1
17.1 XGliD1(GliD1)
17.5 Xwmc147
20.0 Xmta16.2(GluD2)
20.9 Xmta16.3(GluD2)
23.0 Xgpw7082
28.0 Xwmc432
28.1 Xcfd15
30.0 Xwmc336
36.0 Xwmc222
DLGliD1
6AS
-5.2 Xgpw4329
0.0 Xpsr167
5.1 Xgpw3041
5.7 Xgwm459
8.4 Xgwm334
18.0 Xgpw3087
26.2 Xmtd15(GliA2)
28.0 Xgpw2082
29.6 Xgpw7073
30.6 Xgpw7076
32.6 Xgpw7554
59.9 Xgpw7592
DLGliA2
6BS
0.0 Xgwm613
3.0 Xbarc76
6.0 Xwmc486
21.0 Xgwm132
35.0 Xgwm518
36.0 Xgdm113
37.5 Xbarc14
38.0 Xpsp3009
41.0 Xwmc494
43.0 Xgwm508
48.0 Xbarc198
54.0 Xwmc737
DLGliB2
6DS
0.0 Xpsr899
23.5 Xgpw4465
33.6 Xgpw7079
33.7 Xcfd42
34.0 Xgpw7060
38.4 Xcfd132
55.0 Xcfd33
59.4 Xgpw4440
61.9 Xgdm127
63.7 Xgpw3186
68.2 Xgpw3214
72.0 Xbarc196
DLGliD2

Figure S10

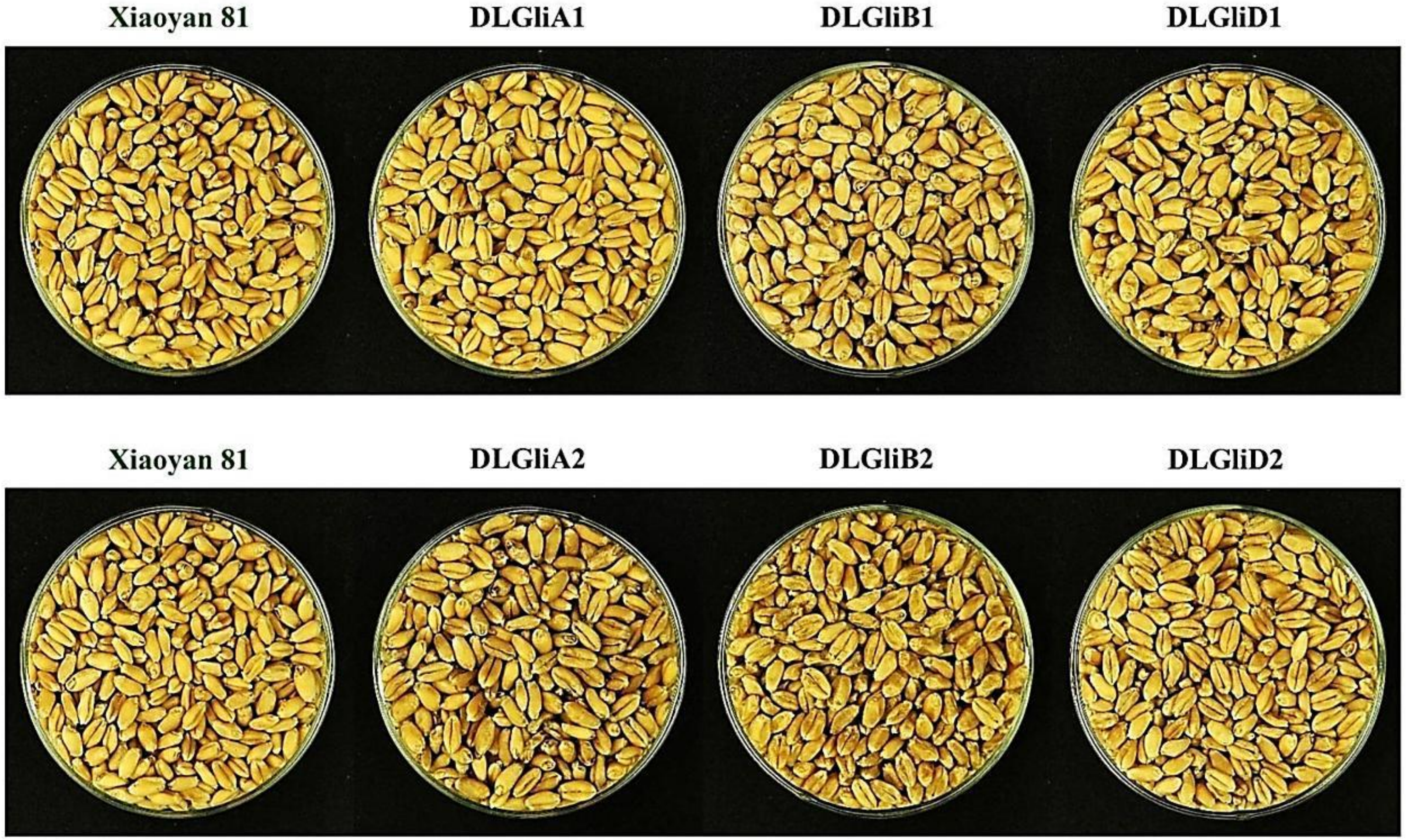
Xiaoyan 81
DLGliA1
DLGliB1
DLGliD1
Xiaoyan 81
DLGliA2
DLGliB2
DLGliD2

Figure S11

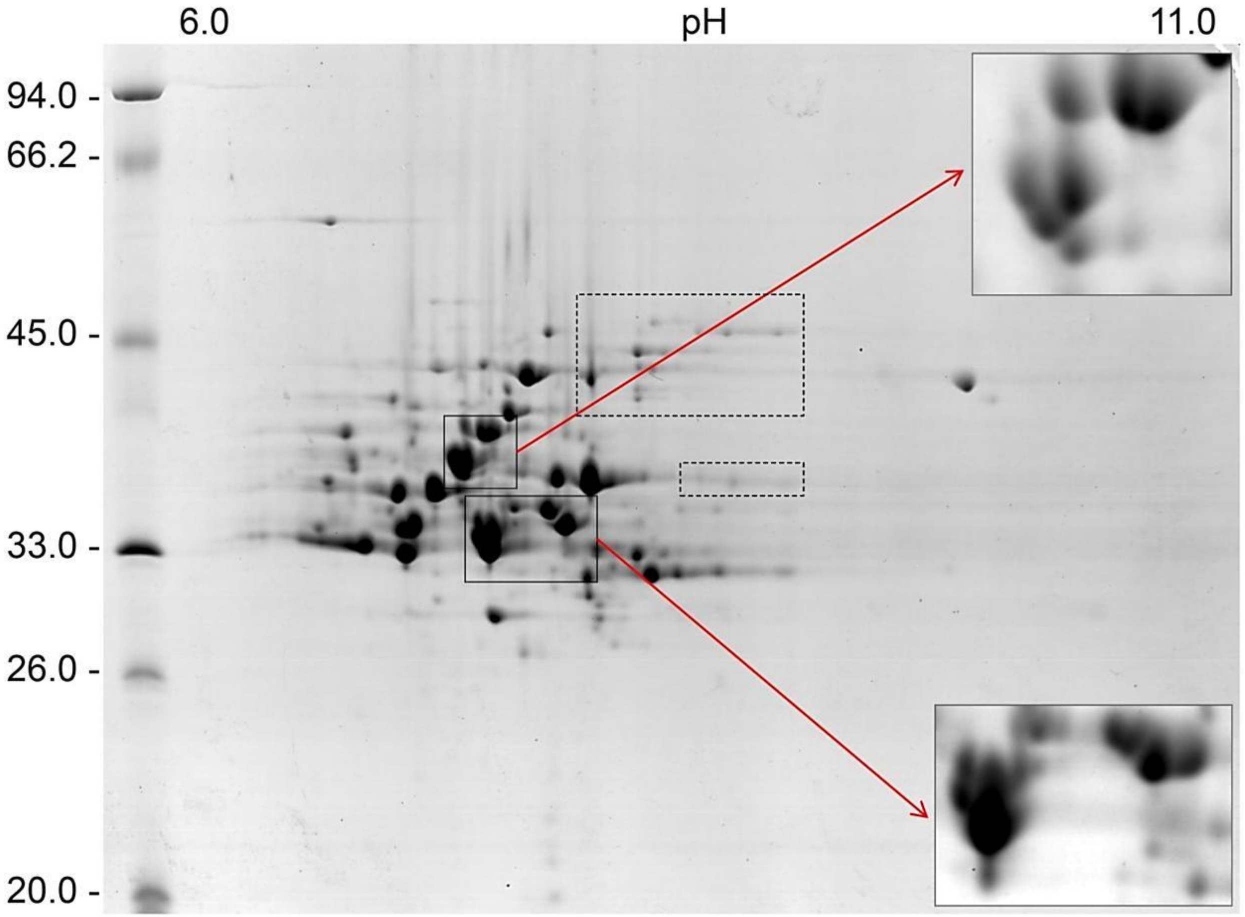

Figure S12

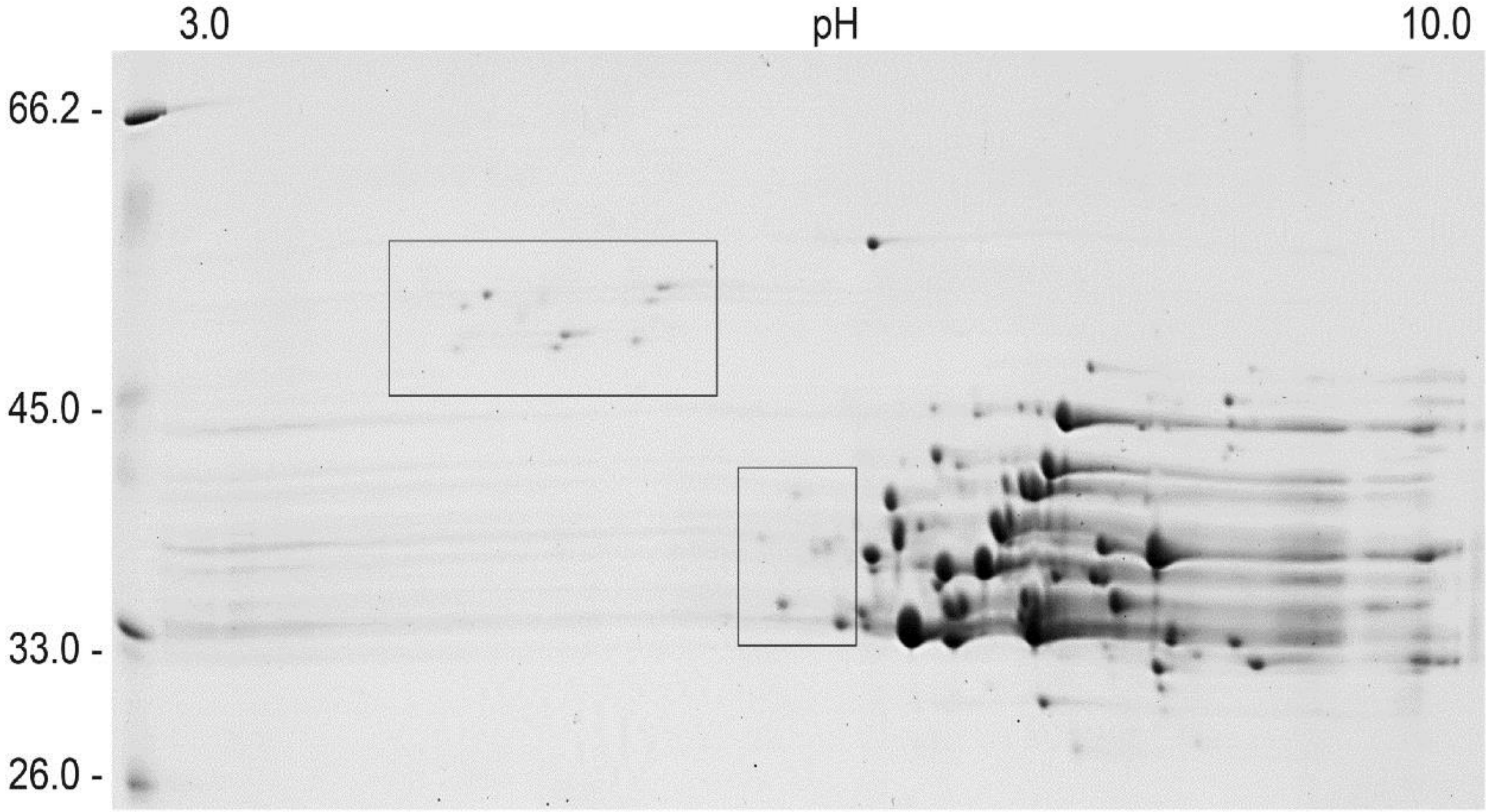

Figure S13

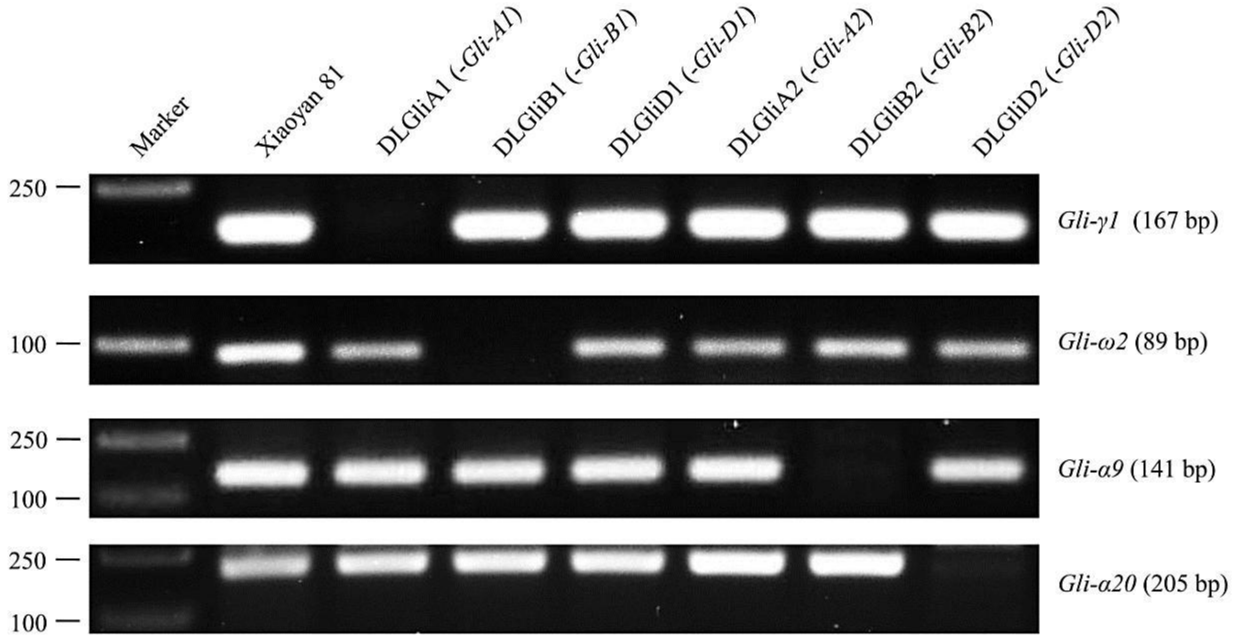
Marker
Xiaoyan 81
DLGliA1 (-Gli-A1)
DLGliB1 (-Gli-B1)
DLGliD1 (-Gli-D1)
DLGliA2 (-Gli-A2)
DLGliB2 (-Gli-B2)
DLGliD2 (-Gli-D2)
250
100
250
100
250
100
Gli-γ1 (167 bp)
Gli-ω2 (89 bp)
Gli-α9 (141 bp)
Gli-α20 (205 bp)

Figure S14

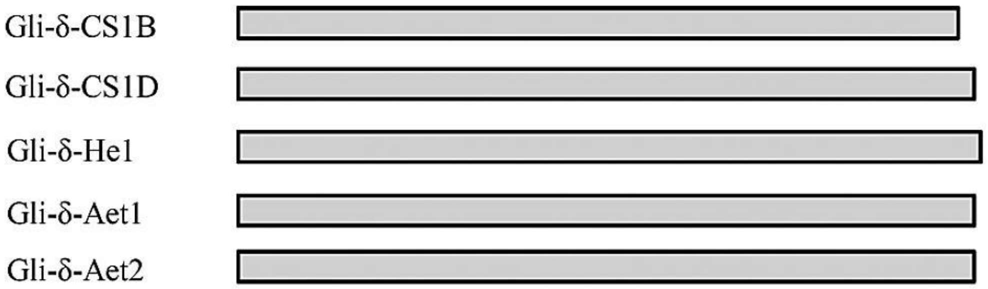

Table S1. Summary of the data of RNA sequencing experiment 3 (RSE3).

Number of cDNA library sequenced	Three (1-2 kb, 2-3 kb, and > 3 kb)
Total number of full-length non-chimeric reads obtained	187,279
Total number of unique transcripts identified	41,611
Number of transcripts mapped to Chinese Spring draft genome sequences	35,380
Number of extant chromosomal loci covered	14,747 (by 25,011 transcripts)
Number of new chromosomal loci identified	5,449 (by 10,369 transcripts)
Total number chromosomal loci mapped	20,916

Note: RSE3 was executed using the PacBio RSII platform. The three cDNA libraries sequenced were constructed with the RNA sample extracted from Xiaoyan 81 grains at 25 days after flowering.

Table S2. Sequence information of the 52 gliadin genes transcribed in the grains of Xiaoyan 81.

	Sequence
**Active members**	1) Transcript and deduced protein sequences for 25 transcribed and intact α-gliadin genes (each transcript sequence is provided from start to stop codons)<br><br>> *Gli-α1* transcript (882 bp)<br>ATGAAGACCTTTCTCATCCTTGCCCTCCTTGCTATCGTGGCGACCACCGCCACAACTGCAGTTAGAGTTCCAGTGCCACAATTGCAGCCACAAA<br>ATCCATCTCAGCAACAGCCACAAGAGCAAGTTCCGTTGGTACAACAACAACAATTTCTAGGGCAGCAACAACCATTTCCACCACAACAACCATA<br>TCCACAGCCGCAACCATTTCCATCACAACAACCATATCTGCAACTGCAACCATTTCCGCAGCCGCAACTATCATATTCGCAGCCACAACCATTT<br>CGACCACAACAACCATATCCACAACCGCAACCACAGTATTCGCAACCACAACAACCAATTTCACAGCAGCAGCAGCAGCAACAACAACAACAAC<br>AACAACAACAACAACAACAACAACAACAACAACAAGAACAACAAATCCTTCAACAAATTTTGCAACAACAACTGATTCCATGCATGGATGTTGT<br>ATTGCAGCAACACAACATAGCGCATGGAAGATCACAAGTTTTGCAACAAAGTACTTACCAGCTGTTGCAAGAATTGTGTTGTCAGCACCTATGG<br>CAGATCCCTGAGCAGTCGCAGTGCCAGGCCATCCACAATGTTGTTCATGCTATTATTCTGCATCAACAACAAAAACAACAACAACAACCATCGA<br>GCCAGGTCTCCTTCCAACAGCCTCTGCAACAATATCCATTAGGCCAGGGCTCCTTCCGGCCATCTCAGCAAAACCCACAGGCCCAGGGCTCTGT<br>CCAGCCTCAACAACTGCCCCAGTTCGAGGAAATAAGGAACCTAGCGCTACAGACGCTACCTGCAATGTGCAATGTTTACATCCCTCCATATTGC<br>ACCATGGCGCCATTTGGCATCTTCGGTACTAACTGA<br>> Gli-α1 deduced protein (293 aa)<br>MKTFLILALLAIVATTATTAVRVPVPQLQPQNPSQQQPQEQVPLVQQQFLGQQQPFPPQQPYPQPQPFPSQQPYLQLQPFPQPQLSYSQPQPF<br>RPQQPYPQPQPQYSQPQQPISQQQQQQQQQQQQQQQQQQQQQQEQQILQQILQQQLIPCMDVVLQQHNIAHGRSQVLQQSTYQLLQELCCQHLW<br>QIPEQSQCQAIHNVVHAIILHQQQKQQQQPSSQVSFQQPLQQYPLGQGSFRPSQQNPQAQGSVQPQQLPQFEEIRNLALQTLPAMCNVYIPPYC<br>TMAPFGIFGTN*<br><br>> *Gli-α2* transcript (882 bp)<br>ATGAAGACCTTTCTCATCCTTGCCCTCCTTGCTATCGTGGCGACCACCGCCACAACTGCAGTTAGAGTTCCAGTGCCACAATTGCAGCCACAAA<br>ATCCATCTCAGCAACAGCCACAAGAGCAAGTTCCGTTGGTACAACAACAACAATTTCTAGGGCAGCAACAACCATTTCCACCACAACAACCATA<br>TCCACAGCCGCAACCATTTCCATCACAACAACCATATCTGCAACTGCAACCATTTCCGCAGCCGCAACTATCATATTCGCAGCCACAACCATTT<br>CGACCACAACAACCATATCCACAACCGCAACCACAGTATTCGCAACCACAACAACCAATTTCACAGCAGCAGCAGCAGCAACAACAACAACAAC<br>AACAACAACAACAACAACAACAACAACAACAACAAGAACAACAAATCCTTCAACAAATTTTGCAACAACAACTGATTCCATGCATGGATGTTGT<br>ATTGCAGCAACACAACATAGCGCATGGAAGATCACAAGTTTTGCAACAAAGTACTTACCAGCTGTTGCAAGAATTGTGTTGTCAGCACCTATGG<br>CAGATCCCTGAGCAGTCGCAGTGCCAGGCCATCCACAATGTTGTTCATGCTATTATTCTGCATCAACAACAAAAACAACAACAACAACCATCGA<br>GCCAGGTCTCCTTCCAACAGCCTCTGCAACAATATCCATTAGGCCAGGGCTCCTTCCGGCCATCTCAGCAAAACCCACAGGCCCAGGGCTCTGT

CCAGCCTCAACAACTGCCCCAGTTCGAGGAAATAAGGAACCTAGCGCTACAGACGCTACCTGCAATGTGCAATGTCTACATCCCTCCATATTGC
ACCATCGCGCCATTTGGCATCTTCGGTACTAACTGA
>Gli-α2 deduced protein (293 aa)
MKTFLILALLAIVATTATTAVRVPVPQLQPQNPSQQQPQEQVPLVQQQQFLGQQQPFPPQQPYPQPQPFPSQQPYLQLQPFPQPQLSYSQPQPF
RPQQPYPQPQPQYSQPQQPISQQQQQQQQQQQQQQQQQQQQQQEQQILQQILQQQLIPCMDVVLQQHNIAHGRSQVLQQSTYQLLQELCCQHLW
QIPEQSQCQAIHNVVHAIILHQQQKQQQQPSSQVSFQQPLQQYPLGQGSFRPSQQNPQAQGSVQPQQLPQFEEIRNLALQTLPAMCNVYIPPYC
TIAPFGIFGTN*

>*Gli-α3* transcript (864 bp)
ATGAAGACCTTTCTCATCCTTGCCCTCCTTGCTATCGTGGCGACCACCGCCACAACTGCAGTTAGAGTTCCAGTGCCACAATTGCAGCCACAAA
ATCCATCTCAGCAACAGCCACAAGAGCAAGTTCCATTGGTACAACAACAACAATTTCTAGGGCAGCAACAACCATTTCCACCACAACAACCATA
TCCACAGCCGCAACCATTTCCATCACAACAACCATATCTGCAGCTGCAACCATTTCCGCAGCCGCAACTACCATATTCACAGCCACAACCATTT
CGACCACAACAACCATATCCACAACCGCAACCACAGTATTCGCAACCACAACAACCAATTTCACAGCAGCAGCAGCAGCAGCAACAACAACAAC
AACAACAACAACAACAACAACAACAAATCCTTCAACAAATTTTGCAACAACAACTGATTCCATGCATGGATGTTGTATTGCAGCAACACAACAT
AGCGCATGGAAGATCACAAGTTTTGCAACAAAGTACTTACCAGCTGCTGCAAGAATTGTGTTGTCAGCACCTATGGCAGATCCCTGAGCAGTCG
CAGTGCCAGGCCATCCACAATGTTGTTCATGCTATTATTCTGCATCAACAACAAAAACAACAACAACAACCATCGAGCCAGGTCTCCTTCCAAC
AGCCTCTGCAACAATATCCATTAGGCCAGGGCTCCTTCCGGCCATCTCAGCAAAACCCACAGGCCCAGGGCTCTGTCCAGCCTCAACAACTGCC
CCAGTTCGAGGAAATAAGGAACCTAGCGCTACAGACGCTACCTGCAATGTGCAATGTCTACATCCCTCCATATTGCACCATCGCGCCATTTGGC
ATCTTCGGTACTAACTGA
>Gli-α3 deduced protein (287 aa)
MKTFLILALLAIVATTATTAVRVPVPQLQPQNPSQQQPQEQVPLVQQQQFLGQQQPFPPQQPYPQPQPFPSQQPYLQLQPFPQPQLPYSQPQPF
RPQQPYPQPQPQYSQPQQPISQQQQQQQQQQQQQQQQQQQILQQILQQQLIPCMDVVLQQHNIAHGRSQVLQQSTYQLLQELCCQHLWQIPEQS
QCQAIHNVVHAIILHQQQKQQQQPSSQVSFQQPLQQYPLGQGSFRPSQQNPQAQGSVQPQQLPQFEEIRNLALQTLPAMCNVYIPPYCTIAPFG
IFGTN*

>*Gli-α4* transcript (864 bp)
ATGAAGACCTTTCTCATCCTTGCCCTCCTTGCTATCGTGGCGACCACCGCCACAACTGCAGTTAGAGTTCCAGTGCCACAATTGCAGCCACAAA
ATCCATCTCAGCAACAGCCACAAGAGCAAGTTCCATTGGTACAACAACAACAATTTCTAGGGCAGCAACAACCATTTCCACCACAACAACCATA
TCCACAGCCGCAACCATTTCCATCACAACAACCATATCTGCAGCTGCAACCATTTCTGCAGCCGCAACTACCATATTCACAGCCACAACCATTT
CGACCACAACAACCATATCCACAACCACAACCACAGTATTCGCAAGCACAACAACCAATTTCACAGCAGCAGCAGCAGCAGCAACAACAACAAC
AACAACAACAACAACAACAACAACAAATCCTTCAACAAATTTTGCAACAACAACTGATTCCATGCATGGATGTTGTATTGCAGCAACACAACAT
AGCCCATGGAAGATCACAAGTTTTGCAACAAAGTACTTACCAGCTGCTGCAAGAATTGTGTTGTCAGCACCTATGGCAGATCCCTGAGCAGTCG
CAGTGCCAGGCCATCCACAATGTTGTTCATGCTATTATTCTGCATCAACAACAAAAACAACAACAACAACCATCGAGCCAGGTCTCCTTCCAAC

AGCCTCTGCAACAATATCCATTAGGCCAGGGCTCCTTCCGGCCATCTCAGCAAAACCCACAGGCCCAGGGCTCTGTCCAGCCTCAACAACTGCC
CCAGTACGAGGAAATAAGGAACCTAGCGCTACAGACGCTACCTGCAATGTGCAATGTCTACATCCCTCCATATTGCACCATCGCGCCATTTGGC
ATCTTCGGTACTAACTGA

>Gli-α4 deduced protein (287 aa)

MKTFLILALLAIVATTATTAVRVPVPQLQPQNPSQQQPQEQVPLVQQQQFLGQQQPFPPQQPYPQPQPFPSQQPYLQLQPFLQPQLPYSQPQPF
RPQQPYPQPQPQYSQAQQPISQQQQQQQQQQQQQQQQQQILQQILQQQLIPCMDVVLQQHNIAHGRSQVLQQSTYQLLQELCCQHLWQIPEQS
QCQAIHNVVHAIILHQQQKQQQQPSSQVSFQQPLQQYPLGQGSFRPSQQNPQAQGSVQPQQLPQYEEIRNLALQTLPAMCNVYIPPYCTIAPFG
IFGTN*

>*Gli-α5* transcript (861 bp)

ATGAAGACATTTCTCATCCTTGCCCTCCTTGCTATTGTGGCGACCACCGCCACAACTGCAGTTAGAGTTCCAGTGCCACAATTGCAGCCACAAA
ATCCATCTCAGCAACAGCCACAAGAGCAAGTTCCATTGGTACAACAACAACAATTTCTAGGGCAGCAACAACCATTTCCACCACAACAACCATA
TCCACAGCCGCAACCATTTCCATCACAACAACCATATCTGCAGCTGCAACCATTTCCGCAGCCGCAACTACCATATTCGCAGCCACAACCATTT
CGACCACAACAACCATATCCACAACCGCAACCACAGTATTCGCAACCACAACAACCAATTTCACAGCAGCAGCAGCAGCAGCAACAACAACAAC
AACAACAACAACAAGAACAACAAATCCTTCAACAAATTTTGCAACAACAACTGATTCCATGCATGGATGTTGTATTGCAGCAACACAACTTAGC
GCATGGAAGATCACAAGTTTTGCAACAAAGTACTTACCAGCTGTTGCAAGAATTGTGTTGTCAGCACCTATGGCAGATCCCTGAGCAGTCGCAG
TGCCAGGCCATCCACAATGTTGTTCATGCTATTATTCTGCATCAACAACAAAAGCAACAACAACAACTATCGAGCCAGGTCTCCTTCCAACAGC
CTCAGCAACAATATCCATTAGGCCAGGGCTCCTTCCGGCCATCTCAGCAAAACTCACAGGCCCAGGGCTCTGTCCAGCCTCAACAACTGCCCCA
GTTCGAGGAAATAAGGAACCTAGCGCTACAGACCCTACCTGCAATGTGCAATGTCTACATCCCTCCATATTGCACCATCGCGCCATTTGGCATC
TTCGGTACTAAATGA

>Gli-α5 deduced protein (286 aa)

MKTFLILALLAIVATTATTAVRVPVPQLQPQNPSQQQPQEQVPLVQQQQFLGQQQPFPPQQPYPQPQPFPSQQPYLQLQPFPQPQLPYSQPQPF
RPQQPYPQPQPQYSQPQQPISQQQQQQQQQQQQQQQEQQILQQILQQQLIPCMDVVLQQHNLAHGRSQVLQQSTYQLLQELCCQHLWQIPEQSQ
CQAIHNVVHAIILHQQQKQQQQLSSQVSFQQPQQQYPLGQGSFRPSQQNSQAQGSVQPQQLPQFEEIRNLALQTLPAMCNVYIPPYCTIAPFGI
FGTK*

>*Gli-α6* transcript (870 bp)

ATGAAGACATTTCTCATCCTTGCCCTCCTTGCTATCGTGGCGACCACCGCCACAAATGCAGTTAGAGTTCCAGTGCCACAATTGCAGCCACAAA
ATCCATCTCAGCAACAGCCACAAGAGCAAGTTCCATTGGTACAACAACAACAATTTCTAGGGCAGCAACAACCATTTCCACCACAACAACCATA
TCCACAGCCGCAACCATTTCCATCACAACAACCATATCTGCAGCTGCAACCATTTTCGCAGCCGCAACTACCATATTCGCAGCCACAACCATTT
CGACCACAACAACCATATCCACAACCGCAACCACAGTATTCGCAACCACAACAACCAATTTCACAGCAGCAGCAGCAGCAGCAACAACAACAAC
AACAACAACAACAACAACAACAAGAACAACAAATCCTTCAACAAATTTTGCAACAACAACTGATTCCATGCATGGATGTTGTATTGCAGCAACA
CAACATAGCGCGTGGAAGATCACAAGTTTTGCAACAAAGTACTTACCAGCTGTTGCAAGAATTGTGTTGTCAGCACCTATGGCAGATCCCTGAG

	AAGTTGCAGTGCCAGGCCATCCACAATGTTGTTCATGCTATTATTCTGCATCAACAACAAAAACAACAACAACAACTATCGAGCCAGGTCTCCT<br>TCCAACAGCCTCAGCAACAATATCCATTAGGCCAGGGCTCCTTCCGGCCATCTCAGCAAAACCCACAGGCCCAGGGCTCTGTCCAGCCTCAACA<br>ACTGCCCCAGTTCGAGGAAATAAGGAACCTAGCGCTACAGACGTTACCTGCAATGTGCAATGTCTACATCCCTCCATATTGCACCATCGCGCCA<br>TTTGGCATCTTCGGTACTAATTGA<br>>Gli-α6 deduced protein (289 aa)<br>MKTFLILALLAIVATTATNAVRVPVPQLQPQNPSQQQPQEQVPLVQQQQFLGQQQPFPPQQPYPQPQPFPSQQPYLQLQPFSQPQLPYSQPQPF<br>RPQQPYPQPQPQYSQPQQPISQQQQQQQQQQQQQQQQQQEQQILQQILQQQLIPCMDVVLQQHNIARGRSQVLQQSTYQLLQELCCQHLWQIPE<br>KLQCQAIHNVVHAIILHQQQKQQQQLSSQVSFQQPQQQYPLGQGSFRPSQQNPQAQGSVQPQQLPQFEEIRNLALQTLPAMCNVYIPPYCTIAP<br>FGIFGTN*<br><br>>*Gli-α7* transcript (864 bp)<br>ATGAAAACCTTTCTCATCCTTGCCCTCCTTGCTATCGTGGCGACCACCGCCACAACTGCAGTTAGAGTTCCAGTGCCACAATTGCAGCCACAAA<br>ATCCATCTCAGCAACAGCCACAAGAGCAAGTTCCATTGGTACAACAACAACAATTTCTAGGGCAGCAACAACCATTTCCACCACAACAACCATA<br>TCCACAGCCGCAACCATTTCCATCACAACAACCATATCTGCAACTGCAACCATTTCTGCAGCCGCAACTACCATATTCACAGCCACAACCATTT<br>CGACCACAACAACCATATCCACAACCGCAACCACAGTATTCGCAACCACAACAACCAATTTCACAGCAGCAGCAGCAGCAGCAGCAACAACAAC<br>AACAACAACAACAACAACAACAACAAATCATTCAACAAATTTTGCAACAACAACTGATTCCATGCATGGATGTTGTATTGCAGCAACACAACAT<br>AGTGCATGGAAAATCACAAGTTTTGCAACAAAGTACTTACCAGCTGCTGCAAGAATTGTGTTGTCAGCACCTATGGCAGATCCCTGAGCAGTCG<br>CAGTGCCAGGCCATCCACAATGTTGTTCATGCTATTATTCTGCATCAACAACAAAAACAACAACAACAACCATCGAGCCAGGTCTCCTTCCAAC<br>AGCCTCTGCAACAATATCCATTAGGCCAGGGCTCCTTCCGGCCATCTCAGCAAAACCCACAGGCCCAGGGCTCTGTCCAGCCTCAACAACTGCC<br>CCAGTTCGAGGAAATAAGGAACCTAGCCCTACAGACGCTACCTGCAATGTGCAATGTCTACATCCCTCCATATTGCACCATCGCGCCATTTGGC<br>ATCTTCGGTACTAACTGA<br>>Gli-α7 deduced protein (287 aa)<br>MKTFLILALLAIVATTATTAVRVPVPQLQPQNPSQQQPQEQVPLVQQQQFLGQQQPFPPQQPYPQPQPFPSQQPYLQLQPFLQPQLPYSQPQPF<br>RPQQPYPQPQPQYSQPQQPISQQQQQQQQQQQQQQQQQQQIIQQILQQQLIPCMDVVLQQHNIVHGKSQVLQQSTYQLLQELCCQHLWQIPEQS<br>QCQAIHNVVHAIILHQQQKQQQQPSSQVSFQQPLQQYPLGQGSFRPSQQNPQAQGSVQPQQLPQFEEIRNLALQTLPAMCNVYIPPYCTIAPFG<br>IFGTN*<br><br>>*Gli-α8* transcript (891 bp)<br>ATGAAGACCTTTCTCATCCTTGCCCTCCTTGCTATCGTGGCGACCACCGCCACAACTGCAGTTAGAGTTCCAGTGCCACAACCGCAGCCACAAA<br>ATCCATCTCAGCCACAGCCACAAGGGCAAGTTCCATTGGTACAACAACAACAATTTCCAGGGCAGCAACAACAATTTCCACCACAACAGCCATA<br>TCCGCAGCCGCAACCATTTCCATCACAACAACCATATCTGCAATTGCAACCATTTCCACAACCGCAACCATTTCCGCCACAACTACCATATCCG<br>CAGCCACCACCATTTTCACCACAACAACCATATCCACAACCGCAACCACAGTATCCGCAACCACAACAACCAATTTCACAGCAACAAGCACAAC<br>AACAACAACAACAACAACAACAACAACAGCAACAACAACAACAACAACAAATCCTTCAACAAATTTTGCAACAACAACTGATTCCATGCAGGGA

TGTTGTCTTGCAACAACACAACATAGCGCATGCAAGATCACAAGTTTTGCAACAAAGCACTTACCAGCCATTGCAACAATTGTGTTGTCAACAG
CTGTGGCAGATCCCCGAGCAGTCGCGGTGCCAAGCCATCCACAATGTTGTTCATGCTATTATTCTGCATCAACAACAGCGACAACAACAACCAT
CGAGCCAGGTCTCCTTGCAACAGCCTCAGCAACAATATCCATCAGGTCAGGGCTTCTTCCAGCCATCTCAGCAAAACCCACAGGCCCAGGGCTC
TGTCCAACCTCAACAATTGCCCCAGTTCGAGGAAATAAGGAACCTAGCGCTACAGACACTACCTAGAATGTGCAATGTCTATATCCCTCCATAT
TGCTCGACCACCATTGCGCCATTTGGCATCTTCGGTACTAACTGA

> Gli-α8 deduced protein (296 aa)

MKTFLILALLAIVATTATTAVRVPVPQPQPQNPSQPQPQGQVPLVQQQQFPGQQQQFPPQQPYPQPQPFPSQQPYLQLQPFPQPQPFPPQLPYP
QPPPFSPQQPYPQPQPQYPQPQQPISQQQAQQQQQQQQQQQQQQQQQQILQQILQQQLIPCRDVVLQQHNIAHARSQVLQQSTYQPLQQLCCQQ
LWQIPEQSRCQAIHNVVHAIILHQQQRQQQPSSQVSLQQPQQQYPSGQGFFQPSQQNPQAQGSVQPQQLPQFEEIRNLALQTLPRMCNVYIPPY
CSTTIAPFGIFGTN*

>*Gli-α9* transcript (978 bp)

ATGAAGACCTTTCTCATCCTTTCCCTCCTTGCTATCGTGGCGACCACTGCCACAACTGCAGTTAGATTTCCAGTGCCACAATTGCAGCCGCAAA
ATCCATCTCAGCAACAACCACAAGAGCAAGTTCCGTTGGTGCAACAACTACAATATCCAGGGCAACAACAACCATTTCCACCACAACAGCCATA
TCCGCAGCCGCAACCATTTCCATCACAACAACCATTGCCGCAACCGCAACCATTTCTGCCACAACTACCATATCCGCAGCCACAACCATTTCCA
CCACAACAACCATATCCACAACCGCAACCACAGTATCCGCAACCACAACAACCAATTTCACAGCAACAAGCACAACAAGCACAACAACAACAAC
AACAACAACAACAACAACAACAACAACAAATCCTTCAACAAATTCTGCAACAACAACAACTGATTCCATGCAGGGATGTCGTCTTGCAACAACA
CAACATAGCGCATGCAAGCTCACAAGTATTGCAACAAAGTAGTTACCAACTGTTGCAACAATTATGTTGTCAACGTTTGTGGCAGATCCCCGAG
CAGTCGCGGTGCCAAGCCATCCACAATGTCGTTCATGCTATTATTCTGCAACAACAACAACAACAACAACAACAACAACAACAACAACAACAAC
AACAACAACAACAACAACAACAACAACAACAACAACAACAACAACAACAACAACAACAACAACAACAACAACAACAACAACAACCGTCGAGCCA
GGTCTCCTACCAGCAGCCTCAGCAACAATATCCATCGGGCCAGGGATCCTTCCAGCCATCTCAGCAGAACCCACAGGCCCAGGCCTCTGTCCAG
CCTCAACAACTTCCCCAGTTCGAGGAAATAAGGAATCTAGCACGGCAGACGCTACCGGCAATGTGCAATGTCTACATCCCTCCATATTGCTCGA
CCACCATTGCGCCATCTGGCATCTTTGGTACTAACTGA

>Gli-α9 deduced protein (325 aa)

MKTFLILSLLAIVATTATTAVRFPVPQLQPQNPSQQQPQEQVPLVQQLQYPGQQQPFPPQQPYPQPQPFPSQQPLPQPQPFLPQLPYPQPQPFP
PQQPYPQPQPQYPQPQQPISQQQAQQAQQQQQQQQQQQQQQQILQQILQQQLIPCRDVVLQQHNIAHASSQVLQQSSYQLLQQLCCQRLWQIPE
QSRCQAIHNVVHAIILQQQQQQQQQQQQQQQQQQQQQQQQQQQQQQQQQQQQQQQQQQQPSSQVSYQQPQQQYPSGQGSFQPSQQNPQAQASVQ
PQQLPQFEEIRNLARQTLPAMCNVYIPPYCSTTIAPSGIFGTN*

>*Gli-α10* transcript (900 bp)

ATGAAGACCTTTCTCATCCTTGCCCTCCTTGCTATCGTGGCGACCACCACCACAACTGCAGTTAGAGTTCCAGTGCCACAATTGCAGCCACAAA
ATCCATCTCAGCAACAACCACAAGAGCAAGTTCCATTGGTGCAACAACAACAATTTCTAGGGCAGCAACAACAACAATTTCCAGGGCAACAACA
ACCATTTCCACCACAACAGCCATATCCGCAGCCGCAACCATTTCTGCCACAACTACCATATCCGCAGCCGCAACCATTTCCACCACAACAATCA

TATCCACAACCACAACCACAATATCCGCAACCACAACAACCAATTTCGCAGCAACAAGCACAACTACAACAACAACAACAACAACAACAACAAC
AACAACAACAACAACAACAACAACAACAAATCCTTCAACAAATTCTGCAACAACAACTGATTCCATGTAGGGATGTCGTCTTGCAACAACCCAA
CATAGCACATGCAAGCTCACAAGTATCGCAACAAAGTTACCAACTGTTGCAACAATTATGTTGTCAGCAACTGTGGCAGACCCCCGAGCAGTCA
CGGTGCCAAGCCATCCACAATGTCATTCATGCTATTATTTTGCATCAACAACAACAACAACAACAACAACAACAACAACAACAACAACAACAAC
AACAACCGTCGAGCCAGGTCTCCTACCAGCAGCCTCAGCAACAATATCCATCAGGCCAGGGCTCGTTCCAGCCATCTCAGCAAAACCCACAGGC
CCAGGGCTTTGTCCAACCTCAGCAACTGCCGCAGTTCGAGGAAATAAGGAACCTAGCGCTACAGACGCTACCAGCAATGTGCAATGTCTACATC
CCTCCATATTGCTCGACCACCATTGCGCCATTTGGCATCATGAGTACTAACTGA
>Gli-α10 deduced protein (299 aa)
MKTFLILALLAIVATTTTTAVRVPVPQLQPQNPSQQQPQEQVPLVQQQQFLGQQQQQFPGQQQPFPPQQPYPQPQPFLPQLPYPQPQPFPPQQS
YPQPQPQYPQPQQPISQQQAQLQQQQQQQQQQQQQQQQQQQILQQILQQLIPCRDVVLQQPNIAHASSQVSQQSYQLLQQLCCQQLWQTPEQS
RCQAIHNVIHAIILHQQQQQQQQQQQQQQQQQQPSSQVSYQQPQQQYPSGQGSFQPSQQNPQAQGFVQPQQLPQFEEIRNLALQTLPAMCNVYI
PPYCSTTIAPFGIMSTN*

>*Gli-α11* transcript (882 bp)
ATGAAGACCTTTCTCATCCTTGCCCTCCTTGCTATCGTGGCGACCACCACCACAACTGCAGTTAGAGTTCCAGTGCCACAATTGCAGCCACAAA
ATCCATCTCAGCAACAACCACAAGAGCAAGTTCCATTGGTGCAACAACAACAATTTCTAGGGCAGCAACAACAACAATTTCCAGGGCAACAACA
ACCATTTCCACCACAACAGCCATATCCGCAGCCGCAACCATTTCTGCCACAACTACCATATCCGCAGCCGCAACCATTTCCACCACAACAATCA
TATCCACAACCACAACCACAATATCCGCAACCACAACAACCAATTTCGCAGCAACAAGCACAACTACAACAACAACAACAACAACAACAACAAC
AACAACAACAAATCCTTCAACAAATTCTGCAACAACAACTGATTCCATGCAGGGATGTCGTCTTGCAACAACCCAATATAGCACATGCAAGCTC
AAAAGTATCGCAACAAAGTTACCAACTGTTGCAACAATTATGTTGTCAGCAACTGTGGCAGACCCCCGAGCAGTCACGGTGCCAAGCCATCCAC
AATGTCATTCATGCTATTATTTTGCATCATCAACAACAACAACAACAACAACAACAACAACAACAACAACAACAACAACAACCGTCGAGCCAGG
TCTCCTACCAGCAGCCTCAGCAACAATATCCATCAGGCCAGGGCTTCTTCCAGCCATCTCAGCAAAACCCACAGGCCCAGGGCTTTGTCCAACC
TCAGCAACTGCCGCAGTTCGAGGAAATAAGGAACCTAGCGCTGCAGACGCTACCAGCAATGTGCAATGTCTACATCCCTCCATATTGCTCGACC
ACCATTGCGCCATTTGGCATCATGAGTACTAACTGA
>Gli-α11 deduced protein (293 aa)
MKTFLILALLAIVATTTTTAVRVPVPQLQPQNPSQQQPQEQVPLVQQQQFLGQQQQQFPGQQQPFPPQQPYPQPQPFLPQLPYPQPQPFPPQQS
YPQPQPQYPQPQQPISQQQAQLQQQQQQQQQQQQQILQQILQQLIPCRDVVLQQPNIAHASSKVSQQSYQLLQQLCCQQLWQTPEQSRCQAIH
NVIHAIILHHQQQQQQQQQQQQQQQQQPSSQVSYQQPQQQYPSGQGFFQPSQQNPQAQGFVQPQQLPQFEEIRNLALQTLPAMCNVYIPPYCST
TIAPFGIMSTN*

>*Gli-α12* transcript (882 bp)
ATGAAGACCTTTCTCATCCTTGCCCTCCTTGCTATCGTGGCGACCACCACCACAACTGCAGTTAGAGTTCCAGTGCCACAATTGCAGCCACAAA
ATCCATCTCAGCAACAACCACAAGAGCAAGTTCCATTGGTGCAACAACAACAATTTCTAGGGCAGCAACAACAACAATTTCCAGGGCAACAACA

ACCATTTCCACCACAACAGCCATATCCGCAGCCGCAACCATTTCTGCCACAACTACCATATCCGCAGCCGCAACCATTTCCACCACAACAATCA
TATCCACAACCACAACCACAATATCCGCAACCACAACAACCAATTTCGCAGCAACAAGCACAACTACAACAACAACAACAACAACAACAACAAC
AACAACAACAAATCCTTCAACAAATTCTGCAACAACAACTGATTCCATGCAGGGATGTCGTCTTGCAACAACCCAATATAGCACATGCAAGCTC
ACAAGTATCGCAACAAAGTTACCAACTGTTGCAACAATTATGTTGTCAGCAACTGTGGCAGACCCCCGAGCAGTCACGGTGCCAAGCCATCCAC
AATGTCATTCATGCTATTATTTTGCATCATCAACAACAACAACAACAACAACAACAACAACAACAACAACAACAACAACAACCGTCGAGCCAGG
TCTCCTACCAGCAGCCTCAGCAACAATATCCATCAGGCCAGGGCTTCTTCCAGCCATCTCAGCAAAACCCACAGGCCCAGGGCTTTGTCCAACC
TCAGCAACTGCCGCAGTTCGAGGAAATAAGGAACCTAGCGCTGCAGACGCTACCAGCAATGTGCAATGTCTACATCCCTCCATATTGCTCGACC
ACCATTGCGCCATTTGGCATCATGAGTACTAACTGA
>Gli-α12 deduced protein (293 aa)
MKTFLILALLAIVATTTTTAVRVPVPQLQPQNPSQQQPQEQVPLVQQQQFLGQQQQQFPGQQQPFPPQQPYPQPQPFLPQLPYPQPQPFPPQQS
YPQPQPQYPQPQQPISQQQAQLQQQQQQQQQQQQQILQQILQQQLIPCRDVVLQQPNIAHASSQVSQQSYQLLQQLCCQQLWQTPEQSRCQAIH
NVIHAIILHHQQQQQQQQQQQQQQQQQPSSQVSYQQPQQQYPSGQGFFQPSQQNPQAQGFVQPQQLPQFEEIRNLALQTLPAMCNVYIPPYCST
TIAPFGIMSTN*

>*Gli-α13* transcript (891 bp)
ATGAAGACCTTTCTCATCCTTGCCCTCCTTGCTATCGTGGCGACCACCACCACAACTGCAGTTAGAGTTCCAGTGCCACAATTGCAGCCACAAA
ATCCATCTCAGCAACAACCACAAGAGCAAGTTCCATTGGTGCAACAACAACAATTTCTAGGGCAGCAACAACAACAATTTCCAGGGCAACAACA
ACCATTTCCACCACAACAGCCATATCCGCAGCCGCAACCATTTCTGCCACAACTACCATATCCGCAGCCGCAACCATTTCCACCACAACAATCA
TATCCACAACCACAACCACAATATCCGCAACCACAACAACCAATTTCGCAGCAACAAGCACAACTACAACAACAACAACAACAACAACAACAAC
AACAACAAATCCTTCAACAAATTCTGCAACAACAACTGATTCCATGCAGGGATGTCGTCTTGCAACAACCCAATATAGCACATGCAAGCTCACA
AGTATCGCAACAAAGTTACCAACTGTTGCAACAATTATGTTGTCAGCAACTGTGGCAGACCCCCGAGCAGTCACGGTGCCAAGCCATCCACAAT
GTCATTCATGCTATTATTTTGCATCAACAACAACAACAACAACAACAACAACAACAACAACAACAACAACAACAACAACAACAACAACAACCGT
CGAGCCAGGTCTCCTACCAGCAGCCTCAGCAACAATATCCATCAGGCCAGGGCTTCTTCCAGCCATCTCAGCAAAACCCACAGGCCCAGGGCTT
TGTCCAACCTCAGCAACTGCCGCAGTTCGAGGAAATAAGGAACCTAGCGCTGCAGACGCTACCAGCAATGTGCAATGTCTACATCCCTCCATAT
TGCTCGACCACCATTGCGCCATTTGGCATCATGAGTACTAACTGA
>Gli-α13 deduced protein (296 aa)
MKTFLILALLAIVATTTTTAVRVPVPQLQPQNPSQQQPQEQVPLVQQQQFLGQQQQQFPGQQQPFPPQQPYPQPQPFLPQLPYPQPQPFPPQQS
YPQPQPQYPQPQQPISQQQAQLQQQQQQQQQQQQILQQILQQQLIPCRDVVLQQPNIAHASSQVSQQSYQLLQQLCCQQLWQTPEQSRCQAIHN
VIHAIILHQQQQQQQQQQQQQQQQQQQQQQPSSQVSYQQPQQQYPSGQGFFQPSQQNPQAQGFVQPQQLPQFEEIRNLALQTLPAMCNVYIPPY
CSTTIAPFGIMSTN*

>*Gli-α14* transcript (891 bp)
ATGAAGACCTTTCTCATCCTTGCCCTCCTTGCTATCGTGGCGACCACCACCACAACTGCAGTTAGAGTTCCAGTGCCACAATTGCAGCCACAAA

ATCCATCTCAGCAACAACCACAAGAGCAAGTTCCATTGGTGCAACAACAACAATTTCTAGGGCAGCAACAACAACAATTTCCAGGGCAACAACA
ACCATTTCCACCACAACAGCCATATCCGCAGCCGCAACCATTTCTGCCACAACTACCATATCCGCAGCCGCAACCATTTCCACCACAACAATCA
TATCCACAACCACAACCACAATATCCGCAACCACAACAACCAATTTCGCAGCAACAAGCACAACTACAACAACAACAACAACAACAACAACAAC
AACAACAAATCCTTCAACAAATTCTGCAACAACAACTGATTCCATGCAGGGATGTCGTCTTGCAACAACCCAATATAGCACATGCAAGCTCACA
AGTATCGCAACAAAGTTACCAACTGTTGCAACAATTATGTTGTCTGCAACTGTGGCAGACCCCCGAGCAGTCACGGTGCCAAGCCATCCACAAT
GTCATTCATGCTATTATTTTGCATCAACAACAACAACAACAACAACAACAACAACAACAACAACAACAACAACAACAACAACAACAACAACCGT
CGAGCCAGGTCTCCTACCAGCAGCCTCAGCAACAATATCCATCAGGCCAGGGCTTCTTCCAGCCATCTCAGCAAAACCCACAGGCCCAGGGCTT
TGTCCAACCTCAGCAACTGCCGCAGTTCGAGGAAATAAGGAACCTAGCGCTGCAGACGCTACCAGCAATGTGCAATGTCTACATCCCTCCATAT
TGCTCGACCACCATTGCGCCATTTGGCATCATGAGTACTAACTGA
>Gli-α14 deduced protein (296 aa)
MKTFLILALLAIVATTTTTAVRVPVPQLQPQNPSQQQPQEQVPLVQQQFLGQQQQQFPGQQQPFPPQQPYPQPQPFLPQLPYPQPQPFPPQQS
YPQPQPQYPQPQQPISQQQAQLQQQQQQQQQQQQILQQILQQQLIPCRDVVLQQPNIAHASSQVSQQSYQLLQQLCCLQLWQTPEQSRCQAIHN
VIHAIILHQQQQQQQQQQQQQQQQQQQQQQPSSQVSYQQPQQQYPSGQGFFQPSQQNPQAQGFVQPQQLPQFEEIRNLALQTLPAMCNVYIPPY
CSTTIAPFGIMSTN*

>*Gli-α15* transcript (942 bp)
ATGAAGACCTTTCTCATCCTTGCCCTCCTTGCTATCGTGGCGACCACCGCCACAACTGCAGTTAGAGTTCCAGTGCCACAATTGCAGCCACAAA
ATCCATCTCAGCAACAGCCACAAGAGCAAGTTCCATTGGTACAACAACAACAATTTCCAGGGCAGCAACAACAATTTCCACCACAACAGCCATA
TCCGCAGCCGCAACCATTTCCATCACAACAACCATATCTGCAACTGCAACCATTTCCGCAGCCGCAACCATTTCCGCCACAACTACCATATCCG
CAGCCGCAATCATTTCCACCACAACAACCATATCCACAACAGCAACCACAGTATCTACAACCACAACAACCAATTTCGCAGCAACAAGCACAAC
AACAACAACAACAACAACAACAACAACAACAACAACAACAAATCCTTCAACAAATTTTGCAACAACAACTGATTCCATGCAGGGATGTTGTCTT
GCAACAACACAACATAGCGCATGCAAGCTCACAAGTTTTGCAACAAAGTACTTACCAGCTATTGCAACAATTGTGTTGTCAACAACTGTTGCAG
ATCCCTGAGCAGTCGAGGTGCCAAGCCATCCATAATGTTGCTCATGCTATTATTATGCATCAACAACAACAACAACAACAAGAACAACAACAAC
AGTTGCAACAACAACAACAGCAGCAACTGCATCAACAACGACAACAACCATCGAGCCAGGTCTCCTTCCAACAGCCTCAGCAGCAATATCCATC
AAGCCAGGTCTCCTTCCAGCCATCTCAGCTAAACCCACAGGCTCAGGGCTCTGTCCAACCTCAACAACTGCCCCAGTTCGCGGAAATAAGGAAC
CTAGCGCTACAGACGCTACCTGCAATGTGCAATGTCTACATCCCTCCACATTGCTCGACCACCATTGCGCCATTTGGCATCTTTGGTACCAACT
GA
>Gli-α15 deduced protein (313 aa)
MKTFLILALLAIVATTATTAVRVPVPQLQPQNPSQQQPQEQVPLVQQQFPGQQQQFPPQQPYPQPQPFPSQQPYLQLQPFPQPQPFPPQLPYP
QPQSFPPQQPYPQQQPQYLQPQQPISQQQAQQQQQQQQQQQQQQQQILQQILQQQLIPCRDVVLQQHNIAHASSQVLQQSTYQLLQQLCCQQLLQ
IPEQSRCQAIHNVAHAIIMHQQQQQQQEQQQQLQQQQQQQLHQQRQQPSSQVSFQQPQQQYPSSQVSFQPSQLNPQAQGSVQPQQLPQFAEIRN
LALQTLPAMCNVYIPPHCSTTIAPFGIFGTN*

	>*Gli-α16* transcript (942 bp)<br>ATGAAGACCTTTCTCATCCTTGCCCTCCTTGCTATCGTGGCGACCACCGCCACAACTGCAGTTAGAGTTCCAGTGCCACAATTGCAGCCACAAA<br>ATCCATCTCAGCAACAGCCACAAGAGCAAGTTCCATTGGTACAACAACAACAATTTCCAGGGCAGCAACAACAATTTCCACCACAACAGCCATA<br>TCCGCAGCCGCAACCATTTCCATCACAACAACCATATCTGCAACTGCAACCATTTCCGCAGCCGCAACCATTTCCGCCACAACTACCATATCCG<br>CAGCCGCAATCATTTCCACCACAACAACCATATCCACAACAGCAACCACAGTATCTACAACCACAACAACCAATTTCGCAGCAACAAGCACAAC<br>AACAACAACAACAACAACAACAACAACAACAACAACAACAAATCCTTCAACAAATTTTGCAACAACAACTGATTCCATGCAGGGATGTTGTCTT<br>GCAACAACACAACATAGCGCATGCAAGCTCACAAGTTTTGCAACAAAGTACTTACCAGCTATTGCAACAATTGTGTTGTCAACAACTGTTGCAG<br>ATCCCTGAGCAGTCGAGGTGCCAAGCCATCCATAATGTTGCTCATGCTATTATTATGCATCAACAACAACAACAACAACAAGAACAACAACAAC<br>AGTTGCAACAACAACAACAGCAGCAACTGCAACAACAACGACAACAACCGTCGAGCCAGGTCTCCTTCCAACAGCCTCAGCAGCAATATCCATC<br>AAGCCAGGTCTCCTTCCAGCCATCTCAGCTAAACCCACAGGCTCAGGGCTCCGTCCAACCTCAACAACTGCCCCAGTTCGCGGAAATAAGGAAC<br>CTAGCGCTACAGACGCTACCTGCAATGTGCAATGTCTACATCCCTCCACATTGCTCGACCACCATTGCGCCATTTGGCATCTTTGGTACCAACT<br>GA<br>>Gli-α16 deduced protein (313 aa)<br>MKTFLILALLAIVATTATTAVRVPVPQLQPQNPSQQQPQEQVPLVQQQQFPGQQQQFPPQQPYPQPQPFPSQQPYLQLQPFPQPQPFPPQLPYP<br>QPQSFPPQQPYPQQQPQYLQPQQPISQQQAQQQQQQQQQQQQQQQQILQQILQQQLIPCRDVVLQQHNIAHASSQVLQQSTYQLLQQLCCQQLLQ<br>IPEQSRCQAIHNVAHAIIMHQQQQQQQEQQQQLQQQQQQQLQQQRQQPSSQVSFQQPQQQYPSSQVSFQPSQLNPQAQGSVQPQQLPQFAEIRN<br>LALQTLPAMCNVYIPPHCSTTIAPFGIFGTN*<br><br>>*Gli-α17* transcript (894 bp)<br>ATGAAGACCTTTCTCATCCTTGCCCTCCTTGCTATCGTGGCGACCACCACCACAACTGCAGTTAGAGTTCCAGTGCCACAATTGCAGCCACAAA<br>ATCCATCTCAGCAACAACCACAAGAGCAAGTTCCATTGGTGCAACAACAACAATTTCTAGGGCAGCAACAACAACAATTTCCAGGGCAACAACA<br>ACCATTTCCACCACAACAGCCATATCCGCAGCCGCAACCATTTCTGCCACAACTACCATATCCGCAGCCGCAACCATTTCCACCACAACAATCA<br>TATCCACAACCACAACCACAATATCCGCAACCACAACAACCAATTTCGCAGCAACAAGCACAACTACAACAACAACAACAACAACAACAACAAC<br>AACAACAAATCCTTCAACAAATTCTGCAACAACAACTGATTCCATGCAGGGATGTCGTCTTGCAACAACCCAATATAGCACATGCAAGCTCAAA<br>AGTATCGCAACAAAGTTACCAACTGTTGCAACAATTATGTTGTCTGCAACTGTGGCAGACCCCCGAGCAGTCACGGTGCCAAGCCATCCACAAT<br>GTCATTCATGCTATTATTTTGCATCATCAACAACAACAACAACAACAACAACAACAACAACAACAACAACAACAACAACAACAACAACAACAAC<br>CGTCGAGCCAGGTCTCCTACCAGCAGCCTCAGCAACAATATCCATCAGGCCAGGGCTTCTTCCAGCCATCTCAGCAAAACCCACAGGCCCAGGG<br>CTTTGTCCAACCTCAGCAACTGCCGCAGTTCGAGGAAATAAGGAACCTAGCGCTGCAGACGCTACCAGCAATGTGCAATGTCTACATCCCTCCA<br>TATTGCTCGACCACCATTGCGCCATTTGGCATCATGAGTACTAACTGA<br>>Gli-α17 deduced protein (297 aa)<br>MKTFLILALLAIVATTTTTAVRVPVPQLQPQNPSQQQPQEQVPLVQQQQFLGQQQQQFPGQQQPFPPQQPYPQPQPFLPQLPYPQPQPFPPQQS<br>YPQPQPQYPQPQQPISQQQAQLQQQQQQQQQQQQILQQILQQQLIPCRDVVLQQPNIAHASSKVSQQSYQLLQQLCCLQLWQTPEQSRCQAIHN<br>VIHAIILHHQQQQQQQQQQQQQQQQQQQQQQQPSSQVSYQQPQQQYPSGQGFFQPSQQNPQAQGFVQPQQLPQFEEIRNLALQTLPAMCNVYIPP

YCSTTIAPFGIMSTN*

>*Gli-α18* transcript (939 bp)
ATGAAGACCTTTCTCATCCTTGCCCTCCTTGCTATCGTGGCGACCACCGCCACAACTGCAGTTAGAGTTCCAGTGCCACAATTGCAGCCACAAA
ATCCATCTCAGCAACAGCCACAAGAGCAAGTTCCATTGGTACAACAACAACAATTTCCAGGGCAGCAACAACAATTTCCACCACAACAGCCATA
TCCGCAGCCGCAACCATTTCCATCACAACAACCATATCTGCAACTGCAACCATTTCCGCAGCCGCAACCATTTCCGCCACAACTACCATATCCG
CAGCCGCAATCATTTCCACCACAACAACCATATCCACAACAGCAACCACAGTATCTACAACCACAACAACCAATTTCGCAGCAACAAGCACAAC
AACAACAACAACAACAACAACAACAACAACAACAACAAATCCTTCAACAAATTTTGCAACAACAACTGATTCCATGCAGGGATGTTGTCTTGCA
ACAACACAACATAGCGCATGCAAGCTCACAAGTTTTGCAACAAAGTACTTACCAGCTATTGCAACAATTGTGTTGTCAACAACTGTTGCAGATC
CCTGAGCAGTCGAGGTGCCAAGCCATCCATAATGTTGCTCATGCTATTATTATGCATCAACAACAACAACAACAACAAGAACAACAACAACAGT
TGCAACAACAACAACAGCAGCAACTGCATCAACAACGACAACAACCATCGAGCCAGGTCTCCTACCAGCAGCCTCAGCAACAATATCCATCAGG
CCAGGGCTTCTTCCAGCCATCTCAGCAAAACCCACAGGCCCAGGGCTTTGTCCAACCTCAGCAACTGCCGCAGTTCGAGGAAATAAGGAACCTA
GCGCTGCAGACGCTACCAGCAATGTGCAATGTCTACATCCCTCCATATTGCTCGACCACCATTGCGCCATTTGGCATCATGAGTACTAACTGA
>Gli-α18 deduced protein (312 aa)
MKTFLILALLAIVATTATTAVRVPVPQLQPQNPSQQQPQEQVPLVQQQQFPGQQQQFPPQQPYPQPQPFPSQQPYLQLQPFPQPQPFPPQLPYP
QPQSFPPQQPYPQQQPQYLQPQQPISQQQAQQQQQQQQQQQQQQILQQILQQQLIPCRDVVLQQHNIAHASSQVLQQSTYQLLQQLCCQQLLQI
PEQSRCQAIHNVAHAIIMHQQQQQQQEQQQQLQQQQQQQLQQQRQQPSSQVSFQQPQQQYPSSQVSFQPSQLNPQAQGSVQPQQLPQFAEIRNL
ALQTLPAMCNVYIPPHCSTTIAPFGIFGTN*

>*Gli-α19* transcript (849 bp)
ATGAAGACCTTTCTCATCCTAGCCCTCCTTGCTATCGTGGCGACCACCGCCACAAGTGCAGTTAGAGTTCCAGTGCCACAATTGCAGCCGCAAA
ATCCATCTCAACAACAACCACAAGAGCAAGTTCCATTGATGCAACAACAACAACAATTTCCAGGGCAGCAAGAACAATTTCCACCACAACAGCC
ATATCCGCATCAGCAACCATTTCCATCACAACAACCATATCCGCAGCCGCAACCATTTCCGCCACAACTACCATATCCGCAGACGCAACCATTT
CCACCACAACAACCATATCCACAACCGCAACCACAGTATCCGCAACCACAACAACCAATTTCGCAGCAACAAGCACAACAACAACAACAACAAC
AACAAATCCTTCAACAAATTCTGCAACAACAACTGATTCCATGCAGGGATGTTGTCTTGCAACAACACAACATAGCGCATGCAAGCTCACAAGT
ATTGCAACAAAGTAGTTACCAACAGTTGCAACAATTATGTTGTCAGCAACTGTTTCAGATCCCCGAGCAGTCGCGGTGCCAAGCCATCCACAAT
GTCGTTCATGCTATTATTCTGCATCATCATCAACAACAACAACAACAACCGTCGAGCCAGGTCTCCTACCAGCAGCCTCAGGAACAATATCCAT
CAGGCCAGGGCTCCTTCCAGTCATCTCAGCAAAACCCACAGGCCCAGGGCTCTGTCCAGCCTCAACAACTGCCCCAGTTCCAGGAAATAAGGAA
CTTAGCGCTGCAGACGCTGCCAGCAATGTGCAATGTCTACATCCCTCCATATTGCTCGACCACCATTGCGCCATTTGGCATCTTCGGTACTAAC
TGA
>Gli-α19 deduced protein (282 aa)
MKTFLILALLAIVATTATSAVRVPVPQLQPQNPSQQQPQEQVPLMQQQQFPGQQEQFPPQQPYPHQQPFPSQQPYPQPQPFPPQLPYPQTQPF
PPQQPYPQPQPQYPQPQQPISQQQAQQQQQQQILQQILQQQLIPCRDVVLQQHNIAHASSQVLQQSSYQQLQQLCCQQLFQIPEQSRCQAIHN

VVHAIILHHHQQQQQQPSSQVSYQQPQEQYPSGQGSFQSSQQNPQAQGSVQPQQLPQFQEIRNLALQTLPAMCNVYIPPYCSTTIAPFGIFGTN
*

>*Gli-α20* transcript (861 bp)
ATGAAGACCTTTCTCATCCTTGCCCTCCTTGCTATTGTAGCAACCACCGCCACAATTGCAGTTAGAGTTCCAGTGCCACAATTGCAGCCACAAA
ATCCATCTCAGCAACAACCACAAGAGCAAGTTCCATTGGTACAACAACAGCAATTTCCAGGGCAGCAACAACCATTTCCACCACAACAACCATA
TCCGCAGCCGCAACCATTTCCATCACAACAACCATATCTGCAGCTGCAACCATTTCCGCAGCCGCAACTACCATATCCGCAGCCGCAACTACCA
TATCCGCAGCCGCAACCATTTCGACCACAACAACCATATCCACAACCGCAACCACAGTATTCGCAACCACAACAACCAATTTCGCAGCAGCAAC
AACAACAACAACAACAAATCCTTCAACAAATTTTGCAACAACAACTGATTCCATGCAGGGATGTTGTATTGCAACAACACAACATAGCGCATGG
AAGGTCACAAGTTTTGCAACAAAGTACTTACCAGCTGGTGCAACAATTGTGTTGTCAGCAGCTGTGGCAGATCCCCGAGCAGTCGCGGTGCCAA
GCCATCCACAATGTTGTTCATGCTATTATTCTGCATCATCATCAACAACAACAACAACAACAACAACCGTTGACCCAGGTCTCCTTCCAACAGC
CTCAACAACAATATCCATCAGGCCAGGGCTCCTTCCAGCCATCTCAGCAAAACCCACAGGCCCAGGGCTCTGTCCAGCCTCAACAACTGCCCCA
GTTTGAGGAAATAAGGAACCTAGCGCTAGAGACGCTACCTGCAATGTGCAATGTCTATATCCCTCCATATTGCACCATTGCTCAAGTTGGCATC
TTCGGTACTAACTGA
>Gli-α20 deduced protein (286 aa)
MKTFLILALLAIVATTATIAVRVPVPQLQPQNPSQQQPQEQVPLVQQQFPGQQQPFPPQQPYPQPQPFPSQQPYLQLQPFPQPQLPYPQPQLP
YPQPQPFRPQQPYPQPQPQYSQPQQPISQQQQQQQQILQQILQQQLIPCRDVVLQQHNIAHGRSQVLQQSTYQLVQQLCCQQLWQIPEQSRCQ
AIHNVVHAIILHHHQQQQQQQQPLTQVSFQQPQQQYPSGQGSFQPSQQNPQAQGSVQPQQLPQFEEIRNLALETLPAMCNVYIPPYCTIAQVGI
FGTN*

>*Gli-α21* transcript (882 bp)
ATGAAGACCTTTCTCATCCTTGCACTCCTTGCTATTGTAGCAACCACCGCCACAATTGCAGTTAGAGTTCCAGTGCCACAATTGCAGCCACAAA
ATCCATCTCAGCAACAACCACAAGAGCAAGTTCCATTGGTACAACAACAACAATTTCCAGGGCAGCAACAACCATTTCCACCACAACAGCCATA
TCCGCAGCCGCAACCATTTCCATCACAACAACCATATCTGCAGCTGCAGCCATTTCCGCAGCCGCAACTACCATATCCGCAGCCGCATCTACCA
TATCCGCAGCCGCAACCATTTCGACCACAACAACCATATCCACAACCGCAACCACAGTATTCGCAACCACAACAACCAATTTCGCAGCAGCAGC
AACAACAACAACAACAACAACAACAACAACAGATCCTTCAACAAATTTTGCAACAACAACTGATTCCATGCAGGGATGTTGTATTGCAACAACA
CAGCATAGCGCATGGAAGCTCACAAGTTTTGCAACAAAGTACTTACCAGCTGGTGCAACAATTGTGTTGTCAGCAGCTGTGGCAGATCCCCGAG
CAGTCGCGGTGCCAAGCCATCCACAATGTTGTTCATGCTATTATTCTGCATCAACAACAACAACAACAACAACAACAACAACAACAACCGTTGA
GCCAGGTCTCCTTCCAACAGCCTCAACAACAATATCCATCAGGCCAGGGCTCCTTCCAGCCATCTCAGCAAAACCCACAGGCCCAGGGCTCTGT
CCAGCCTCAACAACTGCCCCAGTTTGAGGAAATAAGGAACCTAGCGCTAGAGACGCTACCTGCAATGTGCAATGTCTATATCCCTCCATATTGC
ACCATTGCTCCAGTTGGCTTCTTCGGTACTAACTGA
>Gli-α21 deduced protein (293 aa)
MKTFLILALLAIVATTATIAVRVPVPQLQPQNPSQQQPQEQVPLVQQQFPGQQQPFPPQQPYPQPQPFPSQQPYLQLQPFPQPQLPYPQPHLP

	YPQPQPFRPQQPYPQPQPQYSQPQQPISQQQQQQQQQQQQQQILQQILQQQLIPCRDVVLQQHSIAHGSSQVLQQSTYQLVQQLCCQQLWQIPE<br>QSRCQAIHNVVHAIILHQQQQQQQQQQQQPLSQVSFQQPQQQYPSGQGSFQPSQQNPQAQGSVQPQQLPQFEEIRNLALETLPAMCNVYIPPYC<br>TIAPVGFFGTN*<br><br>>*Gli-α22* transcript (927 bp)<br>ATGAAGACCTTTCTCATCCTTGCCCTCCTTGCTATTGTAGCAACCACCGCCACAATTGCAGTTAGAGTTCCAGTGCCACAATTGCAGCCACAAA<br>ATCCATCTCAGCAACAACCACAAGAGCAAGTTTCATTGGTACAACAACAACAATTTCCAGGGCAGCAACAACCATTTCCACCACAACAGCCATA<br>TCCGCAGCCGCAACCATTTCCATCACAACAACCATATCTGCAGCTGCAACCATTTCCGCAGCCGCAACTACCATATCCGCAGCCGCAACTACCA<br>TATCCGCAGCCGCAACTACCATATCCGCAGCCGCAACCATTTCGACCACAACAACCATATCCACAATCGCAACCACAGTATTCGCAACCACAAC<br>AACCAATTTCGCAGCAGCAGCAGCAGCAACAACAACAACAACAACAAAAACAACAACAACAACAACAACAACAGATCCTTCAACAAATTTTGCA<br>ACAACAACTGATTCCATGCAGGGATGTTGTATTGCAACAACACAGCATAGCGTATGGAAGCTCACAAGTTTTGCAACAAAGTACTTACCAGCTG<br>GTGCAACAATTGTGTTGTCAGCAGCTGTGGCAGATCCCCGAGCAGTCGCGGTGCCAAGCCATCCACAATGTTGTTCATGCTATTATTCTGCATC<br>AACAGCAACAACAACAACAACAACAACAACAACAACAACCGTTGAGCCAGGTCTCCTTCCAACAGCCTCAACAACAATATCCATCAGGCCAGGG<br>CTCCTTCCAGCCATCTCAGCAAAACCCACAGGCCCAGGGCTCTGTCCAGCCTCAACAACTGCCCCAGTTTGAGGAAATAAGGAACCTAGCGCTA<br>GAGACGCTACCTGCAATGTGCAATGTCTATATCCCTCCATATTGCACCATTGCTCCAGTTGGCATCTTCGGTACTAACTGA<br>>Gli-α22 deduced protein (308 aa)<br>MKTFLILALLAIVATTATIAVRVPVPQLQPQNPSQQQPQEQVSLVQQQQFPGQQQPFPPQQPYPQPQPFPSQQPYLQLQPFPQPQLPYPQPQLP<br>YPQPQLPYPQPQPFRPQQPYPQSQPQYSQPQQPISQQQQQQQQQQQQKQQQQQQQQILQQILQQQLIPCRDVVLQQHSIAYGSSQVLQQSTYQL<br>VQQLCCQQLWQIPEQSRCQAIHNVVHAIILHQQQQQQQQQQQQQPLSQVSFQQPQQQYPSGQGSFQPSQQNPQAQGSVQPQQLPQFEEIRNLAL<br>ETLPAMCNVYIPPYCTIAPVGIFGTN*<br><br>>*Gli-α23* transcript (930 bp)<br>ATGAAGACCTTTCTCATCCTTGCCCTCCTTGCTATTGTAGCAACCACCGCCACAATTGCAGTTAGAGTTCCAGTGCCACAATTGCAGCCACAAA<br>ATCCATCTCAGCAACAACCACAAGAGCAAGTTCCATTGGTACAACAACAACAATTTCCAGGGCAGCAACAACCATTTCCACCACAACAGCCATA<br>TCCGCAGCCGCAACCATTTCCATCACAACAACCATATCTGCAGCTGCAACCATTTCCGCAGCCGCAACTACCATATCCGCAGCCGCAACTACCA<br>TATCCGCAGCCGCAACTACCATATCCGCAGCCGCAACCATTTCGACCACAACAACCATATCCACAATCGCAACCACAGTATTCGCAACCACAAC<br>AACCAATTTCGCAGCAGCAGCAGCAACAACAACAACAACAACAAAAACAACAACAACAACAACAACAACAACAGATCCTTCAACAAATTTTGCA<br>ACAACAACTGATTCCATGCAGGGATGTTGTATTGCAACAACACAGCATAGCGTATGGAAGCTCACAAGTTTTGCAACAAAGTACTTACCAGCTG<br>GTGCAACAATTGTGTTGTCAGCAGCTGTGGCAGATCCCCGAGCAGTCGCGGTGCCAAGCCATCCACAATGTTGTTCATGCTATTATTCTGCATC<br>AACAGCAACAACAACAACAACAACAACAACAACAAAAACAACCATTGAGCCAGGTCTCCTTCCAACAGCCTCAACAACAATATCCATCAGGCCA<br>GGGCTCCTTCCAGCCATCTCAGCAAAACCCACAGGCCCAGGGCTCTGTCCAGCCTCAACAACTGCCCCAGTTTGAGGAAATAAGGAACCTAGCG<br>CTAGAGACGCTACCTGCAATGTGCAATGTCTATATCCCTCCATATTGCACCATTGCTCCAGTTGGCATCTTCGGTACTAACTGA

>Gli-α23 deduced protein (309 aa)
MKTFLILALLAIVATTATIAVRVPVPQLQPQNPSQQQPQEQVPLVQQQQFPGQQQPFPPQQPYPQPQPFPSQQPYLQLQPFPQPQLPYPQPQLP
YPQPQLPYPQPQPFRPQQPYPQSQPQYSQPQQPISQQQQQQQQQQQKQQQQQQQQQILQQILQQQLIPCRDVVLQQHSIAYGSSQVLQQSTYQL
VQQLCCQQLWQIPEQSRCQAIHNVVHAIILHQQQQQQQQQQQQKQPLSQVSFQQPQQQYPSGQGSFQPSQQNPQAQGSVQPQQLPQFEEIRNLA
LETLPAMCNVYIPPYCTIAPVGIFGTN*

>*Gli-α24* transcript (876 bp)
ATGAAGACCTTTCTCATCCTTGCCCTCCTTGCTATTGTGGCGACCACCGCCACAACTGCAGTTAGAGTTCCAGTGCCACAATTGCAGCTACAAA
ATCCATCTCAGCAACAGCCACAAGAGCAAGTTCCATTGGTACAAGAACAACAATTTCCAGGGCAGCAACAACCATTTCCACCACAACAGCCATA
TCCGCAGCCGCAACCATTTCCATCACAACAACCATATCTGCAGCTGCAACCATTTCCACAGCCGCAACTACCATATCCGCAGCCGCAACCATTT
CGACCACAACAACCATATCCACAGCCGCAACCACAGTATTCGCAACCACAACAACCAATTTCGCAGCAGCAGCAGCAGCAGCAGCAACAACAAC
AACAACAACAACAAATCCTACAACAAATTTTGCAACAACAACTGATTCCATGCAGGGATGTTGTATTGCAACAACACAACATAGCGCATGGAAG
CTCACAAGTTTTGCAAGAAAGTACTTACCAGCTGGTGCAACAATTGTGTTGTCAGCAGCTGTGGCAGATCCCCGAGCAGTCGCGGTGCCAAGCC
ATCCACAATGTTGTTCATGCTATTATTCTGCATCAACAACACCACCACCACCAACAACAACAACAACAACAACAACAACAACCGTTGAGCCAAG
TCTCCTTCCAACAGCCTCAGCAACAATATCCATCAGGCCAGGGCTTCTTCCAACCATCTCAGCAAAACCCACAGGCCCAGGGCTCTTTCCAGCC
TCAACAACTGCCCCAGTTTGAGGCAATAAGGAACCTAGCGCTACAGACGCTACCTGCAATGTGCAATGTGTATATCCCTCCATATTGCACCATT
GCTCCATTTGGCATCTTCGGTACTAACTGA
>Gli-α24 deduced protein (291 aa)
MKTFLILALLAIVATTATTAVRVPVPQLQLQNPSQQQPQEQVPLVQEQQFPGQQQPFPPQQPYPQPQPFPSQQPYLQLQPFPQPQLPYPQPQPF
RPQQPYPQPQPQYSQPQQPISQQQQQQQQQQQQQQILQQILQQQLIPCRDVVLQQHNIAHGSSQVLQESTYQLVQQLCCQQLWQIPEQSRCQA
IHNVVHAIILHQQHHHHQQQQQQQQQQPLSQVSFQQPQQQYPSGQGFFQPSQQNPQAQGSFQPQQLPQFEAIRNLALQTLPAMCNVYIPPYCTI
APFGIFGTN*

>*Gli-α25* transcript (900 bp)
ATGAAGACCTTTCTCATCCTTGCCCTCCTTGCTATTGTAGCAACCACCGCCACAATTGCAGTTAGAGTTCCAGTGCCACAATTGCAGCCACAAA
ATCCATCTCAGCAACAACCACAAGAGCAAGTTCCATTGGTACAACAACAACAATTTCCAGGGCAGCAACAACCATTTCCACCACAACAGCCATA
TCCGCAGCTGCAACCATTTCCATCACAACAACCATATATGCAGCTGCAACCATTTCCGCAGCCGCAACTACCATATCCGCAGCCGCAACTACCA
TATCCGCAGCCGCAACCATTTCGACCACAACAATCATATCCACAACCGCAACCACAGTATTCGCAACCACAACAACCAATTTCGCAGCAGCAGC
AGCAGCAGCAGCAACAACAACAACAACAGATCCTTCAACAAATTTTGCAACAACAACTGATTCCATGCAGGGATGTTGTATTGCAACAACACAG
CATAGCGCATGGAAGCTCACAAGTTTTGCAACAAAGTACTTACCAGCTGGTGCAACAATTGTGTTGTCAGCAGCTGTGGCAGATCCCCGAGCAG
TCGCGGTGCCAAGCCATCCACAATGTTGTTCATGCTATTATTCTGCATCAACAACAACAACAACAACAACAACAACAACAACAACAACAACAAC
AACAACAACAACCGTTGAGCCAGGTCTGCTTCCAACAGTCTCAACAACAATATCCATCAGGCCAGGGCTCCTTCCAGCCATCTCAGCAAAACCC
ACAGGCCCAGGGCTCTGTCCAGCCTCAACAACTGCCCCAGTTTGAGGAAATAAGGAACCTAGCGCTAGAGACGCTACCTGCAATGTGCAATGTC

TATATCCCTCCATATTGCACCATTGCTCCAGTTGGCATCTTCGGTACTAACTGA
>Gli-α25 deduced protein (299 aa)
MKTFLILALLAIVATTATIAVRVPVPQLQPQNPSQQQPQEQVPLVQQQQFPGQQQPFPPQQPYPQLQPFPSQQPYMQLQPFPQPQLPYPQPQLP
YPQPQPFRPQQSYPQPQPQYSQPQQPISQQQQQQQQQQQQQILQQILQQQLIPCRDVVLQQHSIAHGSSQVLQQSTYQLVQQLCCQQLWQIPEQ
SRCQAIHNVVHAIILHQQQQQQQQQQQQQQQQQQQQPLSQVCFQQSQQQYPSGQGSFQPSQQNPQAQGSVQPQQLPQFEEIRNLALETLPAMCNV
YIPPYCTIAPVGIFGTN*

2) Transcript and deduced protein sequences for 11 transcribed and intact γ-gliadin genes (each transcript sequence is provided from start to stop codons)

> *Gli-γ1* transcript (1020 bp)
ATGAAGACCTTACTCATCCTGACAATCATTGCGGTGGCACTAACTACCACCACCGCCAATATACAGGTCGACCCTAGTGGCCAAGTACAATGGC
CACAACAACAACAACCATTCCCCCAGCCCCAACAACCATTCTCCCAACAACCACAACAAATTTTTCCCCAACCCCAACAAACATTCCCCCATCA
ACCACAACAAGCATTTCCCCAACCCCAACAAACATTCCCCCATCAACCACAACAACAATTTCCCCAGCCCCAGCAACCACAACAACCATTTCCC
CAGCAACCACAACAACAATTTCCCCAGCCCCAACAACCACAACAACCATTTCCCCAGCAACCACAACAACAATTTCCCCAGCCCCAACAACCAC
AACAACCATTTCCCCAGCCCCAACAACCCCAACTACCATTTCCGCAACAACCACAACAACCATTCCCCCAGCCTCAACAACCCCAACAACCATT
TCCCCAGTTACAGCAACCACAACAACCTTTACCCCAGCCCCAACAACCGCAACAACCATTCCCCCAGCAACAACAACCATTGATTCAGCCATAC
CTACAACAACAGATGAACCCCTGCAAGAATTACCTCTTGCAGCAATGCAACCCTGTGTCATTGGTGTCATCCCTCGTGTCAATGATCTTGCCAC
GAAGTGATTGCAAGGTGATGCGGCAACAATGTTGCCAACAACTAGCACAGATTCCTCAGCAGCTCCAGTGCGCAGCCATCCATGGCATCGTGCA
TTCCATCATCATGCAGCAAGAACAACAACAACAACAACAACAACAACAACAACAACAACAACAACAACAAGGCATACAGATCATGCGGCCACTATTT
CAGCTCGTCCAGGGTCAGGGCATCATCCAACCTCAACAACCAGCTCAATTGGAGGTGATCAGGTCATTGGTATTGGGAACTCTTCCAACCATGT
GCAACGTGTTTGTTCCACCTGAGTGCTCCACCACCAAGGCACCATTTGCCAGCATAGTCGCCGACATTGGTGGCCAATGA
> Gli-γ1 deduced protein (339 aa)
MKTLLILTIIAVALTTTTANIQVDPSGQVQWPQQQPFPQPQQPFSQQPQQIFPQPQQTFPHQPQQAFPQPQQTFPHQPQQQFPQPQQPQQPFP
QQPQQQFPQPQQPQQPFPQQPQQQFPQPQQPQQPFPQPQQPQLPFPQQPQQPFPQPQQPQQPFPQLQQPQQPLPQPQQPQQPFPQQQQPLIQPY
LQQQMNPCKNYLLQQCNPVSLVSSLVSMILPRSDCKVMRQQCCQQLAQIPQQLQCAAIHGIVHSIIMQQEQQQQQQQQQQQQQQGIQIMRPLF
QLVQGQGIIQPQQPAQLEVIRSLVLGTLPTMCNVFVPPECSTTKAPFASIVADIGGQ*

> *Gli-γ2* transcript (858 bp)
ATGAAGACCTTATTCATCCTAACAATCCTTGCGATGGCAACAACTATCGCCACCGCGAATATGCAGGTCGACCCCAGCGGCCAAGTACAATGGC
CACAACAACAACCATTCCGCCAGCCCCAACAACCATTCTACCAGCAACCACAACACACATTTCCCCAACCCCAACAAACATTCCCCCATCAACC

ACAACAACAATTTCCCCAGCCCCAGCAACCACAACAACAATTTCCGCAGCCCCAGCAACCACAACAACCATTTCCCCAGCCCCAACAAGCCCAA
CTACCATTTCCCCAACAACCACAACAACCATTCCCCCAGCCTCAACAACCCCAACAACCATTTCCCCAGTCACAGCAACCACAACAACCTTTTC
CCCAGCCCCAACAACCGCAACAATCATTCCCCCAGCAACAACAACCGTTGATTCAGCCATATCTACAACAACAGATGAACCCTTGCAAGAATTA
CCTCTTACAACAATGCAACCCTGTGTCATTGGTGTCATCCCTCGTGTCAATGATATTGCCACGAAGTGATTGCCAGGTGATGCAGCAACAATGT
TGCCAACAACTAGCACAGATTCCTCGCCAGCTCCAGTGTGCAGCCATCCATAGCGTCGTGCATTCCATCGTCATGCAGCAAGAACAACAACAAG
GCATACAGATCCTCCGGCCACTGTTTCAGCTCGTCCAAGGTCAGGGCATCATCCAACCTCAACAACCAGCTCAATATGAGGTGATCAGGTCATT
GGTATTGAGAACCCTTCCAAACATGTGCAACGTGTATGTCCGACCTGACTGCTCCACCATCAACGCACCATTTGCCAGCATAGTCGCCGGCATC
AGTGGACAATGA

> Gli-γ2 deduced protein (285 aa)

MKTLFILTILAMATTIATANMQVDPSGQVQWPQQQPFRQPQQPFYQQPQHTFPQPQQTFPHQPQQQFPQPQQPQQQFPQPQQPQQPFPQPQQAQ
LPFPQQPQQPFPQPQQPQQPFPQSQQPQQPFPQPQQPQQSFPQQQQPLIQPYLQQQMNPCKNYLLQQCNPVSLVSSLVSMILPRSDCQVMQQQC
CQQLAQIPRQLQCAAIHSVVHSIVMQQEQQQGIQILRPLFQLVQGQGIIQPQQPAQYEVIRSLVLRTLPNMCNVYVRPDCSTINAPFASIVAGI
SGQ*

> *Gli-γ3* transcript (858 bp)

ATGAAGACCTTATTCATCCTAACAATCCTTGCGATGGCAACAACTATCGCCACCGCGAATATGCAGGTCGACCCCAGCGGCCAAGTACAATGGC
CACAACAACAACCATTCCGCCAGCCCCAACAACCATTCTACCAGCAACCACAACACACATTTCCCCAACCCCAACAAACATTCCCCCATCAACC
ACAACAACAATTTCCCCAGCCCCAGCAACCACAACAACAATTTCCGCAGCCCCAGCAACCACAACAACCATTTCCCCAGCCCCAACAAGCCCAA
CTACCATTTCCCCAACAACCACAACAACCATTCCCCCAGCCTCAACAACCCCAACAACCATTTCCCCAGTCACAGCAACCACAACAACCTTTTC
CCCAGCCCCAACAACCGCAACAATCATTCCCCCAGCAACAACAACCGTTGATTCAGCCATATCTACAACAACAGATGAACCCTTGCAAGAATTA
CCTCTTACAGCAATGCAACCCTGTGTCATTGGTGTCATCCCTCGTGTCAATGATCTTGCCACGAAGTGATTGCCAGGTGATGCAGCAACAATGT
TGCCAACAACTAGCACAGATTCCTCGCCAGCTCCAGTGTGCAGCCATCCATAGCGTCGTGCATTCCATCGTCATGCAGCAAGAACAACAACAAG
GCATACAGATCCTCCGGCCACTGTTTCAGCTCGTCCAAGGTCAGGGCATCATCCAACCTCAACAACCAGCTCAATATGAGGTGATCAGGTCATT
GGTATTGAGAACCCTTCCAAACATGTGCAACGTGTATGTCCGACCTGACTGCTCCACCATCAACGCACCATTTGCCAGCATAGTCGCCGGCATC
AGTGGACAATGA

> Gli-γ3 deduced protein (285 aa)

MKTLFILTILAMATTIATANMQVDPSGQVQWPQQQPFRQPQQPFYQQPQHTFPQPQQTFPHQPQQQFPQPQQPQQQFPQPQQPQQPFPQPQQAQ
LPFPQQPQQPFPQPQQPQQPFPQSQQPQQPFPQPQQPQQSFPQQQQPLIQPYLQQQMNPCKNYLLQQCNPVSLVSSLVSMILPRSDCQVMQQQC
CQQLAQIPRQLQCAAIHSVVHSIVMQQEQQQGIQILRPLFQLVQGQGIIQPQQPAQYEVIRSLVLRTLPNMCNVYVRPDCSTINAPFASIVAGI
SGQ*

	> *Gli-γ4* transcript (1074 bp)<br>ATGAAGACCTTACTCATCCTGACGATCCTTGCGATGGCAATAACCATCAGCACCGCCAATATGCAGGTCGACCCTAGTGGCCAAGTACAATGGC<br>CACAACAACAACTAGTCCCCCAACCCCAACAGCCATTATCCCAGCAACCGCAACAAGCATTTCCCCAACCCCAACAAACATTTCCCCATCAACC<br>ACAACAACAAGTTCCCCAGCCTCAGCAACCACAACAACCATTTCTCCAGCCCCAACAAGCATTCCCCCAACAACCACAACAACCATTCCCTCAG<br>ACTCAACAACCACAACAACCATTTCCCCAGCAACCACAACAACCATTCCCCCAGACTCAACAACCACAACAACCATTTCCCCAGCAACCACAAC<br>AACCATTTCCCCAGCAACCACAACAACCATTCCCCCAGACTCAACAACCACAACAACCATTTCCCCAGCAACCACAACAACCATTCCCCCAGAC<br>TCAACAACCACAACAACCATTTCCCCAGTTCCAGCAACCACACCAACCTTTTCCCCAGCCCCAACAACAATTCCCGCAGCCCCAACAACCGCAA<br>CAATCATTCCTCCAGCAACAACGACCGTTCATTCAGCCATCTCTACAACAACGTTTGAACCCATGCAAGAATATCCTCTTGCAACAATGCAAAC<br>CTGCGTCATTGGTGTCATCCCTCTGGTCGATAATCTGGCCACAAAGCGATTGCCAAGTGATGCAGCAACAATGCTGCCAAGAACTAGCACAGAT<br>TCCTCAGCAGCTCCAGTGCGCAGCCATCCATAGCGTCGTGCATTCCATCATCATGCAGCAGCAACAACAACAACAACAACAACAACAACAACAA<br>CAAGGCATGCATATCCTGCTGACACTATCTCAACAACAACAGTTGGGTCAAGGTACTCTCGTCCAAGGCCAGGGCATCATCCAACCTCAACAAC<br>TAGCTCAATTGGAGGCGATCAGGTCATTGGTGTTGCAAACTCTTCCAACCATGTGCAACGTGTATGTCCCACCTGAGTGCTCCATCATCAGGGC<br>ACCATTTGCCAGCATAGTCGCGGGGATTGGTGGCCAATGA<br>> Gli-γ4 deduced protein (357 aa)<br>MKTLLILTILAMAITISTANMQVDPSGQVQWPQQQLVPQPQQPLSQQPQQAFPQPQQTFPHQPQQQVPQPQQPQQPFLQPQQAFPQQPQQPFPQ<br>TQQPQQPFPQQPQQPFPQTQQPQQPFPQQPQQPFPQQPQQPFPQTQQPQQPFPQQPQQPFPQTQQPQQPFPQFQQPHQPFPQPQQQFPQPQQPQ<br>QSFLQQQRPFIQPSLQQRLNPCKNILLQQCKPASLVSSLWSIIWPQSDCQVMQQQCCQELAQIPQQLQCAAIHSVVHSIIMQQQQQQQQQQQQQ<br>QGMHILLTLSQQQQLGQGTLVQGQGIIQPQQLAQLEAIRSLVLQTLPTMCNVYVPPECSIIRAPFASIVAGIGGQ*<br><br>> *Gli-γ5* transcript (909 bp)<br>ATGAAGACCTTACTCATCCTAACAATCCTTGCGATGGCAACAACCATCGCCACCGCCAATATGCAAGTCGACCCCAGCGGCCAAGTACAATGGC<br>CACAACAACAACCATTCCCCCAGCCCCAACAACCATTCTGCGAGCAACCACAACGAACTATTCCCCAACCCCATCAAACATTCCACCATCAACC<br>ACAACAAACATTTCCCCAACCCGAACAAACATACCCCCATCAACCACAACAACAATTTCCCCAGACCCAACAACCACAACAACCATTTCCCCAG<br>CCCCAACAAACATTCCCCCAACAACCCCAACTACCATTTCCCCAACAACCCCAACAACCATTCCCCCAGCCTCAGCAACCCCAACAACCATTTC<br>CCCAGTCACAACAACCACAACAACCTTTTCCCCAGCCCCAACAACAATTTCCGCAGCCCCAACAACCACAACAATCATTCCCCCAACAACAACA<br>ACCGGCGATTCAGTCATTTCTACAACAACAGATGAACCCCTGCAAGAATTTCCTCTTGCAGCAATGCAACCATGTGTCATTGGTGTCATCTCTC<br>GTGTCAATAATTTTGCCACGAAGTGATTGCCAGGTGATGCAGCAACAATGTTGCCAACAACTAGCACAAATTCCTCAACAGCTCCAGTGCGCAG<br>CCATCCACAGCGTCGCGCATTCCATCATCATGCAACAAGAACAACAACAAGGCGTGCCGATCCTGCGGCCACTATTTCAGCTCGCCCAGGGTCT<br>GGGTATCATCCAACCTCAACAACCAGCTCAATTGGAGGGGATCAGGTCATTGGTATTGAAAACTCTTCCAACCATGTGCAACGTGTATGTGCCA<br>CCTGACTGCTCCACCATCAACGTACCATATGCCAACATAGACGCTGGCATTGGTGGCCAATGA<br>> Gli-γ5 deduced protein (302 aa)<br>MKTLLILTILAMATTIATANMQVDPSGQVQWPQQQPFPQPQQPFCEQPQRTIPQPHQTFHHQPQQTFPQPEQTYPHQPQQQFPQTQQPQQPFPQ

	PQQTFPQQPQLPFPQQPQQPFPQPQQPQQPFPQSQQPQQPFPQPQQQFPQPQQPQQSFPQQQQPAIQSFLQQQMNPCKNFLLQQCNHVSLVSSL<br>VSIILPRSDCQVMQQQCCQQLAQIPQQLQCAAIHSVAHSIIMQQEQQQGVPILRPLFQLAQGLGIIQPQQPAQLEGIRSLVLKTLPTMCNVYVP<br>PDCSTINVPYANIDAGIGGQ*<br><br>> *Gli-γ6* transcript (894 bp)<br>ATGAAGACCTTACTCATCCTAACAATCCTTGCGATGGCAACAATAATCGCCACTGCCAATATGCAGGTCGACCCTAGCAGCCGAGTACAATGGC<br>CACAAGAACAACCATCCCCCCAGTCCCAACAACCATTCTCCCAGCAACCACAACAAATATTTCCCCAACCCCAACAAACATTGCCCCATCAACC<br>ACAACAAGCATTTCCCCAACCTCAACAAACATTCCCCCATCGACCACAACAACAATTTCCCCAGCCCCAGCAACCACAACAACCATTTCCTCAG<br>CCCCAACAACCCCAACTACCATTTCCCCAACAACCACAACAACCATTCCCCCAGCCTCAACAACCCCAACAACCATTTCCCCAGTCACAGCAAC<br>CACAACAACCTTTTCCCCAGCCCCAACAACAATTTCCGCAGCCCCAACAACCACAACAATCATTCCCCCAACAACAACAATGGATGATTCAGTC<br>ATTTCTACAACAACAGATGAACCCCTGCAAGAATTTCCTCTTGCAGCAATGCAACCCTGTGTCATTGGTGTCATCTCTCGTGTCAATAATCTTG<br>CCACGAAGTGATTGCCAGCTGATGCAGCAACAATGTTGCCAACAACTAGCACAAATTCCTCAACAGCTCCAGTGCGCAGCCATCCACAGCGTCG<br>CGCATTCCATCGTCATGCAGCAAGAACAACAACGAGGCGTGCAGATCCTGCGGCCACTATTTCAGCTCGCCCAGGGTCTGGGTATCATCCAACC<br>TCAACAACCAGCTCAATTGGAGGGGATCAGGTCATTGGTATTGAAAACTCTTCCAACCATGTGCAATGTGTATGTCCCACCTGACTGCTCCACC<br>ATCAACGTGCCATATGCCAGCATAGACGCTGTCATTGGTGGCCAATGA<br>> Gli-γ6 deduced protein (297 aa)<br>MKTLLILTILAMATIIATANMQVDPSSRVQWPQEQPSPQSQQPFSQQPQQIFPQPQQTLPHQPQQAFPQPQQTFPHRPQQQFPQPQQPQQPFPQ<br>PQQPQLPFPQQPQQPFPQPQQPQQPFPQSQQPQQPFPQPQQQFPQPQQPQQSFPQQQQWMIQSFLQQQMNPCKNFLLQQCNPVSLVSSLVSIIL<br>PRSDCQLMQQQCCQQLAQIPQQLQCAAIHSVAHSIVMQQEQQRGVQILRPLFQLAQGLGIIQPQQPAQLEGIRSLVLKTLPTMCNVYVPPDCST<br>INVPYASIDAVIGGQ*<br><br>> *Gli-γ7* transcript (876 bp)<br>ATGAAGACCTTACTCATCCTGACAATCCTTGCGATGGCAATAACCATCGCCACCGCCAATATGCAGGTCGACCCTAGCGGCCAAGTACAATGGC<br>CGCAACAACAACCATTCCTGCAGCCTCACCAACCATTCTCCCAGCAACCACAACAAATATTTCCCCAACCCCAACAAACATTCCCCCATCAACC<br>ACAACAACAATTTCCCCAGCCTCAGCAACCACAACAACAATTTCTCCAGCCCCGACAACCATTCCCCCAACAACCACAACAACCATATCCCCAG<br>CAACCACAACAACCGTTCCCCCAGACTCAACAACCCCAACAACCATTTCCCCAGTCCAAGCAACCACAACAACCTTTTCCCCAGCCCCAACAAC<br>CGCAACAATCATTCCCCCAACAACAACCATCATTGATTCAACAATCTCTACAACAACAGTTGAACCCATGCAAGAATTTCCTCTTGCAGCAATG<br>CAAACCTGTGTCCTTGGTGTCATCCCTCTGGTCAATCATCTTGCCACCAAGCGATTGCCAGGTGATGCGGCAACAATGTTGTCAACAACTAGCA<br>CAAATTCCTCAGCAACTCCAGTGTGCAGCCATCCATAGCGTCGTGCATTCCATCATCATGCAGCAAGAACAACAAGAACAACTACAGGGTGTGC<br>AAATCCTGGTGCCACTGTCTCAACAGCAACAGGTGGGTCAAGGTATTCTCGTCCAGGGTCAAGGCATCATCCAACCTCAACAACCAGCTCAATT<br>GGAGGTGATCAGGTCATTGGTGTTGCAAACTCTTCCAACCATGTGCAACGTGTATGTCCCACCTTACTGCTCCACCATCAGGGCACCATTTGCT<br>AGCATAGTCGCCAGCATTGGTGGCCAATGA

> Gli-γ7 deduced protein (291 aa)
MKTLLILTILAMAITIATANMQVDPSGQVQWPQQQPFLQPHQPFSQQPQQIFPQPQQTFPHQPQQQFPQPQQPQQQFLQPRQPFPQQPQQPYPQ
QPQQPFPQTQQPQQPFPQSKQPQQPFPQPQQPQQSFPQQQPSLIQQSLQQQLNPCKNFLLQQCKPVSLVSSLWSIILPPSDCQVMRQQCCQQLA
QIPQQLQCAAIHSVVHSIIMQQEQQEQLQGVQILVPLSQQQQVGQGILVQGQGIIQPQQPAQLEVIRSLVLQTLPTMCNVYVPPYCSTIRAPFA
SIVASIGGQ*

> *Gli-γ8* transcript (984 bp)
ATGAAGACCTTACTCATCCTGACAATCCTTGCGATGGCAATAACCATCGGCACCGCCAATATCCAGGTCGACCCTAGCGGCCAAGTACAATGGC
TACAACAACAACTAGTCCCCCAGCTCCAACAGCCATTATCCCAGCAACCACAACAAACATTTCCCCAACCTCAACAAACATTCCCCCATCAACC
ACAACAACAAGTTCCCCAGCCTCAGCAACCACAACAACCATTTCTCCAGCCCCAACAACCATTCCCCCAACAACCACAACAACCATTCCCCCAG
ACTCAACAACCACAACAACCATTTCCCCAGCAACCACAACAACCATTTCCCCAGACTCAACAACCCCAACAACCATTTCCCCAACAACCACAAC
AACCATTCCCCCAGACTCAACAACCCCAACAACCATTTCCCCAGCTCCAGCAACCACAACAACCTTTTCCCCAGCCCCAACAACAATTGCCGCA
GCCCCAACAACCGCAACAATCATTCCCCCAACAACAACGGCCATTCATTCAACCATCTCTACAACAACAGTTGAACCCATGCAAGAATATCCTC
TTGCAACAATGCAAACCTGCGTCATTGGTGTCATCCCTCTGGTCAATAATCTGGCCACAAAGCGATTGCCAAGTGATGCGGCAACAATGCTGCC
AACAACTAGCACAGATTCCTCAACAGCTCCAGTGCGCAGCCATCCATAGCGTCGTGCATTCCATCATCATGCAGCAGCAGCAGCAACAACAACA
ACAACAAGGCATGCATATCTTTCTGCCACTATCTCAGCAGCAACAGGTGGGTCAAGGTTCTCTAGTCCAAGGCCAGGGCATCATCCAACCACAA
CAACCAGCTCAATTGGAGGCGATCAGATCATTGGTGTTGCAAACTCTTCCATCCATGTGCAACGTGTATGTCCCACCTGAGTGCTCCATCATGA
GGGCACCATTTGCCAGCATAGTCGCGGGCATTGGTGGCCAATGA

> Gli-γ8 deduced protein (327 aa)
MKTLLILTILAMAITIGTANIQVDPSGQVQWLQQQLVPQLQQPLSQQPQQTFPQPQQTFPHQPQQQVPQPQQPQQPFLQPQQPFPQQPQQPFPQ
TQQPQQPFPQQPQQPFPQTQQPQQPFPQQPQQPFPQTQQPQQPFPQLQQPQQPFPQPQQQLPQPQQPQQSFPQQQRPFIQPSLQQQLNPCKNIL
LQQCKPASLVSSLWSIIWPQSDCQVMRQQCCQQLAQIPQQLQCAAIHSVVHSIIMQQQQQQQQQQGMHIFLPLSQQQQVGQGSLVQGQGIIQPQ
QPAQLEAIRSLVLQTLPSMCNVYVPPECSIMRAPFASIVAGIGGQ*

> *Gli-γ9* transcript (897 bp)
ATGAAGACCTTACTCATCCTGACAATCCTTGCGATGGCAATAACCATCGGCACCGCCAATATGCAGGTCGACCCTAGCAGCCAAGTACAATGGC
CACAACAACAACCAGTCCCACAGCCTCACCAACCATTCTCCCAGCAACCACAACAAACATTTCCCCAACCCCAACAAACATTCCCCCATCAACC
ACAACAACAATTTCCCCAGCCTCAGCAACCACAACAACAATTTCTCCAGCCCCAACAACCATTCCCCCAACAACCACAACAACCATATCCCCAG
CAACCACAACAACCATTCCCCCAGACTCAACAACCCCAACAACTATTTCCCCAGTCCCAGCAACCACAACAACAATTTTCTCAGCCCCAACAAC
AATTCCCGCAGCCCCAACAACCGCAACAATCATTCCCCCAACAACAACCACCGTTCATTCAGCCATCTCTACAACAACAGGTGAACCCATGCAA
GAATTTCCTCTTGCAGCAATGCAAACCTGTGTCACTGGTGTCATCCCTCTGGTCAATGATCTGGCCACAAAGCGATTGCCAAGTGATGCGGCAA
CAATGCTGCCAACAACTAGCACAGATTCCTCAGCAGCTCCAGTGTGCAGCCATCCATACCATCATACATTCCATCATCATGCAGCAAGAACAAC

AAGAACAACAACAAGGCATGCATATCCTGCTGCCACTATATCAGCAGCAACAGGTGGGTCAAGGTACTCTCGTCCAGGGCCAGGGCATCATCCA
ACCCCAACAACCAGCTCAATTGGAGGCGATCAGGTCATTGGTGTTGCAAACTCTTCCAACCATGTGCAACGTGTATGTCCCACCTGAGTGCTCC
ATCATCAAGGCACCATTTTCCAGCGTAGTCGCCGGCATTGGTGGCCAATGA

> Gli-γ9 deduced protein (298 aa)
MKTLLILTILAMAITIGTANMQVDPSSQVQWPQQQPVPQPHQPFSQQPQQTFPQPQQTFPHQPQQQFPQPQQPQQQFLQPQQPFPQQPQQPYPQ
QPQQPFPQTQQPQQLFPQSQQPQQQFSQPQQQFPQPQQPQQSFPQQQPPFIQPSLQQQVNPCKNFLLQQCKPVSLVSSLWSMIWPQSDCQVMRQ
QCCQQLAQIPQQLQCAAIHTIIHSIIMQQEQQEQQQGMHILLPLYQQQQVGQGTLVQGQGIIQPQQPAQLEAIRSLVLQTLPTMCNVYVPPECS
IIKAPFSSVVAGIGGQ*

> *Gli-γ10* transcript (888 bp)
ATGAAGACCTTACTCATCGTAACAATCCTTGCGATGGCAACAACCATCGCCACCGCCAATATGCAAGTCGACCCCGGCTACCAAGTACAATGGC
CACAACAACAACCATTCCCCCAGCCCCAACAACCATTCTGCCAGCAACCACAACAAACTATTCCCCAACCCCATCAAACGTTCCACCATCAACC
ACAGCAAACATACCCCCATCAACCACAACAACAATTTCCCCAGACCCAGCAACCACAACAACCATTTCCCCAGCCCCAACAAACATTCCCCCAA
CAACCCCAACTACCATTTCCCCAACAACCCCAACAACCATTCCCCCAGCCTCAACAACCCCAACAACAATTTCCCCAGTCACAGCAACCACAAC
AACCTTTTCCCCAGCCCCAACAACAATTCCTGCAGCCCCAACAACCGCAACAATCATTCCCCCAGCAACAACAACCGTTGATTCAGCTATCTCT
ACAACAACAGATGAACCCCTGCAAGAATTTTCTCTTGCAGCAATGCAACCCTGTGTCATTGGTGTCATCCCTCATATCAATGATCTTGCCACGA
AGTGATTGCCAGGTGATGCAGCAACAATGTTGCCAACAACTGGCACAGATTCCTCAGCAGCTCCAGTGTGCAGCCATCCATAGTGTCGTGCATT
CCATCATCATGCAGCAAGAACAACGACAAGGCGTGCAGATCCGGCGGCCACTGTTTCAGCTCGTTCAGGGTCAGGGCATCATCCAACCTCAACA
ACCAGCTCAATTGGAGGTGATCAGGTCATTGGTATTGAGAACTCTTCCAACCATGTGCAACGTGTATGTCTCACCTGACTGCTCCACCATCAAC
GCACCATTTGCCAACATAGTCGTCGGCATTGGTGGCCAATGA

> Gli-γ10 deduced protein (295 aa)
MKTLLIVTILAMATTIATANMQVDPGYQVQWPQQQPFPQPQQPFCQQPQQTIPQPHQTFHHQPQQTYPHQPQQQFPQTQQPQQPFPQPQQTFPQ
QPQLPFPQQPQQPFPQPQQPQQQFPQSQQPQQPFPQPQQQFLQPQQPQQSFPQQQQPLIQLSLQQQMNPCKNFLLQQCNPVSLVSSLISMILPR
SDCQVMQQQCCQQLAQIPQQLQCAAIHSVVHSIIMQQEQRQGVQIRRPLFQLVQGQGIIQPQQPAQLEVIRSLVLRTLPTMCNVYVSPDCSTIN
APFANIVVGIGGQ*

> *Gli-γ11* transcript (897 bp)
ATGAAGACCTTACTCATCCAAACAATCCTCGTGATGGCAATAACCATCGCCACCGCCAATATGCAGGTCGACCCTAGCGGCCAAGTACCATGGC
CACAACAACAACCATTCCCGCAGCCTCACCAACCATTCTCCCAGCAACCGCAACAAACATTTCCCCAACCCCAACAAACATTCCCCCATCAACC
ACAACAACAATTTTCCCAGCCTCAGCAACCACAACAACAATTTATCCAGCCCCAACAACCATTCCCCCAACAACCACAACAAACATATCCCCAG
CAACCACAACAACCATTCCCCCAGACTCAACAACCCCAACAACTATTTCCCCAGTCCCAGCAACCACAACAACAATTTCCCCAGCCCCAACAAC
AATTCCCGCAGCCCCAACAACCACAACAATCATTCCCCCAACAACAACCATCGTTGATTCAACAATCTCTACAACAACAGTTGAACCCATGCAA

GAATTTCCTCTTGCAACAATGCAAACCTGTGTCCTTGGTGTCATCCCTCTGGTCAATGATCTTGCCACGAAGCGATTGCCAGGTGATGCGGCAA
CAATGTTGCCAACAACTAGCACAAATTCCTCAGCAACTCCAGTGTGCAGCCATCCATAGCATCGTGCATTCCATCATCATGCAGCAAGAACAAC
AAGAACAACGACAGGGTGTGCAAATCCTGGTGCCACTGTCTCAACAGCAACAGGTAGGTCAAGGTACTCTCGTCCAAGGTCAGGGCATCATCCA
ACCTCAACAACCAGCTCAATTGGAGGTGATTAGGTCATTGGTGTTGCAAACTCTTGCAACCATGTGCAACGTGTATGTCCCACCTTACTGCTCC
ACCATCAGGGCACCATTTGCCAGCATAGTCGCCGGCATTGGTGGCCAATGA

> Gli-γ11 deduced protein (298)

MKTLLIQTILVMAITIATANMQVDPSGQVPWPQQQPFPQPHQPFSQQPQQTFPQPQQTFPHQPQQQFSQPQQPQQQFIQPQQPFPQQPQQTYPQ
QPQQPFPQTQQPQQLFPQSQQPQQQFPQPQQQFPQPQQPQQSFPQQQPSLIQQSLQQQLNPCKNFLLQQCKPVSLVSSLWSMILPRSDCQVMRQ
QCCQQLAQIPQQLQCAAIHSIVHSIIMQQEQQEQRQGVQILVPLSQQQQVGQGTLVQGQGIIQPQQPAQLEVIRSLVLQTLATMCNVYVPPYCS
TIRAPFASIVAGIGGQ*

3) Transcript and deduced protein sequences for the single transcribed and intact δ-gliadin gene (the transcript sequence is provided from start to stop codons)

> *Gli-δ1* transcript (984 bp)

ATGAAGATCTTCATGGTCTTTGCCCTCCTCGTTGCATCAACGACCATCACCACCGCGACCGCACAGCTCGACCCTCGCATCCATGACCAAGAAA
GGCCACAACAATCGTTTCTGCAACAGCAACCACTTATCCAGCAACAACCATACCCGCCTCAAGAGCCACAACAACCACTATTCCCGCAAAAAGA
GCCACAACAACCATTTCCGCTGCAGCAGCCACAATACCAGCAACAACAACCGTATCCACAACAACCACTTCCCCAAGAACAACTTCCCCAGCAA
CATTTATTTCCGCAGCAGCCGCCACAACAACAATTTCCACAACAGATGCCACTTCCGCATCAACAACAAACATTCCCGCAACAACAACAACAAC
AACAACAACAAGAACAACTCCCACAACAACTCCCACAATTCCCGCAACAACAACCATTTTCCCAATATCAACAACCATTAACACAACAACCATA
CTCGCAAGAGCAACCATTGCCACAACAACAACCTTCTGTAGAGGAAAAACAACAATTGAACTTGTGCAAGGAGTTCCTCCTGCAGCAGTGTAAC
CCAGAGGAGAAACTGTCGTTACTCCAGTCAGTGATCCCGTTCCTCCGACCAAAGACCTCGCAACAGAACAGCTGCCAGTTGAAGCGACTACAAT
GTTGTCGACAACTTGCACATATCAGTGAACCGTCCCGATGCCCGGCCATCCACAACATTGTGCATGCCATCGTCATGCAACAACAACATGTGGA
TAGAGGTTTCGGCCAGCCTCAACCACAACAGTTGGGCCAGGAAATGCCCATGCAGCCTCAACATCAATTGGGCCAGCACTCTATCCTACCTCAA
CAACTAGCCCAGTACAAGTTGGTTAGGTTACTTGTGATTCAGACCCTTCCTATGTTATGCAACGTGCATGTCCCGTCTGATTGCTACACCATTA
CTGCACCATTTGGTAGCATGACTGCCTACAATGGTGGACAATGA

> Gli-δ1 deduced protein (327 aa)

MKIFMVFALLVASTTITTATAQLDPRIHDQERPQQSFLQQQPLIQQQPYPPQEPQQPLFPQKEPQQPFPLQQPQYQQQQPYPQQPLPQEQLPQQ
HLFPQQPPQQQFPQQMPLPHQQQTFPQQQQQQQQEQLPQQLPQFPQQQPFSQYQQPLTQQPYSQEQPLPQQQPSVEEKQQLNLCKEFLLQQCN
PEEKLSLLQSVIPFLRPKTSQQNSCQLKRLQCCRQLAHISEPSRCPAIHNIVHAIVMQQQHVDRGFGQPQPQQLGQEMPMQPQHQLGQHSILPQ
QLAQYKLVRLLVIQTLPMLCNVHVPSDCYTITAPFGSMTAYNGGQ*

4) Transcript and deduced protein sequences for five transcribed and intact ω-gliadin genes (each transcript sequence is provided from start to stop codons)

> *Gli-ω1* transcript (1080 bp)
ATGAAGACCTTCCTCATCTTCGTCCTCCTTGCCATGGCGATGAGCATCGTCACTGCTGCTAGGCAGCTAAACCCTAGCAAGCAAGAGTTGCAAT
CACCACAACAATTATATCCGCAGCAACCATATCCACAGCAACCATATCCACCACAACAACCATTTCCCACACCCCAACAATATTTCCCCCATCA
ATCACAACAACCATTTTCCCCACCACAACAATCATTTCCCCAACCCCAACAAGCAACCCCCCTACAACCACAACAACCATTCCCCCAGCAACCC
CAACAACCACAACAAGCTTTTCCCCAACCCCAACAACAATTTGCCTTGCAACCACAACAACAATTTCCCCAGCTCCAACAACCACAACAATCAT
TCCCACAACAACCCCAGAGACCACACCCATTCCCCCAACAACTAGAACAAGTAATTTCACAGCAACCACAACAACCATTCCTCCTGCAACCGCA
ACAACCATTCCCCCAGCAACCAGAACAAATAATATCCCAGCAACCCCAACAACTATTTTCTCAGTCACAACAACCATTTCCCCAGCAACCCCAA
CAACCATTTCCCCTGCAACCGCAACAACCATTTCCCCAACAACCAGCACAAATAATTGCTCAGCAACCTCAACAACCATCCCCTCTGCAACCAC
AACAACCGTTCCTCCGGCAACCACAACAATCGTTCCTCCAGCAACCACAACAACCATTTCCCCAACCCCAACAAGTAGTACAAATAAATCCCCA
GCAACCCCAACAACCATTTTCCCAGTCACGACAACCATTTCCCCAGCAACCCCAACAACCATTCCCACTGCAACCGCAACAACCATTTCCCCAG
CAATCAGCACAAATAATTCCTCAGCAACCCCAACAACCATTCCCTCTACAACCACAACAATCATTCCTTCGGCAATCACAACAACCGTTCCTCC
AACAACCACAACAACCATCTCCCCAACCCCAACAAGTAGTACAAATAATTCCCCAGCAACCCCAACAACCATTCCCTCTGCTGGCAAACCAACC
TCAGCAACCTTATCCACAACAGCAACCATCAGGAGTAATGGTATAG

> Gli-ω1 deduced protein (359 aa)
MKTFLIFVLLAMAMSIVTAARQLNPSKQELQSPQQLYPQQPYPQQPYPPQQPFPTPQQYFPHQSQQPFSPPQQSFPQPQQATPLQPQQPFPQQP
QQPQQAFPQPQQQFALQPQQQFPQLQQPQQSFPQQPQRPHPFPQQLEQVISQQPQQPFLLQPQQPFPQQPEQIISQQPQQLFSQSQQPFPQQPQ
QPFPLQPQQPFPQQPAQIIAQQPQQPSPLQPQQPFLRQPQQSFLQQPQQPFPQPQQVVQINPQQPQQPFSQSRQPFPQQPQQPFPLQPQQPFPQ
QSAQIIPQQPQQPFPLQPQQSFLRQSQQPFLQQPQQPSPQPQQVVQIIPQQPQQPFPLLANQPQQPYPQQQPSGVMV*

> *Gli-ω2* transcript (1221 bp)
ATGAAGACCTTCCTCATCTTGGTCCTCCTTGCCATGGCGACGAGCATGGTCACTGCTGCTAGGCCGCTAAACCCTAGCGACCAAGAGTTGCAAT
CACCACAACAACAATTTCCGGAAGAACAATCATATCCGCAGCAACCATATCCACAGCAAGCATTTCCCATACCCCAACAATATTCCCCGCATCA
ACCACAACAACCATTTCCTCAACCCCAACGACCAACCCCCCTCCAACCACAACAACCATTCCCCCAGCAACCCAAACAACCACAACAATCTTTT
CCCCAGCCCCAACAACAATTCCCCTTGCAACCACAACAACCATTTCCCCAGCCTCAACAGCCGATTCCCCAACAACCACGAATACCATTCCCGG
AGCAACCCCAGAGACCCCAACAACCACAACAATCTTTTCCTCAGCCCCAACAACAATTCCCCTTGCAACCACAACAACCATTTCCCCAGCCCCA
ACAACCACAACAACCATTCCCCCAGCAACCCCAGAGACCACAACAATCATTCCCCCAGCAACCAGAACAAATAATTCCCCAGCAACCGCAACAA
CCATTCCCCCTACAACCGCAACAACCATTCCCCCAGCAACCAGAACAAATAATTTCCCAGCAATCCCAACAACCATTCTCTCTGCAGCCACAAC
AACCATTTTCCCAGCCCCAACAACCATTATCCCAGCAACCAGGACAAATAATTCCCCAGCAATCCCAACAACCATGCCCCCTCCAACCACAACA
ACCATTCCCCCAGCAACCAGAACAAATAATTCGCCAGCAACCGCAACAACCATTCCTCCTACAATCGCAACAACCATTCCACCAGCAACCAGAA

	CAAATAATTTCTCAGCAACCCCAAAAACCATTCTCTCTGCAGCCCCAACAACCATTTTCCCAGCCCCAACAACCATTTCCCCAGCAACCAGGAC<br>AAATAATTCCCCAGCAACCCCAACAACCATTCTCCCTGCAACCTCAACAACCATTCCCCCAGCAACTCCAACAACCATTCGCTCTGCAACCACA<br>ACAATCGTTCCCCCAGCAACCACAACAACCATTTCCCCAACCCCAACAAGCATTCCCAGAACAAGCCAGAACAGATAATTCCCCAGCAACCCTA<br>ACAACCATTCCCTCTGCAGCCACACCAACCTCAGCAACCTTATCCACAACAACAACCATCTGGAGTAGCGGTATAGGCATCAGGGGCCAATGA<br>> Gli-ω2 deduced protein (406 aa)<br>MKTFLILVLLAMATSMVTAARPLNPSDQELQSPQQQFPEEQSYPQQPYPQQAFPIPQQYSPHQPQQPFPQPQRPTPLQPQQPFPQQPKQPQQSF<br>PQPQQQFPLQPQQPFPQPQQPIPQQPRIPFPEQPQRPQQPQQSFPQPQQQFPLQPQQPFPQPQQPQQPFPQQPQRPQQSFPQQPEQIIPQQPQQ<br>PFPLQPQQPFPQQPEQIISQQSQQPFSLQPQQPFSQPQQPLSQQPGQIIPQQSQQPCPLQPQQPFPQQPEQIIRQQPQQPFLLQSQQPFHQQPE<br>QIISQQPQKPFSLQPQQPFSQPQQPFPQQPGQIIPQQPQQPFSLQPQQPFPQQLQQPFALQPQQSFPQQPQQPFPQPQQAFPEQARTDNSPATL<br>TTIPSAATPTSATLSTTTTIWSSGIGIRGQ*<br><br>> *Gli-ω3* transcript (1320 bp)<br>ATGAAGACCTTCATCATATTTGTCCTCCTTGCCATGGCGATGAACATCGCCAGTGCCAGTAGGCTGCTAAGCCCTAGAGGCAAGGAATTGCATA<br>CTCCACAAGAACAATTCCCCCAACAACAACAATTCCCCCAACCACAACAATTCCCCCAACAACAAATCCCCCAACAACATCAAATCCCCCAGCA<br>ACCACAACAATTCCCCCAACAACAACAATTCTTCCAACAACAACAAATCCCGCAACAACAAATCCCCCAACAACATCAAATCCCCCAGCAACCA<br>CAACAATTCCCCCAGCAACAGCAATTCCCCCAACAACACCAATCCCCCCAACAACAATTCCCACAACAACAATTCCCCCAACAGAAATTGCCGC<br>AACAGGAATTCCCACAACAACAAATCTCCCAGCAACCACAACAACTCCCCCAGCAACATCAAATCCCCCAGCAACCTCAACAATTTCTCCAACA<br>ACAACAATTCCCCCAGCAACAACCCCCCCAACAACATCAATTTCCCCAACAACAATTGCCCCAACAACAACAAATCCCCCAACAACAACAAATC<br>CCCCAGCAACCACAACAAATCCCCCAACAACAACAAATCCCCCAGCAACCACAACAATTCCCCCAACAACAATTCCCGCAACAACAATTTCCCC<br>AACAGCAATTCCCGCAACAGGAATTCCCACAACAACAACAATTCCCACAACAACAAATCGCCCAGCAACCACAACAACTCCCCCAACAACAACA<br>AATCCCCCAGCAACCACAACAATTTCCCCAACAACAACAATTCCCCCAGCAACAATCACCCCAACAACAGCAATTTCCCCAACAACAATTCCCC<br>CAACAACAACAATTACCGCAAAAACAATTCCCCCAACCACAACAAATACCCCAACAACAACAAATCCCCCAGCAACCACAACAATTCCCCCAGC<br>AACAATTCCCCCAACAACAGCAATTTCCCCAACAACAAGAATTCTCCCCACAGCAATTCCCGCAACAACAATTCCACCAACAACAATTACCGCA<br>ACAACAATTTCCCCAACAACAATTCCCCCAACAACAATTCCCCCAACAACAACAGTTCCCCCAACAACAACAATTAACGCAACAACAATTCCCC<br>CGGCCACAACAATACCCTGAACAACAACAATTCCCCCAACAACAATTCCCCCAGCAACCACCACAACAATTCCCCCAACAACAATTTCCAATAC<br>CATACCCACCCCAGCAATCACAAGAACCTTCCCCATACCAACAATATCCACAACAACAACCATCTGGGAGCGACGTTATAAGTATCAGTGGCCT<br>ATGA<br>> Gli-ω3 deduced protein (439 aa)<br>MKTFIIFVLLAMAMNIASASRLLSPRGKELHTPQEQFPQQQQFPQPQQFPQQQIPQQHQIPQQPQQFPQQQQFFQQQQIPQQQIPQQHQIPQQP<br>QQFPQQQQFPQQHQSPQQQFPQQQFPQQKLPQQEFPQQQISQQPQQLPQQHQIPQQPQQFLQQQQFPQQQPPQQHQFPQQQLPQQQQIPQQQQI<br>PQQPQQIPQQQQIPQQPQQFPQQQFPQQQFPQQQFPQQEFPQQQQFPQQQIAQQPQQLPQQQQIPQQPQQFPQQQQFPQQQSPQQQQFPQQQFP<br>QQQQLPQKQFPQPQQIPQQQQIPQQPQQFPQQQFPQQQQFPQQQEFSPQQFPQQQFHQQQLPQQQFPQQQFPQQQFPQQQQFPQQQQLTQQQFP

RPQQYPEQQQFPQQQFPQQPPQQFPQQQFPIPYPPQQSQEPSPYQQYPQQQPSGSDVISISGL*

> *Gli-ω4* transcript (1164 bp)
ATGAAGACCTTCCTCATCTTTGTCCTCCTTGCCATGGCGATGAAGATCGCCACTGCCGCTAGGGAGTTAAACCCTAGCAACAAAGAGTTACAAT
CACCTCAACAATCATTTTCCCATCAACAACAACCATTTCCACAGCAGCCATATCCACAACAACCATATCCATCACAGCAACCATATCCATCGCA
ACAACCATTTCCCACACCCCAACAACAATTTCCCCAGCAATCACAACAACCATTTACCCAGCCCCAACAACCGACCCCCTTACAACCACAACAA
CCATTCCCCCAGCAACCCCAACAACCACAACAACCTTTTCCACAACCCCAACAACCATTTCCCTGGCAACCACAACAACCATTTCCCCAGACCC
AACAATCGTTCCCTCTCCAACCACAACAGCCATTCCCCCAGCAACCCCAACAACCATTTCCCCAGCCCCAACTACCATTCCCCCAGCAATCAGA
ACAAATAATTCCCCAGCAACCCCAACAACCATTCCCCCTGCAACCGCAACAACCATTCCCCCAGCAACCCCAACAACCATTTCCCCAGCCCCAA
CAACCAATCCCCGTGCAACCACAACAATCATTCCCCCAACAATCCCAACAATCACAACAACCTTTTGCCCAGCCCCAACAATTATTTCCTGAAC
TCCAACAACCAATTCCCCAGCAACCACAACAACCATTCCCCCTGCAACCGCAACAACCATTCCCCCAGCAACCGCAACAACCATTCCCCCAGCA
ACCGCAACAATCATTTCCCCAGCAACCACAACAACCATTCCCCCAGCAACCGCAACAACCATTCCCCCAACAACCACAACAACCATTCCCTCAA
CAACCACAACAACCATTCCCCCTACGACCGCAACAACCATTTCCCCAGCAACCCCAACAATCACAACAATCATTTCCCCAGCCCCAACCCCAAC
CCCAGCAACCCCCACAACCATCCATCCTTCAACCACAACAACCATTACCCCAACAACCACAACAACCATTTCAACAGCCCCAACAACAATTATC
CCAGCAACCAGAACAAACAATTTCCCAGCAACCCCAACAACCATTCCCCCAGCAACCACACCAACCTCAACAACCATATCCACAACAACAACCA
TATGGGAGTAGTCTTACAAGCATCGGTGGCCAATGA

> Gli-ω4 deduced protein (387 aa)
MKTFLIFVLLAMAMKIATAARELNPSNKELQSPQQSFSHQQQPFPQQPYPQQPYPSQQPYPSQQPFPTPQQQFPQQSQQPFTQPQQPTPLQPQQ
PFPQQPQQPQQPFPQPQQPFPWQPQQPFPQTQQSFPLQPQQPFPQQPQQPFPQPQLPFPQQSEQIIPQQPQQPFPLQPQQPFPQQPQQPFPQPQ
QPIPVQPQQSFPQQSQQSQQPFAQPQQLFPELQQPIPQQPQQPFPLQPQQPFPQQPQQPFPQQPQQSFPQQPQQPFPQQPQQPFPQQPQQPFPQ
QPQQPFPLRPQQPFPQQPQQSQQSFPQPQPQPQQPSQPSILQPQQPLPQQPQQPFQQPQQQLSQQPEQTISQQPQQPFPQQPHQPQQPYPQQQP
YGSSLTSIGGQ*

> *Gli-ω5* transcript (1134 bp)
ATGAAGACCTTCCTCATCTTTGTCCTCCTTGCCATGGCGATGAACATCGCCACTGCTGCTAGGCAGCTAAACCCTAGCAACAAAGAGCTACAAT
CACCTCAACAATCATTTTCCCATCAACAACAACCATTTCCACAGCAACCATATCCACAACAACCATATCCATCACAGCAACCATATCCATCGCA
ACAACCATTTCCCACACCCCAACCACAATTTCCCCAGCAATCACAACAACCATTTACCCAGCCCCAACAACCGACCCCCCTACAACCACAACAA
CCATTCCCCCAGCAACCCCAACAACCACAACAACCTTTTCCACAGCCGCAACAACCATTTCCCTGGCAACCACAACAACCATTTCCCCAGACCC
AACAATCTTTCCCTCTGCAACCACAACAACCATTCCCCCAGCAACCCCAACAACCATTTCCCCAGCCCCAACTACAATTCCCCCAGCAACCAGA
ACAAATAATTCCCCAGCAACCCCAACAACCATTCCTCCTGGAATCGCAACAACCATTCCCCCAGCAACCCCAACAACCATTTCCCCAGCCCCAA
CAACTGATCCCCATGCAACCACAACAACCATTCCCCCAGCAATCCCAACAATCACAACAACCTTTTCCCGGGCCCCAACAATTATTTCCTGAAC
TCCAACAACCAATTCCCCAGCAACCACAACAACCATTCCCCCTGCAACCGCAACAACCATTCCCCCAGCAATCGCAACAACCATTCCCCCAGCA

<table>
<tr><td></td><td>ACCCCAACAACCATGCCCCCTGCAACCGCAACAACCATTCCCCCAACAACCACAACAACCATTCCCCCAACAACCACAACAACCTTTCCCCCTA
CAACCACAACAACCATTCCCCCTACGACCGCAACAACCATTTTCCCAGCAACCCCAACAATCACAACAATCATTTCCCCAGCCCCAACCCCAGC
AACCCCAACAACCATCCATCCTGCAACCACAACAACCATTTCTGCAGCCCCAACAACAATTATCCCAGCAACTAGAACAAACAATTTCCCAGCA
ACCCCAACAACCATTCCCCCAGCAACCACACCAACCTCAACAACCATATCCACAACAACAACCATATGGGAGTAGTCTTACAAGCATCGGTGGC
CAATGA
> Gli-ω5 deduced protein (377 aa)
MKTFLIFVLLAMAMNIATAARQLNPSNKELQSPQQSFSHQQQPFPQQPYPQQPYPSQQPYPSQQPFPTPQPQFPQQSQQPFTQPQQPTPLQPQQ
PFPQQPQQPQQPFPQPQQPFPWQPQQPFPQTQQSFPLQPQQPFPQQPQQPFPQPQLQFPQQPEQIIPQQPQQPFLLESQQPFPQQPQQPFPQPQ
QLIPMQPQQPFPQQSQQSQQPFPGPQQLFPELQQPIPQQPQQPFPLQPQQPFPQQSQQPFPQQPQQPCPLQPQQPFPQQPQQPFPQQPQQPFPL
QPQQPFPLRPQQPFSQQPQQSQQSFPQPQPQQPQQPSILQPQQPFLQPQQQLSQQLEQTISQQPQQPFPQQPHQPQQPYPQQQPYGSSLTSIGG
Q*</td></tr>
<tr><td>Inactive members</td><td>5) Transcript sequence for 10 inactive gliadin genes (each transcript sequence is provided from 5' to 3' end)

>Gli-α-ψ1 transcript (868 bp, coding sequence interrupted)
ATGAAGACATTTCTCATCCTTGCCCTCCTTGCTATCGTGGCGACCACCGCCACAACTGCAGTTAGAGTTCCAGTGCCACAATTGCAGCCACAAA
ATCCATCTCAGCAACAGCCACAAGAGCAAGTTCCATTGGTACAACAACAACAATTTCTAGGGCAGCAACAACCATTTCCACCACAACAACCATA
TCCACAGCCGCAACCATTTCCATCACAACAACCATATCTGCAGCTGCAACCATTTTCGCAGCCGCAACTACCATATTCGCAGCCACAACCATTT
CGACCACAACAACCATATCCACAACCGCAACCACAGTATTCGCAACCACAACAACCAATTTCACAGCAGCAGCAGCAGCCAGCAACAACAACAA
CAACAACAACAACAACAACAAGAACAACAAATCCTTCAACAAATTTTGCAACAACAACTGACTCCATGCATGGATGTTGTATTGCAGCAACACA
ACATAGCGCGTGGAAGATCACAAGTTTTGCAACAAAGTACTTACCAGCTGTTGCAAGAATTGTGTTGTCAGCACCTATGGCAGATCCCTGAGAA
GTTGCAGTGCCAGGCCATCCACAATGTTGTTCATGCTATTATTCTGCATCAACAACAAAAACAACAACAACAACTATCGAGCCAGGTCTCCTTC
CAACAGCCTCAGCAACAATATCCATTAGGCCAGGGCTCCTTCCGGCCATCTCAGCAAAACCCACAGGCCCAGGGCTCTGTCCAGCCTCAACAAC
TGCCCCAGTTCGAGGAAATAAGGAACCTAGCGCTACAGACGCTACCTGCAATGTGCAATGTCTACATCCCTCCATATTGCACCATCGCGCCATT
TGGCATCTTCGGTACTAATTGA

>Gli-α-ψ2 transcript (863 bp, coding sequence interrupted)
ATGAAGACCTTTCTCATCCTTGTCCTCCTTGCTATTGTGGCGACCACCGCCACAACTGCAGTTAGATTTCCAGTGCCACAATTGCAGCCACAAA
AATCCATCTCAGCAACAGCCACAAGAGCAAGTTCCATTGGTACAACAACAACAATTTCTAGGGCAGCAACAACCATTTCCACCACAACAACCAT
ATCCACAGCCGCAACCATTTCCATCACAACTACCATATCTGCAGCTGCAACCATTTCCGCAGCCGCAACTACCATATTCACAGCCACAACCATT
TCGACCACAACAACCATATCCACAACCGCAACCACAGTATTCGCAACCACAACAACCAATTTCACAGCAGCAGCAGCAGCAGCAGCAGCAGCAA
CAACAACAACAACAACAACAACAAATCCTTCAACAAATTTTGCAACAACAACTGATTCCATGCATGGATGTTGTATTGCAGCAACACAACATAG
CGCATGGAAGATCACAAGTTTTGCAACAAAGTACTTACCAGCTGTTGCAAGAATTGTGTTGTCAACACCTATGGCAGATCCCTGAGCAGTCGCA</td></tr>
</table>

GTGCCAGGCCATCCACAATGTTGTTCATGCTATTATTCTGCATCAACAACAAAAACAACAACAAACAACCATCGAGCCAGGTCTCCTTCCAACA
GCCTCTGCAACAATATCCATTAGGCCAGGGCTCCTTCCGGCCATCTCAGCAAAACCCACAGGCCCAGGGCTCTGTCCAGCCTCAACAACTGCCC
CAGTTCGAGGAAATAAGGAACCTAGCGCTACAGACGCTACCTGCAATGTGCAATGTCTACATCCCTCCATATTGCACCATCGCGCCATTTGGCA
TCTTCGGTACTAACTGA

>*Gli-α-ψ3* transcript (1386 bp, coding sequence interrupted)
ATGGCAACAACCATCGCCACCGCCAATATGCAAGTCGACCCCGGCTACCAAGTACAATGGCCACAACAACAACCATTCCCCCAGCCCCAACAAC
CATTCTGCCAGCAACCACAACAAACTATTCCCCAACCCCATCAAACGTTCCACCATCAACCACAGCAAACATACCCCATCAACCACAACAACAA
TTTCCCCAGACCCAGCAACCACAACAACCATTTCCCCAGCCCCAACAAACATTCCCCCAACAACCCCAACTACCATTTCCCCAACAACCCCAAC
AACCATTCCCCCAGCCTCAACAACCCCAACAACAATTTCCCCAGTCACAGCAACCACAACAACCTTTTCCCCAGCCCCAACAACAATTCCTGCA
GCCCCAACAACCGCAACAATCATTCCCCCAGCAACAACAACCGTTGATTCAGCTATCTCTACAACAACAGATGAACCCCTGCAAGAATTTTCTC
TTGCAGCAATGCAACCCTGTGTCATTGGTGTCATCCCTCATATCAATGATCTTGCACGAAGTGATTGCCAGGTGATGCAGCAACAATGTTGCCA
ACAACTGGCACAGATTCCTCAGCAGTTCCAGTGCCACAATTGCAGCCACAAAATCCATCTCAGCAACAGCCACAAGAGCAAGTTCCATTGGTAC
AACAACAACAATTTCTAGGGCAGCAACAACCATTTCCACCACAACAACCATATCCACAGCCGCAACCATTTCCATCACAACAACCATATCTGCA
GCTGCAACCATTTCTGCAGCCGCAACTACCATATTCACAGCCACAACCATTTCGACCACAACAACCATATCCACAACCACAACCACAGTATTCG
CAACCACAACAACCAATTTCACAGCAGCAGCAGCAGCAGCAACAACAACAACAACAACAACAACAACAACAACAACAAATCCTTCAACAAATTT
TGCAACAACAACTGATTCCATGCATGGATGTTGTATTGCAGCAACACAACATAGCCCATGGAAGATCACAAGTTTTGCAACAAAGTACTTACCA
GCTGCTGCAAGAATTGTGTTGTCAGCACCTATGGCAGATCCCTGAGCAGTCGCAGTGCCAGGCCATCCACAATGTTGTTCATGCTATTATTCTG
CATCAACAACAAAAACAACAACAACAACCATCGAGCCAGGTCTCCTTCCAACAGCCTCTGCAACAATATCCATTAGGCCAGGGCTCCTTCCGGC
CATCTCAGCAAAACCCACAGGCCCAGGGCTCTGTCCAGCCTCAACAACTGCCCCAGTTCGAGGAAATAAGGAACCAAGCGCTACAGACGCTACC
TGCAATGTGCAATGTCTACATCCCTCCATATTGCACCATCGCGCCATTTGGCATCTTCGGTACTAACTGA

>*Gli-α-ψ4* transcript (1364 bp, coding sequence interrupted)
ATGAAGACCATTCTCATCCTTGCCCTCCTTACTATCGTGGCGACCACCGCCACAACTGCAGTTAGAGTCCAAGTTCCACAATTGTAGCCACAAA
ATCCATCTCAGCAACAGCCACAAGAGCAAGTTCCATTGGTACAACAACAACAATTTCCAGGGCAGCAACAACCATTTCCACCACAACAGCCATA
TCCGCGGCCGCAACCATTTTCATCACAACAACCATATCTGCAGCTGCAACCATTTCCGCAGCCGCAACTACCATATCCGCAGCCGCAACCATTT
CGACCACAACAACCATATCCACAACCGCAACCACAGTATTAGCAACCACAACAACCAATTTCGCAGCTGCAGCAGCAACAACAACAACAATAGC
AACAACAACAGCAACAAAAACAGCAGCAACAACAACAGCAGCAGCAACAACAACAACAACAGCAACAACACCAGCAACAACAGCACCAACAGCA
GTAGCAGCAGCAGCAGCAACAACAACAACAACAACAACAACAGCAGCAGCAGCAGCAGCAGCAGCAGCAGCAGCAGCAGCAGCAGCAGCAACAA
CAACAACAACAACAACAACAACAACAACAACAACAACAACAGCAGCAGCAGCAGCAGCAGCAGCAGCAACAACAACAACAACAATAACAGCAAC
AACCATTTCCACCACAACAACCATATCCACAGCCGCAACCATTTCCATCACAACAACCATATCTGCAACTGCAACCATTTCCGCAGCCGCAACT
ACCATATTCACAGCCACAACCATTTCGACCACAACAACCATATCCACAACCGCAACCACAGTATTCGCAACCACAACAACCAATTTCACAGCAG

CAGCAGCAGCAGCAGCAACAACAACAACAACAACAACAACAACAACAACAACAACAAATCCTTCAACAAATTTTGCAACAACAACTGATTCCAT
GCATGGATGTTGTATTGCAGCAACACAACATAGCGCATGGAAGATCACAAGTTTTGCAACAAAGTACTTACCAGCTGTTGCAAGAATTGTGTTG
TCAGCACCTATGGCAGATCCCTGAGCAGTCGCAGTGCCAGGCCATCCACAAAGTTGTTCATGCTATTATTCTGCATCAACAACAAAAACAACAA
CAACAACCATCGAGCCAGGTCTCCTTCCAACAGCCTCTGCAACAATATCCATTAGGCCAGGGCTCCTTCGGCCATCTCAGCAAAACCCACAGGC
CCAGGGCTCTGTCCAGCCTCAACAACTGCCCCAGTTCGAGGAAATAAGGAACCTAGCGCTACAGACGCTACCTGCAATGTGCAATGTCTACATC
CCTCCATATTGCACCATCACGCCATTTGGCATCTTCGATACTAACTGA

>*Gli*-*α*-*ψ5* transcript (804 bp, coding sequence interrupted)
CAGCCACAAAATCCATCTCAGCAACAGCCACAAGAGCAAGTTCCGTTGGTACAACAACAACAATTTCTAGGGCAGCAACAACCATTTCCACCAC
AACAACCATATCCACAGCCGCAACCATTTCCATCACAAACAACCATATCTGCAACTGCAACCATTTCCGCAGCCGCAACTATCATATTCGCAGC
CACAACCATTTCGACCACAACAACTATATCCACAACCGCAACCACAGTATTCGCAACCACAACAACCAATTTCACAGCAGCAGCAGCAACAACA
ACAACAACAACAACAACAACAACAACAACAACAACAACAACAACAACAACAAGAACAACAAATCCTTCAACAAATTTTGCAACAACAACTGATT
CCATGCATGGATGTTGTATTGCAGCAACACAACATAGCGCATGGAAGATCACAAGTTTTGCAACAAAGTACTTACCAGCTGTTGCAAGAATTGT
GTTGTCAGCACCTATGGCAGATCCCTGAGCAGTCGCAGTGCCAGGCCATCCACAATGTTGTTCATGCTATTATTCTGCATCAACAACAAAAACA
ACAACAACAACCATCGAGCCAGGTCTCCTTCCAACAGCCTCTGCAACAATATCCATTAGGCCAGGGCTCCTTCCGGCCATCTCAGCAAAACCCA
CAGGCCAGGGCTCTGTCCAGCCTCAACAACTGCCCCAGTTCGAGGAAATAAGGAACCTAGCGCTACAGACGCTACCTGCAATGTGCAATGTCTA
CATCCCTCCATATTGCACCATCGCGCCATTTGGCATCTTCGGTACTAACTGA

>*Gli*-*α*-*ψ6* transcript (805 bp, coding sequence interrupted)
CAGCCACAAAATCCATCTCAGCAACAGCCACAAGAGCAAGTTCCGTTGGTACAACAACAACAATTTCTAGGGCAGCAACAACCATTTCCACCAC
AACAACCATATCCACAGCCGCAACCATTTCCATCACAAACAACCATATCTGCAACTGCAACCATTTCCGCAGCCGCAACTATCATATTCGCAGC
CACAACCATTTCGACCACAACAACCATATCCACAACCGCAACCACAGTATTCGCAACCACAACAACCAATTTCACAGCAGCAGCAGCAGCAACA
ACAACAACAACAACAACAACAACAACAACAACAACAACAACAACAACAACAAGAACAACAAATCCTTCAACAAATTTTGCAACAACAACTGATT
CCATGCATGGATGTTGTATTGCAGCAACACAACATAGCGCATGGAAGATCACAAGTTTTGCAACAAAGTACTTACCAGCTGTTGCAAGAATTGT
GTTGTCAGCACCTATGGCAGATCCCTGAGCAGTCGCAGTGCCAGGCCATCCACAATGTTGTTCATGCTATTATTCTGCATCAACAACAAAAACA
ACAACAACAACCATCGAGCCAGGTCTCCTTCCAACAGCCTCTGCAACAATATCCATTAGGCCCAGGGCTCCTTCCGGCCATCTCAGCAAAACCC
ACAGGCCAGGGCTCTGTCCAGCCTCAACAACTGCCCCAGTTCGAGGAAATAAGGAACCTAGCGCTACAGACGCTACCTGCAATGTGCAATGTCT
ACATCCCTCCATATTGCACCATCGCGCCATTTGGCATCTTCGGTACTAACTGA

>*Gli*-*α*-*ψ7* transcript (887 bp, coding sequence interrupted)
ATGAAGACCTTTCTCATCCTTGCCCTCCTTACTATCGTGGCGACCACCGCCACAACTGCAGTTAGAGTTCCAGTGCCACAATTGCAGCCACAAA
ATCCATCTCAGCAACACCCACAAGAGCAAGTTCCGTTGGTACAACAACAACAATTTCTAGGGCAGCAACAATCATTTCCACCACAACAACCATA

	TCCACAGCCGCAACCATTTCCATCACAACAACCATATCTGCAGCTGCAACCATTTCCGCAGCCGCAACTACCATATTTGCAGCCACAACCATTT<br>CGACCACAACAACCATATCCACAACCGCAACCACAATATTCGCAACCACAACAACCAATTTCACAGCAGCAGCAGCAGCAGCAACAACAACAAC<br>AACAACAACAACAACAACAACAACAACAACAACAACAACAACAACAGCAAATCCTTCAACAAATTTTGCAACAACAACTGATTCCATGCATGGA<br>TGTTGTATTGCAGCAACACAACATAGCGCATGGAAGATCACAAGTTTTGCAACAAAGTACTTACCAGCTGTTGCAAGAATTGTGTTGTCAGCAC<br>CTATGGCAGATCCCTGAGCAGTCGCAGTGCCAGGCCATCCACAATGTTGTTCATGCTATTATTCTGCATCAACAACAAAAACAACAACAACAAC<br>CATCGAGCCAGGTCTCCTTCCAACAGCCTCTGCAACAATATCATTAGGCCAGGGCTCCTTCCGGCCATCTCAGCAAAACCCACTGGCCCAGGGC<br>TCTGTCCAGCCTCAACAACTGCCCCAGTTCGAGGAAATAAGGAACCTAGCGCTACAGACGCTACCTGCAATGTGCAATGTCTACATCCCTCCAT<br>ATTGCACCATCGTGCCATTTGGCATCTTCAGTACTAACTGA<br><br>>*Gli-γ-ψ1* transcript (863 bp, coding sequence interrupted)<br>ATGGCAATAACCATCGGCACCGCCAATATGCAGGTCGACCCTAGCAGCCAAGTACAATGGCCACAACAACAACCAGTCCCACAGCCTCACCAAC<br>CATTCTCCCAGCAACCACAACAAACATTTCCCAACCCCCAACAAACATTCCCCCATCAACCACAACAACAATTTCCCCAGCCTCAGCAACCACA<br>ACAACAATTTCTCCAGCCCCAACAACCATTCCCCCAACAACCACAACAACCATATCCCCAGCAACCACAACAACCATTCCCCAGACTCAACAAC<br>CCCAACAACTATTTCCCCAGTCCCAGCAACCACAACAACAATTTTCTCAGCCCCAACAACAATTCCCGCAGCCCCAACAACCGCAACAATCATT<br>CCCCCAACAACAACCACCGTTCATTCAGCCATCTCTACAACAACAGGTGAACCCATGCAAGAATTTCCTCTTGCAGCAATGCAAACCTGTGTCA<br>CTGGTGTCATCCCTCTGGTCAATGATCTGGCCACAAAGCGATTGCCAAGTGATGCGGCAACAATGCTGCCAACAACTAGCACAGATTCCTCAGC<br>AGCTCCAGTGTGCAGCCATCCATACCATCATACATTCCATCATCATGCAGCAAGAACAACAAGAACAACAACAAGGCATGCATATCCTGCTGCC<br>ACTATATCAGCAGCAACAGGTGGGTCAAGGTACTCTCGTCCAGGGCCAGGGCATCATCCAACCCCAACAACCAGCTCAATTGGAGGCGATCAGG<br>TCATTGGTGTTGCAAACTCTTCCAACCATGTGCAACGTGTATGTCCCACCTGAGTGCTCCATCATCAAGGCACCATTTTCCAGCGTAGTCGCCG<br>GCATTGGTGGCCAATGA<br><br>>*Gli-γ-ψ2* transcript (1201 bp, coding sequence interrupted)<br>ATGAAGACCTTACTCATCCTAACAATCCTTGCGATGGCAACAATAATCGCCACTGCCAATATGCAGGTCGACCTAGCAGCCGAGTACAATGGCC<br>ACAAGAACAACCATCCCCCCAGTCCCAACAACCATTCTCCAGCAACCACAACAAATATTTCCCCAACCCCAACAAACATTGCCCCATCAACCAC<br>AACAAGCATTTCCCCAACCTCAACAAACATTCCCCCATCGACCACAACAACAATTTCCCCAGCCCCAGCAACCACAACAACCATTTCCTCAGCC<br>CCAACAACCCCAACTACCATTTCCCCAACAACTACAACAACAACTAGTCCCCAGCTCCAACAGCCATTATCCAGCAACCACAACAAACATTTCC<br>CCAACCTCAACAAACATTCCCCATCAACCACAACAACAAGTTCCCCAGCCTCAGCAACCACAACAACCATTTCTCCAGCCCCAACAACCATTCC<br>CCCAACAACCACAACAACCATTCCCCCAGACTCAACAACCACAACAACCATTTCCCCAGCAACCACAACAACCATTTCCCCAGACTCAACAACC<br>CCAACAACCATTTCCCCAACAACCACAACAACCATTCCCCCAGACTCAACAACCCCAACAACCATTTCCCCAGCTCCAGCAACCACAACAACCT<br>TTTCCCCAGCCCCAACAACAATTGCCGCAGCCCCAACAACCGCAACAATCATTCCCCCAACAACAACGGCCATTCATTCAACCATCTCTACAAC<br>AACAGTTGAACCCATGCAAGAATATCCTCTTGCAACAATGCAAACCTGCGTCATTGGTGTCATCCCTCTGGTCAATAATCTGGCCACAAAGCGA<br>TTGCCAAGTGATGCGGCAACAATGCTGCCAACAACTAGCACAGATTCCTCAACAGCTCCAGTGCGCAGCCATCCATAGCGTCGTGCATTCCATC

	ATCATGCAGCAGCAGCAGCAACAACAACAACAACAAGGCATGCATATCTTTCTGCCACTATCTCAGCAGCAACAGGTGGGTCAAGGTTCTCTAG<br>TCCAAGGCCAGGGCATCATCCAACCACAACAACCAGCTCAATTGGAGGCGATCAGATCATTGGTGTTGCAAACTCTTCCATCCATGTGCAACGT<br>GTATGTCCCACCTGAGTGCTCCATCATGAGGGCACCATTTGCCAGCATAGTCGCGGGCATTGGTGGCCAATGA<br><br>>*Gli-ω-ψ1*(1319 bp, coding sequence interrupted)<br>ATGAAGACCTTCATCATATTTGTCCTCCTTGCCATGGCGATGAACATCGCCAGTGCCAGTAGGCTGCTAAGCCCTAGAGGCAAGGAATTGCATA<br>CTCCACAAGAACAATTCCCCCAACAACAACAATTCCCCCAACCACAACAATTCCCCCAACAACAAATCCCCCAACAACATCAAATCCCCCAGCA<br>ACCACAACAATTCCCCCAACAACAACAATTCCTCCAACAACAACAAATCCCGCAACAACAAATCCCCCAACAACATCAAATCCCCCAGCAACCA<br>CAACAATTCCCCCAGCAACAGCAATTCCCCAACAACACCAATCCCCCCAACAACAATTCCCACAACAACAATTCCCCCAACAGAAATTGCCGCA<br>ACAGGAATTCCCACAACAACAAATCTCCCAGCAACCACAACAACTCCCCCAGCAACAACAAATCCCCCAGCAACCACAACAATTTCTCCAACAA<br>CAACAATTCCCCCAGCAACAACCCCCCCAACAACATCAATTTCCCCAACAGCAATTGCCCCAACAACAACAAATCCCCCAACAACAACAAATCC<br>CCCAGCAACCACAACAAATCCCCCAACAACAACAAATCCCCCAGCAACCACAACAATTCCCCCAACAACAATTCCCGCAACAACAATTTCCCCA<br>ACAGCAATTCCCGCAACAGGAATTCCCACAACAACAACAATTCCCACAACAACAAATCGCCCAGCAACCACAACAACTCCCCCAACAACAACAA<br>ATCCCCCAGCAACCACAACAATTTCCCCAACAACAACAATTCCCCCAGCAACAATCACCCCAACAACAGCAATTTCCCCAACAACAATTCCCCC<br>AACAACAACAATTACCGCAAAAACAATTCCCCCAACCACAACAAATACCCCAACAACAACAAATCCCCCAGCAACCACAACAATTCCCCCAGCA<br>ACAATTCCCCCAACAACAGCAATTTCCCCAACAACAAGAATTCTCCCAACAGCAATTCCCGCAACAACAATTCCACCAACAACAATTACCGCAA<br>CAACAATTTCCCCAACAACAATTCCCCCAACAACAATTCCCCCAACAACAACAGTTCCCCCAACAACAACAATTAACGCAACAACAATTCCCCC<br>GGCCACAACAATACCCTGAACAACAACAATTCCCCCAACAACAATTCCCCCAGCAACCACCACAACAATTCCCCCAACAACAATTTCCAATACC<br>ATACCCACCCCAGCAATCACAAGAACCTTCCCCATACCAACAATATCCACAACAACAACCATCTGGGAGCGACGTTATAAGTATCAGTGGCCTA<br>TGA

Table S4. Computation of coeliac disease (CD) epitopes in the 21 α-gliadins accumulated in Xiaoyan 81 mature grains.

		DQ2.5																DQ2.2	DQ8				DQ8.5		
α-Gliadin	*Gli* locus	DQ2.5-glia-α1[a]	DQ2.5-glia-α1[b]	DQ2.5-glia-α2	DQ2.5-glia-α3	DQ2.5-glia-γ1	DQ2.5-glia-γ2	DQ2.5-glia-γ3	DQ2.5-glia-γ4[a]	DQ2.5-glia-γ4[b]	DQ2.5-glia-γ4[c]	DQ2.5-glia-γ4[d]	DQ2.5-glia-γ5	DQ2.5-glia-ω1	DQ2.5-glia-ω2	DQ2.5-glut-L1	DQ2.5-glut-L2	DQ2.2-glut-L1	DQ8-glia-α1	DQ8-glia-γ1a	DQ8-glia-γ1b	DQ8-glut-H1	DQ8.5-glia-α1	DQ8.5-glia-γ1	DQ8.5-glut-H1
Gli-α1	*Gli-A2*	-	-	-	1	-	-	-	-	-	-	-	-	-	-	-	-	-	-	-	-	-	-	-	-
Gli-α2	(6A)	-	-	-	1	-	-	-	-	-	-	-	-	-	-	-	-	-	-	-	-	-	-	-	-
Gli-α3		1	-	-	1	-	-	-	-	-	-	-	-	-	-	-	-	-	-	-	-	-	-	-	-
Gli-α4		-	-	-	1	-	-	-	-	-	-	-	-	-	-	-	-	-	-	-	-	-	-	-	-
Gli-α5		1	-	-	1	-	-	-	-	-	-	-	-	-	-	-	-	-	-	-	-	-	-	-	-
Gli-α6		-	-	-	1	-	-	-	-	-	-	-	-	-	-	-	-	-	-	-	-	-	-	-	-
Gli-α9	*Gli-B2*	-	-	-	-	-	-	-	-	-	-	-	-	-	-	-	-	-	1	-	-	-	1	-	-
Gli-α10	(6B)	-	-	-	-	-	-	-	-	-	-	-	-	-	-	-	-	-	1	-	-	-	1	-	-
Gli-α11		-	-	-	-	-	-	-	-	-	-	-	-	-	-	-	-	-	-	-	-	-	-	-	-
Gli-α12		-	-	-	-	-	-	-	-	-	-	-	-	-	-	-	-	-	-	-	-	-	-	-	-
Gli-α13		-	-	-	-	-	-	-	-	-	-	-	-	-	-	-	-	-	-	-	-	-	-	-	-
Gli-α14		-	-	-	-	-	-	-	-	-	-	-	-	-	-	-	-	-	-	-	-	-	-	-	-
Gli-α15		-	-	-	-	-	-	-	-	-	-	-	-	-	-	-	-	-	-	-	-	-	-	-	-
Gli-α16		-	-	-	-	-	-	-	-	-	-	-	-	-	-	-	-	-	-	-	-	-	-	-	-
Gli-α17		-	-	-	-	-	-	-	-	-	-	-	-	-	-	-	-	-	-	-	-	-	-	-	-
Gli-α19	*Gli-D2*	-	-	-	-	-	-	-	-	-	-	-	-	-	-	-	-	-	-	-	-	-	-	-	-
Gli-α20	(6D)	1	1	1	1	-	-	-	-	-	-	-	-	-	-	-	-	-	1	-	-	-	1	-	-
Gli-α21		1	-	1	1	-	-	-	-	-	-	-	-	-	-	-	-	-	1	-	-	-	1	-	-
Gli-α22		1	1	2	1	-	-	-	-	-	-	-	-	-	-	-	-	-	1	-	-	-	1	-	-
Gli-α23		1	1	2	1	-	-	-	-	-	-	-	-	-	-	-	-	-	1	-	-	-	1	-	-
Gli-α24		1	-	1	1	-	-	-	-	-	-	-	-	-	-	-	-	-	-	-	-	-	-	-	-

Note: The CD epitopes are divided into four groups based on specific human leucocyte antigen class II proteins (DQ2.5, DQ2.2, DQ8 and DQ8.5) to which they bind. Each color shaded value indicates the number of times a particular epitope is found in a given gliadin protein. The eight gliadins marked in blue do not carry CD epitopes.

Table S5. Computation of coeliac disease (CD) epitopes in the 11 γ-gliadin, 1 δ-gliadin and 5 ω-gliadin proteins accumulated in Xiaoyan 81 mature grains.

Gliadin	*Gli* locus	DQ2.5																DQ2.2	DQ8				DQ8.5		
		DQ2.5-glia-α1a	DQ2.5-glia-α1b	DQ2.5-glia-α2	DQ2.5-glia-α3	DQ2.5-glia-γ1	DQ2.5-glia-γ2	DQ2.5-glia-γ3	DQ2.5-glia-γ4a	DQ2.5-glia-γ4b	DQ2.5-glia-γ4c	DQ2.5-glia-γ4d	DQ2.5-glia-γ5	DQ2.5-glia-ω1	DQ2.5-glia-ω2	DQ2.5-glut-L1	DQ2.5-glut-L2	DQ2.2-glut-L1	DQ8-glia-α1	DQ8-glia-γ1a	DQ8-glia-γ1b	DQ8-glut-H1	DQ8.5-glia-α1	DQ8.5-glia-γ1	DQ8.5-glut-H1
Gli-γ1	*Gli-A1*	-	-	-	-	-	1	-	-	-	6	-	2	1	-	-	-	-	-	6	-	-	-	-	-
Gli-γ2	(1A)	-	-	-	-	1	-	-	-	-	4	-	-	-	-	-	-	-	-	4	-	-	-	1	-
Gli-γ3		-	-	-	-	1	-	-	-	-	4	-	-	-	-	-	-	-	-	4	-	-	-	1	-
Gli-γ4	*Gli-B1*	-	-	-	-	-	-	-	-	1	6	-	3	-	-	-	-	-	-	6	-	-	-	-	-
Gli-γ5	(1B)	-	-	-	-	1	1	-	-	1	4	-	-	1	-	-	-	-	-	4	-	-	-	1	-
Gli-γ6		-	-	-	-	1	1	-	-	1	4	-	-	-	-	-	-	-	-	4	-	-	-	1	-
Gli-γ7		-	-	-	-	1	1	1	-	-	2	-	-	-	-	-	-	-	-	2	1	-	-	1	-
Gli-γ8	*Gli-D1*	-	-	-	-	1	1	-	-	-	5	-	3	-	-	-	-	-	-	5	-	-	-	1	-
Gli-γ9	(1D)	-	-	-	-	1	1	1	1	-	1	-	1	-	-	-	-	-	-	1	1	-	-	1	-
Gli-γ10		-	-	-	-	1	1	-	-	-	3	1	-	1	-	-	-	-	-	3	-	-	-	1	-
Gli-γ11		-	-	-	-	1	1	-	-	1	1	-	1	-	-	-	-	-	-	1	-	-	-	1	-
Gli-δ1	*Gli-D1* (1D)	-	-	-	-	-	-	-	-	-	-	-	-	-	-	-	-	-	-	-	-	-	-	-	-
Gli-ω1	*Gli-A1* (1A)	-	-	-	-	-	-	-	-	-	1	-	2	-	-	-	-	-	-	1	-	-	-	-	-
Gli-ω2	*Gli-B1*	-	-	-	-	-	-	-	-	-	2	-	1	-	-	-	-	-	-	2	-	-	-	-	-
Gli-ω3	(1B)	-	-	-	-	-	-	-	-	-	-	-	-	-	-	-	-	-	-	-	-	-	-	-	-
Gli-ω4	*Gli-D1*	-	-	-	-	-	-	-	-	-	7	-	7	1	1	-	-	-	-	7	-	-	-	-	-
Gli-ω5	(1D)	-	-	-	-	-	-	-	-	-	5	-	5	1	1	-	-	-	-	5	-	-	-	-	-

Note: The CD epitopes are divided into four groups based on specific human leucocyte antigen class II proteins (DQ2.5, DQ2.2, DQ8 and DQ8.5) to which they bind. Each color shaded value indicates the number of times a particular epitope is found in a given gliadin protein. The two gliadins marked in blue do not carry CD epitopes.

Table S6. PCR primers used for microsatellite marker analysis of the six *Gli* locus deletion lines.

Marker	Primer sequence (5' - 3')	Chromosomal location	For the analysis of
Xgdm33	F: GGCTCAATTCAACCGTTCTT<br>R: TACGTTCTGGTGGCTGCTC	1AS, -5.1 cM	DLGliA1
Xcfa2153	F: TTGTGCATGATGGCTTCAAT<br>R: CCAATCCTAATGATCCGCTG	1AS, -2.3 cM	
Xgpw7072	F: AATGCAGCGATGTCTTGCTA<br>R: ACTGTTGGGGTTGTTGTTGC	1AS, 4.8 cM	
Xgpw2276	F: ATAGGGTTCTTCTGTGCCCC<br>R: ACCCACAGTTGAACTTGGG	1AS, 13.8 cM	
Xwmc24	F: GTGAGCAATTTTGATTATACTG<br>R: TACCCTGATGCTGTAATATGTG	1AS, 35.3 cM	
Xpsp3000	F: GCAGACCTGTGTCATTGGTC<br>R: GATATAGTGGCAGCAGGATACG	1BS, 2.4 cM	DLGliB1
Xwmc406	F: TATGAGGGTCGGATCAATACAA<br>R: CGAGTTTACTGCAAACAAATGG	1BS, 20.0 cM	
Xwmc230	F: AGAAGCGAGCAGGTGTGTTTGA<br>R: CTGCTTCCTCCCACAACAGATG	1BS, 20.5 cM	
Xgpw4069	F: GCTCCCTTTGCGTATCATGT<br>R: GCTCTTGCTCAGAAGCGAAT	1BS, 25.6 cM	
Xwmc147	F: AGAACGAAAGAAGCGCGCTGAG<br>R: ATGTGTTTCTTATCCTGCGGGC	1DS, 17.5 cM	DLGliD1
Xgpw7082	F: AGGTACGGAGTGCAATGGAA<br>R: CCCAACAGTCAGGTCAAGGT	1DS, 23.0 cM	
Xwmc432	F: ATGACACCAGATCTAGCAC<br>R: AATATTGGCATGATTACACA	1DS, 28.0 cM	
Xcfd15	F: CTCCCGTATTGAGCAGGAAG<br>R: GGCAGGTGTGGTGATGATCT	1DS, 28.1 cM	
Xwmc336	F: GTCTTACCCCGCGATCTGC<br>R: GCGGCCTGAGCTTCTTGAG	1DS, 30.0 cM	
Xwmc222	F: AAAGGTGCGTTCATAGAAAATTAGA<br>R: AGAGGTGTTTGAGACTAATTTGGTA	1DS, 36.0 cM	
Xgpw4329	F: AGGAGGACACTTGACTGTAGCC<br>R: GTTCATGCTTTTCTTCGTCTCA	6AS, -5.2 cM	DLGliA2
Xgpw3041	F: GCGTTTTTCCTATTCCTGCA<br>R: ACACCCAAACACTCGGTCTC	6AS, 5.1 cM	
Xgwm459	F: ATGGAGTGGTCACACTTTGAA<br>R: AGCTTCTCTGACCAACTTCTCG	6AS, 5.7 cM	
Xgwm334	F: AATTTCAAAAAGGAGAGAGA<br>R: AACATGTGTTTTTAGCTATC	6AS, 8.4 cM	
Xgpw2082	F: AGCAAAATCATGCATTGAAAA<br>R: CAACAACAGAAGGTGGGTGG	6AS, 28.0 cM	
Xgpw7073	F: CAACCATTTCGACCACAACA<br>R: CTCAGGGATCTGCCATAGGT	6AS, 29.6 cM	
Xgpw7076	F: ATTCGCAACCACAACCATTT<br>R: CTCAGGGATCTGCCATAGGT	6AS, 30.6 cM	
Xgpw7592	F: CAAGACAAGGACAGCAACGA<br>R: TCAGGCGCTTTTGAAGATG	6AS, 59.9 cM	
Xgdm113	F: ACCCATCTGATATTTTGGGG<br>R: AAAATGCCCTTCCCAACC	6BS, 36.0 cM	DLGliB2
Xbarc14	F: GCGTTGTGGAAACTCAGTTTTGTTGATTTA<br>R: GCGGAAAGGAACGAAGTACATTTTGTAGA	6BS, 37.6 cM	
Xpsp3009	F: CACCGGTTTTGACACCGATAGCC<br>R: TGGGTAAGCTGCATGGGCAAACC	6BS, 38.0 cM	

Xwmc494	F: GGATCGAGTCTCAAGTCTACAA	6BS, 41.0 cM	
	R: AGAAGGAACAAGCAACATCATA		
Xgwm508	F: GTTATAGTAGCATATAATGGCC	6BS, 43.0 cM	
	R: GTGCTGCCATGATATTT		
Xbarc198	F: CGCTGAAAAGAAGTGCCGCATTATGA	6BS, 48.0 cM	
	R: CGCTGCCTTTTCTGGATTGCTTGTCA		
Xwmc737	F: CGACTAGGACTAGACGACTCTAACGG	6BS, 54.0 cM	
	R: GTCGATCACCAGAGGCATTG		
Xgpw4465	F: GGTCATTATCTGTCCCCTTGC	6DS, 23.5 cM	DLGliD2
	R: CGGTTTCAGTTTGCTACTTGC		
Xcfd42	F: AGGTTCTAGGGGGCATGTCT	6DS, 33.7 cM	
	R: GCTCTCAATGACTGCACTGG		
Xcfd132	F: CAAATGCTAATCCCCGCC	6DS, 38.4 cM	
	R: TGTAAACAAGGTCGCAGGTG		
Xcfd33	F: TACCGCAATAATCACACCCA	6DS, 55.0 cM	
	R: GGTCGATGGACTGTCCCTAA		
Xgdm127	F: ACGGGGAAATTAAAACGACC	6DS, 61.9 cM	
	R: TGAGATGGAATCGACAGAAA		
Xbarc196	F: GGTGGGTTTTATCGAATAGATTTGCT	6DS, 72.0 cM	
	R: GCGTTTCGTCAAGATTAATGCAGGTTT		

Note: F, forward primer; R, reverse primer. The primer sequences and chromosomal positions of the 36 microsatellite markers were derived from GrainGenes (http://wheat.pw.usda.gov/ggpages/SSRclub/GeneticPhysical/).

Table S7. PCR primers used for mapping the chromosomal locations of 17 gliadin genes.

Target gene	Sequence (5' - 3')	Tm (℃)	Amplicon size (bp)
Gli-α1	F: TCTCCTTCCAACAGCCTCTGC	58	180
	R: GTGCAATATGGAGGGATGTAA		
Gli-α4	F: CATCATTCTCATTAGTAGAG	58	414
	R: CTGTGAAATTGGTTGTTGTGC		
Gli-α5	F: TCCGGCCATCTCAGCAAAACT	52	187
	R: CTAGAGTTATTTTCTTCTCAT		
Gli-α7	F: ATTGCAGCAACACAACATAGT	61	345
	R: CATTGCAGTAGCGTCTGTAGG		
Gli-α8	F: ACCATCGAGCCAGGTCTCCTTG	65	137
	R: TTATTTCCTCGAACTGGGGCAA		
Gli-α9	F: GCCTCAGCAACAATATCCATCG	63	141
	R: GCCTCAGCAACAATATCCATCG		
Gli-α10	F: TCATGCTATTATTTTGCATCA	58	287
	R: ATGGCGCAATGGTGGTCGAGC		
Gli-α11	F: CATGCTATTATTTTGCATCAT	58	276
	R: ATGGCGCAATGGTGGTCGAGC		
Gli-α16	F: CAACAGCAACCACAGTATCTA	58	410
	R: TGGAAGGAGACCTGGCTCGAC		
Gli-α17	F: CATTCATGCTATTATTTTGCA	58	305
	R: ATGGCGCAATGGTGGTCGAGC		
Gli-α18	F: TGTTGCTCATGCTATTATTAT	58	320
	R: ATGGCGCAATGGTGGTCGAGC		
Gli-α20	F: CACAACATAGCGCATGGAAGG	65	205
	R: GCTGTTGGAAGGAGACCTGGG		
Gli-α24	F: AGCAAGTTCCATTGGTACAGG	60	160
	R: TGCGAATACTGTGGTTGCGGC		
Gli-α25	F: ACAACAGCCATATCCGCAGCT	65	139
	R: TGGTTGCGGTTGTGGATATGA		
Gli-γ1	F: CAGATCATGCGGCCACTATTTC	62	167
	R: CTGGCAAATGGTGCCTTGG		
Gli-γ11	F: GCAACAATGTTGCCAACAACTG	62	135
	R: AGCTGAAACAGTGGCCGCC		
Gli-ω2	F: GAGTAGCGGTATAGGCATCAGG	58	89
	R: GAACACTCCATTGACTAAACGC		

Note: F, forward primer; R, reverse primer.
